# LINC00955 suppresses colorectal cancer growth by acting as a molecular scaffold of TRIM25 and Sp1 to Inhibit DNMT3B-mediated methylation of the PHIP promoter

**DOI:** 10.1186/s12885-023-11403-2

**Published:** 2023-09-23

**Authors:** Ganglin Ren, Hongyan Li, Dan Hong, Fangyu Hu, Rongjia Jin, Shuang Wu, Wenhao Sun, Honglei Jin, Lingling Zhao, Xiaodong Zhang, Dongxiang Liu, Chuanshu Huang, Haishan Huang

**Affiliations:** 1 Zhejiang Provincial Key Laboratory of Medical Genetics, Key Laboratory of Laboratory Medicine, Ministry of Education, China, School of Laboratory Medicine and Life Sciences, Wenzhou Medical University, Wenzhou, 325035 Zhejiang China; 2 Jiaxing Center for Disease Control and Prevention, Jiaxing, 314050 Zhejiang China; 3 The First Affiliated Hospital of Wenzhou Medical University, Wenzhou, 325035 Zhejiang China; 4 Center for Chemical Biology, Shanghai Institute of Materia Medica, Chinese Academy of Sciences, Shanghai, 201203 China

**Keywords:** LINC00955, Colorectal cancer, Cell cycle, CDK2, PHIP, Sp1

## Abstract

**Background:**

Long non-coding RNAs play an important role in the development of colorectal cancer (CRC), while many CRC-related lncRNAs have not yet been identified.

**Methods:**

The relationship between the expression of LINC00955 (Long Intergenic Non-protein Coding RNA 955) and the prognosis of colorectal cancer patients was analyzed using the sequencing results of the TCGA database. LINC00955 expression levels were measured using qRT-PCR. The anti-proliferative activity of LINC00955 was evaluated using CRC cell lines in vitro and xenograft models in nude mice in vivo. The interaction of TRIM25-Sp1-DNMT3B-PHIP-CDK2 was analyzed by western blotting, protein degradation experiment, luciferase, RNA-IP, RNA pull-down assays and immunohistochemically analysis. The biological roles of LINC00955, tripartite motif containing 25 (TRIM25), Sp1 transcription factor (Sp1), DNA methyltransferase 3 beta (DNMT3B), pleckstrin homology domain interacting protein (PHIP), cyclin dependent kinase 2 (CDK2) in colorectal cancer cells were analyzed using ATP assays, Soft agar experiments and EdU assays.

**Results:**

The present study showed that LINC00955 is downregulated in CRC tissues, and such downregulation is associated with poor prognosis of CRC patients. We found that LINC00955 can inhibit CRC cell growth both in vitro and in vivo. Evaluation of its mechanism of action showed that LINC00955 acts as a scaffold molecule that directly promotes the binding of TRIM25 to Sp1, and promotes ubiquitination and degradation of Sp1, thereby attenuating transcription and expression of DNMT3B. DNMT3B inhibition results in hypomethylation of the PHIP promoter, in turn increasing PHIP transcription and promoting ubiquitination and degradation of CDK2, ultimately leading to G0/G1 growth arrest and inhibition of CRC cell growth.

**Conclusions:**

These findings indicate that downregulation of LINC00955 in CRC cells promotes tumor growth through the TRIM25/Sp1/DNMT3B/PHIP/CDK2 regulatory axis, suggesting that LINC00955 may be a potential target for the therapy of CRC.

**Supplementary Information:**

The online version contains supplementary material available at 10.1186/s12885-023-11403-2.

## Introduction

Colorectal cancer (CRC) is the third most prevalent cancer type and the second major cause of cancer-related deaths worldwide [1]. Surgical removal of CRCs is the main therapy for patients with CRC, and is frequently combined with other treatment modalities such as neoadjuvant and adjuvant chemotherapy, radiotherapy, and treatment with targeted agents [2]. These strategies, however, have not significantly improved survival rates in patients with CRC. Identifying new molecular markers and therapeutic targets, and clarifying the mechanisms underlying their effects, may provide greater understanding of the occurrence, development, and therapy of CRC.

Long non-coding RNAs (lncRNAs) are a new type of transcript encoded by the genome, but mostly not translated into protein [3]. LncRNAs are involved in a variety of cellular biological processes, including gene regulation and chromatin dynamics [4]. Aberrant expression and mutation of lncRNAs are widely associated with a variety of disease development and cell functional behaviors, including tumor proliferation [5], invasion, and metastasis [6]. LncRNAs are expected to serve as biomarkers for cancer prognosis, diagnosis, and efficacy prediction, and as therapeutic targets [7, 8]. In the past decade, a variety of lncRNAs have been found to be involved in the occurrence and development of CRC [9]. For example, lncRNA HIF1 α-As2 upregulates hypoxia inducible-factor 1α (HIF1α) expression through a ceRNA mechanism, promoting interaction of HIF1α with the RMRP promoter to activate the IGF2 signaling pathway and promote CRC progression [10]. LncRNA GLCC1 stabilizes c-myc by binding to HSP90, which in turn upregulates transcription of lactate dehydrogenase and supports survival and proliferation of CRC cells by enhancing glycolysis [11]. Although many lncRNAs are closely related to the malignant progression of CRC and have application prospects in tumor screening and detection, while many CRC-related lncRNAs have not yet been identified. Therefore, further functional lncRNAs and their regulatory mechanisms in CRC development still need to be explored.

LINC00955 (Long Intergenic Non-protein Coding RNA 955) is an intergenic lncRNA located on chromosome 4p16.3 with a full length of about 2483 nucleotides. Its biological function has not been reported. The present study was designed to assess the role of LINC00955 in the development of CRC. Evaluation of The Cancer Genome Atlas (TCGA) revealed that LINC00955 was downregulated in CRC tissues, and lower levels of LINC00955 were associated with worse survival. Overexpression of LINC00955 significantly inhibited proliferation of CRC cells in vitro and in vivo. Mechanistically, LINC00955 bound to Sp1 transcription factor (Sp1) protein to modulate the Sp1 protein level post-translationally by regulating the binding of the E3 ubiquitin ligase tripartite motif containing 25 (TRIM25) to Sp1. Downregulation of Sp1 inhibited the cell cycle and malignant proliferation of CRC cells through the DNA methyltransferase 3 beta (DNMT3B)/pleckstrin homology domain-interacting protein (PHIP)/cyclin-dependent kinase 2 (CDK2) axis. This study revealed a novel mechanism by which LINC00955 inhibits the development of CRC and provided a theoretical basis for a potential targeted therapy for CRC.

## Methods

### Plasmids, reagents, and antibodies

Fenghui Biotechnology Co., Ltd. (Hunan, China) provided the LINC00955 precursor overexpression plasmid, and MiaoLingBio provided the GFP-Sp1 plasmid (Wuhan, China). PHIP-knockdown plasmids were from Open Biosystem. HA-CDK2, HA-DNMT3B, HA-TRIM25, a set of TRIM25-targeting shRNA plasmids, the PHIP promoter-driven luciferase reporter, the DNMT3B promoter-driven luciferase reporter, and deleted LINC00955 fragments were constructed in the laboratory. Antibodies against Sp1 (9389), HA (3724), FOXO1 (2880), and FOXO3A (12,829) were sourced from CST (Boston, MA, USA). Santa Cruz Biotechnology (Dallas, TX, USA) provided antibodies against cyclin D1 (sc-20044), GFP (sc-9996), CDK2 (sc-6248), CDK4 (sc-260), CDK6 (sc-7961), Sp2 (sc-17814), and Sp3 (sc-28305). Antibodies against β-actin (Ab0011) were purchased from Abways Technology (Shanghai, China). Antibodies against tubulin (Ab7291), KLHL6 (Ab182163), and FOXC1 (Ab5079) were purchased from Abcam (Cambridge, MA, USA). Proteintech (Wuhan, China) sold antibodies against the following: PHIP (20,933–1-AP), TRIM25 (12,573–1-AP), DNMT1 (24,206–1-AP), DNMT3A (20,954–1-AP), and cyclin E2 (11,935–1-AP). Antibodies against DNMT3B (52A1018) were purchased from Novus Biologicals (USA).

### Human samples and cell lines

A total of 75 pairs of CRC and adjacent normal human tissues were provided by the First Affiliated Hospital of Wenzhou Medical University. Each specimen was divided into three parts when sampled: one part was confirmed as CRC by pathological examination, the second part was used to extract RNA and synthesize cDNA, and the third part was fixed in formalin, embedded in paraffin, and stored at room temperature. The human CRC cell lines HCT116 (CBP60028 COBIOER, Nanjing, China), HT29 (CBP60011 COBIOER), RKO (CBP60006 COBIOER), SW480 (CCL-228; ATCC, Manassas, VA, USA), CCD 841 CoN (CRL-1790, ATCC), and CCD-18Co (CRL-1459, ATCC) were cultured in 1640 medium (Gibco, 11,875–093), McCoy’s 5A 127 medium or minimum essential medium supplemented with 10% fetal bovine serum (FBS) at 37 °C in a humidified incubator with 5% CO_2_.

### Quantitative real-time PCR (qRT-PCR)

Total RNA was extracted from samples using TRIzol (Invitrogen) and reverse-transcribed using Fast SYBR Green Master Mix kit (4,385,614, Applied Biosystems). The results were normalized to those of β-actin. The following primers were used: human CDK2 (forward, 5′-GGC ATT CCT CTT CCC CTC ATC AA-3′; reverse, 5′-CTC CAA AAG CTC TGG CTA GTC CA-3′); human LINC00955 (forward, 5′-CGT CGC CAA CGC CCC TAG GAC-3′; reverse, 5′-CAC CCG GAA GTC TCA TGT GGA-3′); human PHIP (forward, 5′-ACA GAC TTG AGC GAC TTG TT-3′; reverse, 5′-GGT AGC TAA CAA CCT CCC AT-3′); human DNMT3B (forward, 5′-AGG GAA GAC TCG ATC CTC GTC-3′; reverse, 5′-GTG TGT AGC TTA GCA GAC TGG-3′); human Sp1 (forward, 5′-AGC AGC AGC AAC ACC ACT CTC AC-3′; reverse, 5′-TCA TCA TGT ATT CCA TCA CCA CCA G-3′); and human β-actin (forward, 5′-TGG ATG ATG ATA TCG CCG CG-3′; reverse, 5′-GTG CTC GAT GGG GTA CTT CAG-3′).

### Western blotting

Tissues or cells were sonicated, their protein concentrations were measured, and equal aliquots were loaded onto SDS-PAGE gels, which were electrophoresed. Protein samples were transferred to nylon membranes, which were then incubated in 5% non-fat milk for 1 h to avoid non-specific binding. The appropriately diluted primary antibody was applied to the membranes, followed by three washes with TBS, incubation with a tagged secondary antibody for 3 h at 4 °C, and three more washes with TBS. Finally, the membranes were exposed to film. Antibodies against Sp1, HA, FOXO1, FOXO3A, FOXC1, TRIM25, and DNMT1 were diluted 1:1000. Antibodies against cyclin D1, GFP, CDK2, CDK4, CDK6, Sp2, Sp3, cyclin E2, and DNMT3B were diluted 1:500. Antibodies against β-actin, α-tubulin, and DNMT3A were diluted 1:10,000. The anti-KLHL6 antibody was diluted 1:1500. The anti-PHIP antibody was diluted 1:2000.

### IHC

Tissue samples were cut and embedded in paraffin, and then baked, dewaxed, and hydrated. Antigen retrieval was performed by dropwise addition of 3% H_2_O_2_. After blocking non-specific binding with 5% BSA, the samples were treated with the appropriately diluted primary antibody overnight at 4 °C. The samples were then washed and incubated for 1 h with the appropriate biotinylated secondary antibody, and then SABC (SA1022, Boster Bio Engineering Company, Wuhan, China) was added dropwise. Samples were developed with DAB (59,718, Abcam) and examined by light microscopy. The final hematoxylin staining was terminated with ddH_2_O. Antibodies against CDK2 (Proteintech, 10,122–1-AP, 1:250), PHIP (Bioworld, BS8775, 1:100), DNMT3B (Proteintech, 26,971–1-AP, 1:250), and Sp1 (Cell Signaling Technology, 9389, 1:2000) were used for IHC.

### Experimental animals

Female BALB/C-nu nude mice weighing 15 ± 0.5 g obtained from GemPharmatech (license number: SCXK [SU] 2018–0008; Nanjing, Jiangsu, China) were raised in the SPF facility of Wenzhou Medical University for experimental animals. The Wenzhou Medical University Experimental Animal Ethics Committee approved all animal research. Twelve female BALB/c-nu nude mice were randomly split into two groups of six. Each mouse was subcutaneously injected with 5 × 10^6^ HCT116 (Vector) or HCT116 (LINC00955) cells in 100 μL of media, and the injection was performed slowly and at a constant speed. Three weeks later, the mice were euthanized by injecting excessive pentobarbital sodium. The tumors were dissected out, photographed, and weighed.

### Cell cycle analysis

Cells in logarithmic growth phase were trypsinized, resuspended, and added to the wells of 6-well plates. The cells were grown with 0.1% FBS medium for 12 h after adhering to the plates, and subsequently with the matching 10% FBS complete medium for 12 h. The cells were stored in a 4 °C refrigerator for 12–24 h after being digested in EP tubes with 70% pre-cooled alcohol. The cell pellet was obtained by centrifugation at 1000 × g for 5 min, then the cell pellet was washed with precooled PBS, and the cells were stained with 30 μL RNaseA and 120 μL PI staining solution. The cell suspensions were then assayed by flow cytometry.

### RNA pull-down assay and mass spectrometry

RNA pull-down kits were used to perform RNA pull-down experiments (Bes5102; BersinBio, China). Secondary structures of the corresponding mass of biotin-labeled target RNA probes and NC probes were formed. Two RNase-free centrifuge tubes each received 40 μL of streptavidin magnetic beads. RNA probes (about 100 μL) were added to form secondary structures with the magnetic beads, tubes were centrifuged and incubated at 25 °C, and the supernatants were discarded. Aliquots containing 2 × 10^7^ cells were washed, with 100 μL of supernatant considered the input group. The probe-magnetic bead complex was mixed with the cell magnetic bead complex and the cell lysate, followed by incubation with rotation for 2 h. The beads were collected and washed for 5 min. The magnetic beads were subsequently mixed with 60 μL of protein elution buffer, and incubated at 37 °C for 2 h. The supernatants were transferred to new centrifuge tubes. A 15 μL aliquot of each protein sample was loaded onto SDS-PAGE gels for western blotting. The gels were stained for 2 h in Coomassie brilliant blue staining solution, washed with ddH_2_O, and subjected to mass spectrometry.

### Methylation-specific PCR

DNA extracted from peripheral blood leukocytes was methylated by the M.SssI (M0226S; New England Biolabs, USA) enzyme to obtain fully methylated DNA. Sodium bisulfite conversion was performed using EZ DNA Methylation-Gold kits (D5005; Zymo Research, USA). Specific primers were used for PCR amplification and nucleic acid electrophoresis as follows: MF: 5′-GGG TGG GGG TTT TAT TGT GTC G-3′; MR: 5′-GAA TCC CTC CGC CGC A-3′; UF: 5′-GGG TGG GGG TTT TAT TGT GTT G-3′; and UR: 5′-AAA TCC CTC CAC CAC A-3′.

### RNA-IP assay

HCT116 (LINC00955) cells cultured in a 10 cm dish to 70–80% confluence were lysed using the buffer provided in the RNA Immunoprecipitation Kit (Bes5101; BersinBio, China). Each cell lysate sample was divided into three aliquots, with 0.8 mL used for IP, 0.8 mL for IgG assays, and 0.1 mL as input. IP and IgG samples were supplemented with specific antibodies. Each sample received 20 μL of carefully balanced protein A/G beads. The beads were recovered by centrifugation and then incubated at 55 °C for 1 h with polysome elution buffer. RNA was eluted and reverse-transcribed, and qPCR was performed.

### IP

A Myc-Ub IP assay was performed. Briefly, PHIP-knockdown HCT116 (LINC00955) and RKO (LINC00955) cells and TRIM25 knockdown HCT116 (LINC00955) and RKO (LINC00955) cells were grown in 10 cm plates to 70–80% confluence, followed by co-transfection with Myc-Ub and HA-CDK2 or Myc-Ub and GFP-Sp1. Eight hours later, the medium was changed, followed by incubation for another 12 h. MG132 (10 µM) was added to the cells for 8 h. Each protein sample received an anti-Myc antibody before being incubated at 4 °C for 12 h. The samples were mixed with agarose beads (sc2003; Santa Cruz, USA) and incubated for 3 h at 4 °C. The agarose beads were collected and washed 5–6 times. The protein samples were analyzed by western blotting after addition of 60 µL of elution buffer.

### Prediction of Sp1 and TRIM25 binding regions within LINC00955

The genomic sequence of human LINC00955 was obtained from the nucleotide database of the National Library of Medicine (sequence ID: NR_040045.1). The secondary structure of LINC00955 was predicted using RNAfold, using the options of minimum free energy and partition function, while avoiding isolated base pairs. The RNA binding domains in the transcription factor Sp1 (entry ID: P08047) and the E3 ubiquitin/ISG15 ligase TRIM25 (entry ID: Q14258) were predicted from their three dimensional structures modeled by AlphaFold. Specifically, folds similar to two structural domains in Sp1 (i.e., amino acids 429–558 and 626–714) and three domains in TRIM25 (i.e., amino acids 1–84, 105–190, and 431–630) were searched in the Protein Data Bank using the Dali web server. LINC00955 was aligned with the nucleotides in 1MEY using Clustal Omega.

### Statistical analysis

The mean ± standard deviation (± SD) of three separate experiments are used to represent all experimental data and were compared using t-tests. *p* < 0.05 was deemed statistically significant.

## Results

### Downregulation of LINC00955 in CRC tissues and cells, and LINC00955 suppression of CRC cell growth in vitro and in vivo

**Fig. 1:**
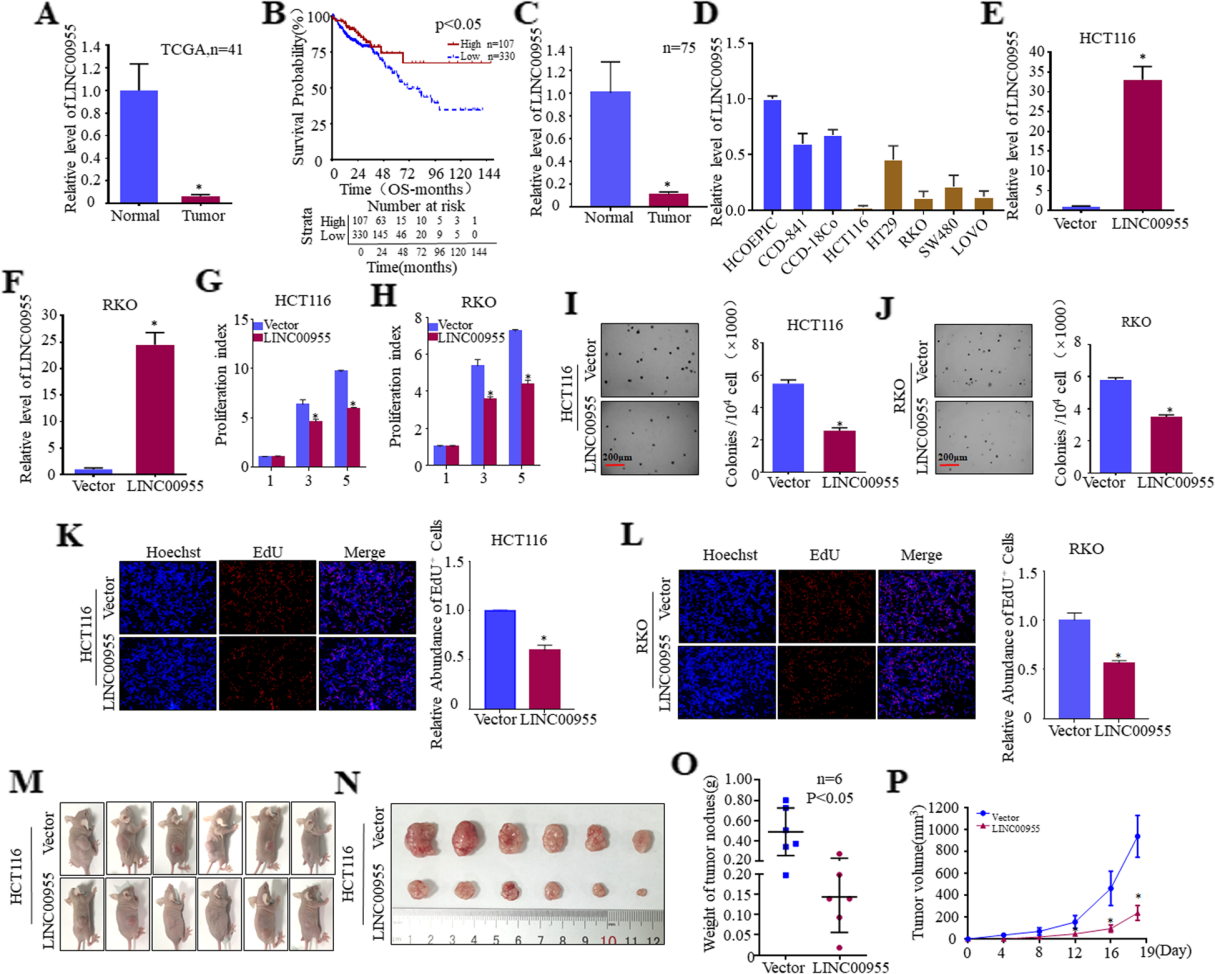
Expression of LINC00955 is considerably lower in CRC tissues, and it inhibits growth of CRC cells in vitro and in vivo. **A** Expression of LINC00955 in clinical samples from the TCGA database. **B** Correlation between expression of LINC00955 in the TCGA database and the survival rate of CRC patients. **C**, **D** qPCR assays of the expression of LINC00955 in (**C**) primary CRC and adjacent normal colorectal tissue samples, and (**D**) in normal colorectal and CRC cell lines. **E**, **F** LINC00955 expression in HCT116 and RKO cells stably transfected with LINC00955, as determined by qPCR. **G**, **H** Effects of LINC00955 on proliferation of CRC cells, as determined by ATP assays. **I**, **J** Soft agar experiments were performed to analyze the effects of LINC00955 on the growth of CRC cells. **K**, **L** Effects of LINC00955 on the DNA replication activity of HCT116 and RKO cells, as determined by EdU assays. **M** HCT116 cells were injected into nude mice, which were then imaged after developing tumors. **N** Photographs of excised tumors. **O** Comparison of tumor weight in two sets of nude mice. **P** Volumes of tumors excised from the two groups of nude mice over 19 days. An asterisk (*) indicates a significant difference (*p* < 0.05)

The potential role of LINC00955 in development of CRC was investigated by analyzing its expression in samples from the TCGA database. Expression of LINC00955 was markedly lower in CRC tumor tissue than in normal colon tissue (Fig. 1A). Kaplan–Meier analysis revealed that downregulation of LINC00955 was associated with poor prognosis in patients with CRC (Fig. 1B). Downregulation of LINC00955 was confirmed by qPCR assays of 75 clinical samples (Fig. 1C). LINC00955 levels were lower in human CRC cell lines, including HCT116, HT29, RKO, SW480, and LOVO cells, than in normal human colorectal cell lines (Fig. 1D).

The possible role of LINC00955 in CRC was further explored by constructing stable CRC cell lines bearing LINC00955, including HCT116 (Vector, LINC00955) and RKO (Vector, LINC00955) cells (Fig. 1E, F), and testing the effects of LINC00955 on proliferation of these cells. LINC00955 significantly reduced the growth rates of monolayers of HCT116 and RKO cells (Fig. 1G, H), and reduced their anchorage-independent growth (Fig. 1I, J). EdU assays showed that LINC00955 significantly inhibited DNA replication in CRC cells (Fig. 1K, L). To better assess the effects of LINC00955 on CRC cell proliferation, HCT116 cells were injected under the skin of nude mice, and tumor growth was monitored. Overexpression of LINC00955 led to a significant reduction in the size and weight of subcutaneous tumors in nude mice (Fig. 1M–P). These findings demonstrate that LINC00955 prevents CRC cells from proliferating malignantly both in vivo and in vitro.

### LINC00955 induces cell cycle arrest of CRC cells by inhibiting CDK2

**Fig. 2:**
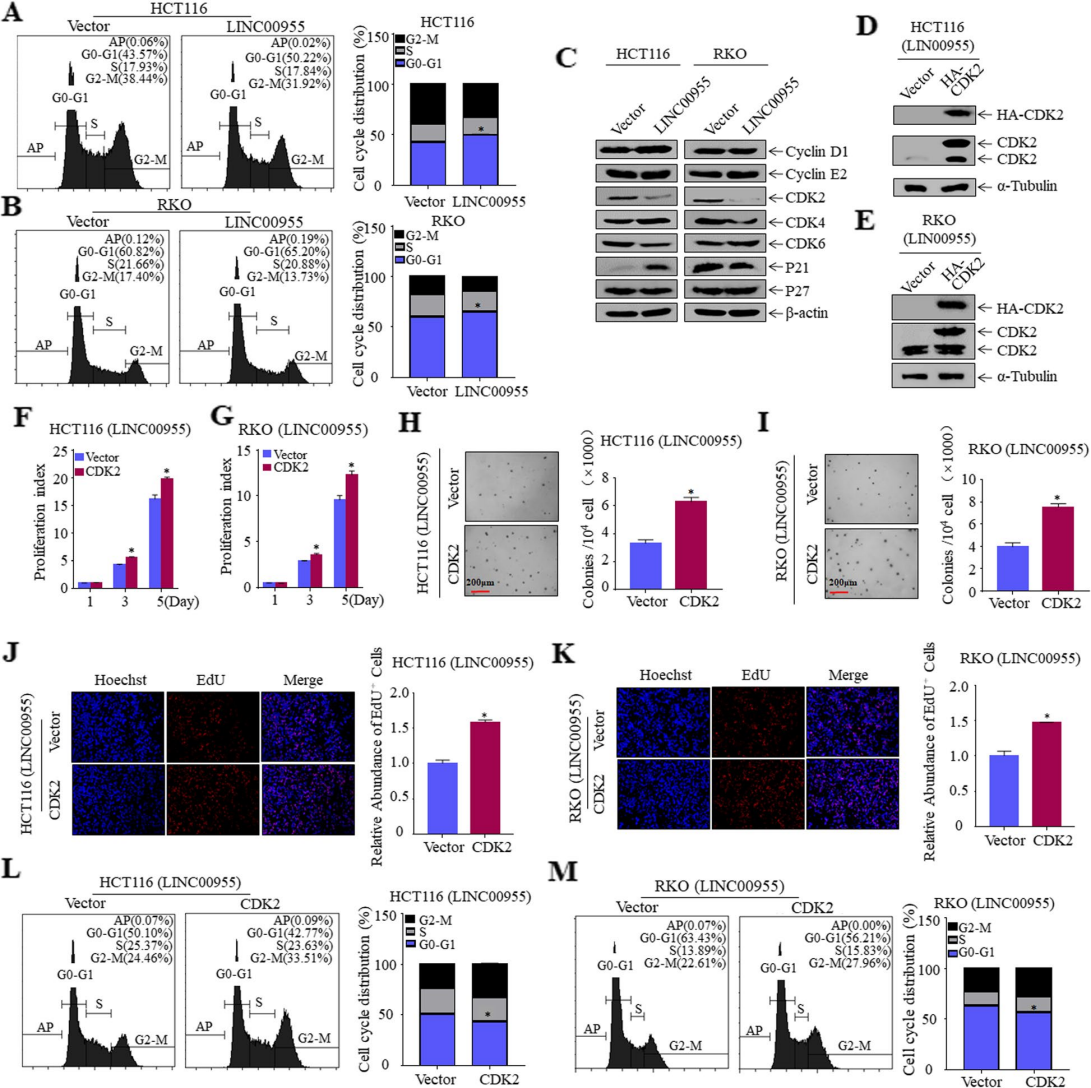
LINC00955 inhibits the proliferation of CRC cells by downregulating expression of CDK2 and inducing cell arrest at G0/G1 phase. **A**, **B** Flow cytometric investigation of the impact of LINC00955 on the cell cycle of CRC cells. **C** Western blotting, showing expression of important proteins associated with the G0-G1 phase. **D**, **E** The efficiency of transfecting HA-CDK2 and its control plasmids into HCT116 (LINC00955) and RKO (LINC00955) cells was evaluated using western blotting. **F**, **G** Effects of CDK2 overexpression on cell proliferation, as measured by ATP assays, in (**F**) HCT116 (LINC00955) and (**G**) RKO (LINC00955) cells. **H**, **I** Effects of CDK2 overexpression on growth of (**H**) HCT116 (LINC00955) and (**I**) RKO (LINC00955) cells, as determined by soft agar assays. (**J**, **K**) Effects of CDK2 overexpression on the DNA replication activity of (**J**) HCT116 (LINC00955) and (**K**) RKO (LINC00955) cells, as determined by EdU assays. **L**, **M** Flow cytometric analysis of the effects of CDK2 overexpression on the cell cycle in (**L**) HCT116 (LINC00955) and (**M**) RKO (LINC00955) cells. An asterisk (*) indicates a significant difference (*p* < 0.05)

The cell cycle is the main physiological process that drives cell growth, as well as a crucial factor in the uncontrolled proliferation of tumor cells. The ability of LINC00955 to affect the cell cycle of CRC cells was therefore evaluated by flow cytometry. Overexpression of LINC00955 induced G0/G1 phase arrest of HCT116 and RKO cells (Fig. 2A, B). These results imply that the ability of LINC00955 to mediate CRC cell proliferation is due to its effect on cell cycle progression. To clarify the mechanism by which LINC00955 induces G0/G1 phase arrest, G0/G1 phase-related proteins in these cells were examined using western blotting. LINC00955 substantially downregulated expression of CDK2 in HCT116 and RKO cells (Fig. 2C), suggesting that LINC00955 may inhibit malignant proliferation of CRC cells by inhibiting CDK2 expression. To verify this hypothesis, HCT116 (LINC00955) and RKO (LINC00955) cells were stably transfected with the HA-labeled CDK2 plasmid (Fig. 2D, E). ATP, soft agar, and EdU assays showed that, compared with control cells, overexpression of CDK2 rescued the growth ability of CRC cells (Fig. 2F–K). Additionally, flow cytometry demonstrated that overexpression of CDK2 drastically reduced the ability of LINC00955 to cause cell cycle arrest in HCT116 and RKO cells (Fig. 2L, M). Collectively, these findings indicate that CDK2 is an important downstream effector of LINC00955.

### LINC00955 mediates degradation of CDK2 through E3 ligase PHIP

**Fig. 3:**
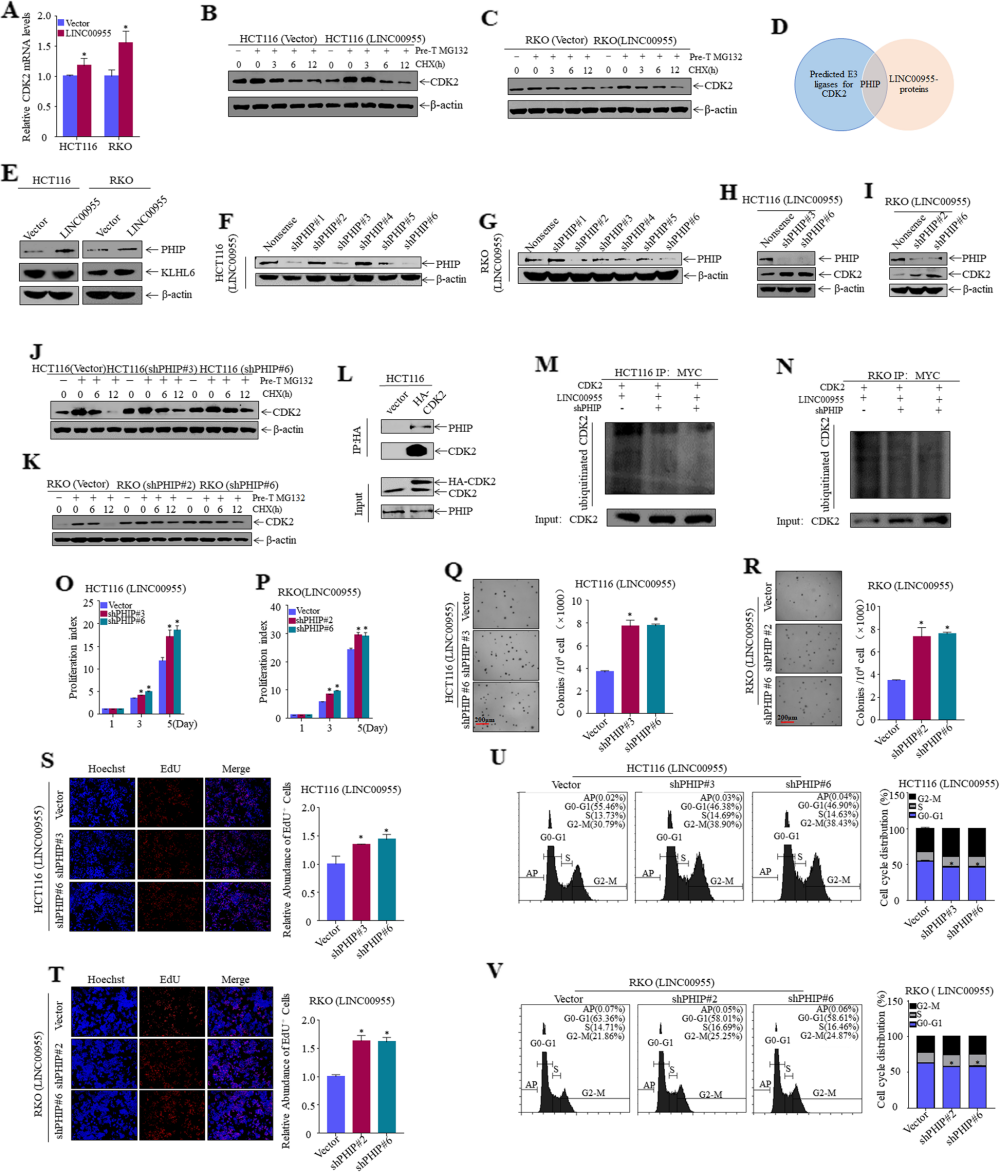
LINC00955 promotes ubiquitination and degradation of CDK2, and inhibits proliferation of CRC cells by promoting expression of PHIP. **A** qPCR assay of CDK2 mRNA levels in HCT116 (Vector, LINC00955) and RKO (Vector, LINC00955) cells. **B**, **C** Degradation of CDK2 protein in (**B**) HCT116 (Vector, LINC00955) and (**C**) RKO (Vector, LINC00955) cells treated with MG132 and CHX, as determined by western blotting. **D** Venn diagram analysis of the E3 ligase controlling CDK2 protein degradation. **E** Expression of KLHL6 and PHIP in HCT116 (Vector, LINC00955) and RKO (Vector, LINC00955) cells according to western blotting. **F**, **G** Transfection efficiency of shPHIP in (**F**) HCT116 (LINC00955) and (**G**) RKO (LINC00955) cells according to western blotting. **H**, **I** Expression of CDK2 in (**H**) HCT116 (LINC00955) and (**I**) RKO (LINC00955) cells after PHIP knockdown, as determined by western blotting. **J**, **K** Degradation rate of CDK2 in (**J**) HCT116 (LINC00955) and (**K**) RKO (LINC00955) cells after PHIP knockdown, as determined by western blotting. **L** The connection between PHIP and CDK2, as determined by Co-IP assays. **M**, **N** Effect of PHIP knockdown on CDK2 ubiquitination in (**M**) HCT116 (LINC00955) and (**N**) RKO (LINC00955) cells, as determined by western blotting after ubiquitin-IP assay. **O**, **P** ATP assays were performed to assess the impact of PHIP knockdown on the growth of (**O**) HCT116 (LINC00955) and (**P**) RKO (LINC00955) cells. **Q**, **R** Effect of PHIP knockdown on growth of (**Q**) HCT116 (LINC00955) and (**R**) RKO (LINC00955) cells, as determined by soft agar assays. **S**, **T** Effect of PHIP knockdown on the DNA replication activity of (**S**) HCT116 (LINC00955) and (**T**) RKO (LINC00955) cells, as determined by EdU assays. **U**, **V** Effect of CDK2 knockdown on the cell cycle in (**U**) HCT116 (LINC00955) and (**V**) RKO (LINC00955) cells, as determined by flow cytometry. An asterisk (*) indicates a significant difference (*p* < 0.05)

To further investigate the specific molecular mechanisms by which LINC00955 regulates CDK2, the ability of LINC00955 to affect CDK2 mRNA levels was assessed by qPCR. LINC00955 upregulated the levels of CDK2 mRNA expression in HCT116 and RKO cells, indicating that LINC00955 did not downregulate CDK2 protein expression at the mRNA level (Fig. 3A). Next, we assessed whether LINC00955 downregulated CDK2 expression by promoting the ubiquitinated degradation. We already knew that the half-life of CDK2 protein was about 3 h [12]. Therefore, we treated HCT116 (Vector, LINC00955) and RKO (Vector, LINC00955) cells with MG132 and cycloheximide (CHX) and then identified expression of CDK2 by western blotting. The rate of CDK2 protein degradation in HCT116 (LINC00955) and RKO (LINC00955) cells was significantly higher than in HCT116 (Vector) and RKO (Vector) (Fig. 3B, C). These results reveal that LINC00955 downregulates CDK2 by inducing protein degradation.

Next, to explore whether LINC00955 regulates ubiquitination and degradation of CDK2 by regulating E3 ligase, we used western blotting to detect the important E3 ligase KLHL6, which recognizes CDK2 [12]. Expression of KLHL6 did not change significantly in HCT116 (Vector, LINC00955) and RKO (Vector, LINC00955) cells (Fig. 3E). Next, we predicted E3 ligases that are involved in CDK2 degradation using the UbiBrowser database. We also identified an E3 ligase regulated by LINC00955 by quantitative proteomic analysis. We then combined the site prediction data with the proteomic analysis results. The findings showed that PHIP may be involved in regulation of degradation of ubiquitinated CDK2 by LINC00955 (Fig. 3D). PHIP expression was detected using western blotting, and the results indicated that it was elevated in HCT116 (LINC00955) and RKO (LINC00955) cells (Fig. 3E). To further explore whether LINC00955 regulates ubiquitination and degradation of CDK2 by regulating the E3 ligase PHIP that specifically recognizes CDK2, PHIP expression was knocked down in HCT116 (LINC00955) and RKO (LINC00955) cells (Fig. 3F, G), and CDK2 expression was evaluated by western blotting. Knockdown of PHIP significantly upregulated CDK2 expression (Fig. 3H, I). Moreover, the effect of LINC00955 on degradation of CDK2 protein was assessed after knocking down PHIP in HCT116 (LINC00955) and RKO (LINC00955) cells. The results showed that knocking down PHIP significantly reduced the effect of LINC00955 on degradation of CDK2 protein (Fig. 3J, K), indicating that LINC00955 regulates ubiquitination and degradation of CDK2 by regulating the E3 ligase PHIP. To determine whether PHIP binds to CDK2, these mixtures were incubated with HA-beads, which pull-down the HA protein by immunoprecipitation (IP). The results showed that PHIP was present in the immune complex (Fig. 3L), indicating that PHIP binds to CDK2 and triggers its degradation. To further explore whether PHIP is involved in ubiquitination of the CDK2 protein, Myc-Ub and HA-CDK2 were co-transfected into PHIP-knockdown HCT116 (LINC00955) and RKO (LINC00955) cells, followed by ubiquitin-IP. The experiment found that knockdown of PHIP substantially decreased the ubiquitination level of CDK2 (Fig. 3M, N). Knocking down PHIP reduced the ability of LINC00955 to suppress the growth of CRC cells, as demonstrated by ATP, soft agar, and EdU assays (Fig. 3O–T). Flow cytometry also showed that knocking down PHIP significantly weakened the ability of LINC00955 to arrest the cell cycle of HCT116 and RKO cells (Fig. 3U, V). Collectively, these findings suggest that LINC00955 promotes ubiquitination and degradation of CDK2 by upregulating expression of PHIP, thereby inhibiting proliferation of CRC cells.

### LINC00955 downregulates the DNA methylation level of the PHIP promoter, thereby increasing promoter activity and PHIP expression

**Fig. 4:**
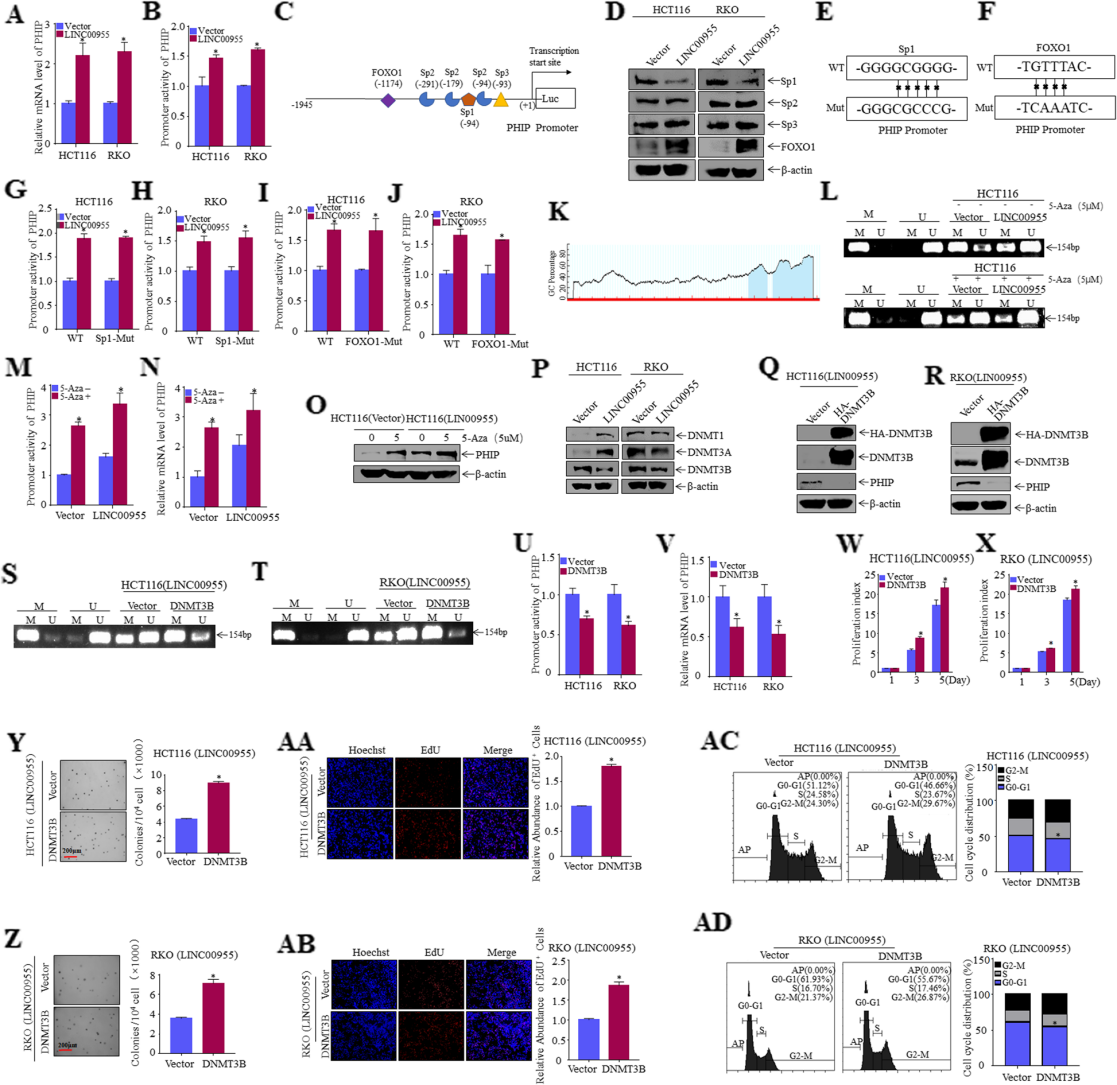
LINC00955 inhibits PHIP promoter methylation by downregulating expression of the DNA methyltransferase DNMT3B. **A** PHIP mRNA levels in HCT116 (Vector, LINC00955) and RKO (Vector, LINC00955) cells, as determined by qPCR. **B** PHIP promoter levels in HCT116 (Vector, LINC00955) and RKO (Vector, LINC00955) cells, as determined by dual-luciferase reporter assays. **C** Possible transcription factors in the promoter region of PHIP, predicted from the JASPAR database. **D** Western blotting was performed to examine expression of transcription factors in HCT116 and RKO (Vector, LINC00955) cells. **E** Construction of a Sp1 mutant PHIP promoter-driven luciferase reporter. **F** Construction of a FOXO1 mutant PHIP promoter-driven luciferase reporter. **G**, **H** Wild-type and Sp1 mutant PHIP promoter-driven luciferase reporters were transferred into (**G**) HCT116 (Vector, LINC00955) and (**H**) RKO (Vector, LINC00955) cells, and their promoter activity was determined. **I**, **J** Wild-type and FOXO1 mutant PHIP promoter-driven luciferase reporters were transferred into (**I**) HCT116 (Vector, LINC00955) and (J) RKO (Vector, LINC00955) cells, and their promoter activity was determined. **K** The MethPrimer website predicted the CpG island in the PHIP promoter. **L**–**O** HCT116 (Vector, LINC00955) cells were pretreated with the methylation inhibitor 5-Aza, and the effects of LINC00955 were tested on (**L**) the methylation and non-methylation levels of the PHIP promoter region (determined by MSP), (**M**) PHIP promoter levels (determined by dual-luciferase reporter assays), (**N**) PHIP mRNA levels (determined by qPCR), and (**O**) PHIP protein levels (analyzed using western blotting). **P** Expression of DNA methyltransferase, as determined by western blotting. **Q**, **R** The transfection efficiency of HA-DNMT3B and its control plasmids was analyzed by western blotting in (**Q**) HCT116 (LINC00955) and (**R**) RKO (LINC00955) cells. **S**, **T** Effects of DNMT3B overexpression on PHIP promoter methylation in (**S**) HCT116 (LINC00955) and (**T**) RKO (LINC00955) cells, as determined by MSP assays. **U** Effects of DNMT3B overexpression on PHIP promoter activity in HCT116 (LINC00955) and RKO (LINC00955) cells, as determined by dual-luciferase reporter assays. **V** Effects of DNMT3B overexpression on PHIP mRNA levels in HCT116 (LINC00955) and RKO (LINC00955) cells, as determined by qPCR assays. **W**, **X** Effects of DNMT3B overexpression on proliferation of (**W**) HCT116 (LINC00955) and (**X**) RKO (LINC00955) cells, as determined by ATP assays. **Y**, **Z** Soft agar tests were performed to investigate the effects of DNMT3B overexpression on the growth of (**Y**) HCT116 (LINC00955) and (**Z**) RKO (LINC00955) cells. **AA**, **AB** Effects of DNMT3B overexpression on the DNA replication activity of (**AA**) HCT116 (LINC00955) and (**AB**) RKO (LINC00955) cells, as determined by EdU assays. **AC**, **AD** Effects of DNMT3B overexpression the cell cycle in (**AC**) HCT116 (LINC00955) and (**AD**) RKO (LINC00955) cells, as determined by flow cytometry. An asterisk (*) indicates a significant difference (*p* < 0.05)

Analysis of the effects of LINC00955 on the regulation of PHIP mRNA demonstrated that PHIP mRNA levels were considerably higher in HCT116 (LINC00955) and RKO (LINC00955) cells than in HCT116 (Vector) and RKO (Vector) cells (Fig. 4A). A PHIP promoter-driven luciferase reporter was transfected into HCT116 (Vector, LINC00955) and RKO (Vector, LINC00955) cells to determine whether LINC00955 regulates PHIP expression at the transcriptional level. LINC00955 significantly enhanced PHIP promoter activity in CRC cells (Fig. 4B). JASPAR analysis to predict transcription factors that regulate PHIP identified multiple probable transcription factor binding sites in the promoter region of PHIP, including sites for Sp1, Sp2, Sp3, and FOXO1 (Fig. 4C). A truncated PHIP promoter-driven luciferase reporter was constructed (Figure S1A), with the activity of the PHIP promoter region being altered by dual-luciferase reporter assays. This experiment revealed that *PHIP-2* is the critical region responsible for the change of promoter activity (Figure S1B, C). Western blotting to assess expression of transcription factors in CRC cells showed that expression of FOXO1 was drastically upregulated, whereas the expression of Sp1 was drastically downregulated in HCT116 (LINC00955) and RKO (LINC00955) cells (Fig. 4D). These findings suggest that FOXO1 and Sp1 may be transcription factors that regulate the PHIP promoter.

To determine whether PHIP upregulation is caused directly by the transcription factors Sp1 and FOXO1, the mutation binding sites of Sp1 and FOXO1 were determined [13, 14], and a series of PHIP promoter-driven luciferase reporters harboring Sp1 and FOXO1 mutants was constructed (Fig. 4E, F). Wild-type and mutant-type PHIP promoter-driven luciferase reporters were transferred into HCT116 (Vector, LINC00955) and RKO (Vector, LINC00955) cells, and their promoter activity was measured. The activity of wild-type and mutant-type PHIP promoters did not differ significantly (Fig. 4G–J), indicating that LINC00955 does not promote transcription of PHIP by influencing transcription factor expression.

DNA methylation is an epigenetic modification that can play an important role in control of gene expression in mammalian cells, with gene silencing caused by abnormal promoter hypermethylation being one of the mechanisms leading to downregulation of tumor suppressor genes, and occurrence and progression of cancers [15, 16]. Bioinformatics software predicted that CpG islands are present in the PHIP promoter region, which was confirmed in this study (Fig. 4K). To detect alterations in the methylation level of the PHIP promoter region, methylation-specific PCR (MSP) experiments were performed using primers constructed based on the location of the CpG islands. After overexpression of LINC00955 in HCT116 cells, the DNA methylation level in the PHIP promoter region was significantly reduced (Fig. 4L), suggesting that LINC00955 promotes PHIP promoter activity by affecting DNA methylation in the PHIP promoter region. To test this hypothesis, MSP was performed after treating HCT116 (Vector, LINC00955) cells with the DNA methylation inhibitor 5-Aza, with findings showing that addition of 5-Aza suppressed the level of methylation of the PHIP promoter region (Fig. 4L). Similarly, dual-luciferase reporter, qPCR, and western blotting assays showed that 5-Aza treatment increased PHIP promoter, mRNA, and protein levels (Fig. 4M–O). LINC00955 may promote PHIP promoter activity by affecting DNA methylation in the PHIP promoter region. To determine the specific mechanism by which LINC00955 regulates DNA methylation of the PHIP promoter, expression of the related DNMT1, DNMT3A, and DNMT3B was measured by western blotting. DNMT3B levels were lower in HCT116 (LINC00955) and RKO (LINC00955) cells than in their respective control cells (Fig. 4P). DNMT3B was overexpressed in HCT116 (LINC00955) and RKO (LINC00955) cells (Fig. 4Q, R), and MSP assays detected changes in PHIP promoter methylation after overexpression of DNMT3B, suggesting that overexpression of DNMT3B can significantly increase the level of PHIP promoter methylation (Fig. 4S, T). Overexpression of DNMT3B significantly reduced PHIP promoter activity and mRNA and protein levels (Fig. 4U, V, Q, R). Additionally, ATP, soft agar, and EdU assays demonstrated that LINC00955 restricted CRC cell proliferation by repressing the expression of DNMT3B (Fig. 4W, X, Y, Z, AA, AB). Moreover, flow cytometry showed that overexpression of DNMT3B dramatically weakened the ability of LINC00955 to arrest the cell cycle of HCT116 and RKO cells (Fig. 4AC, AD).

### LINC00955 inhibits transcription of DNMT3B by downregulating expression of Sp1

**Fig. 5:**
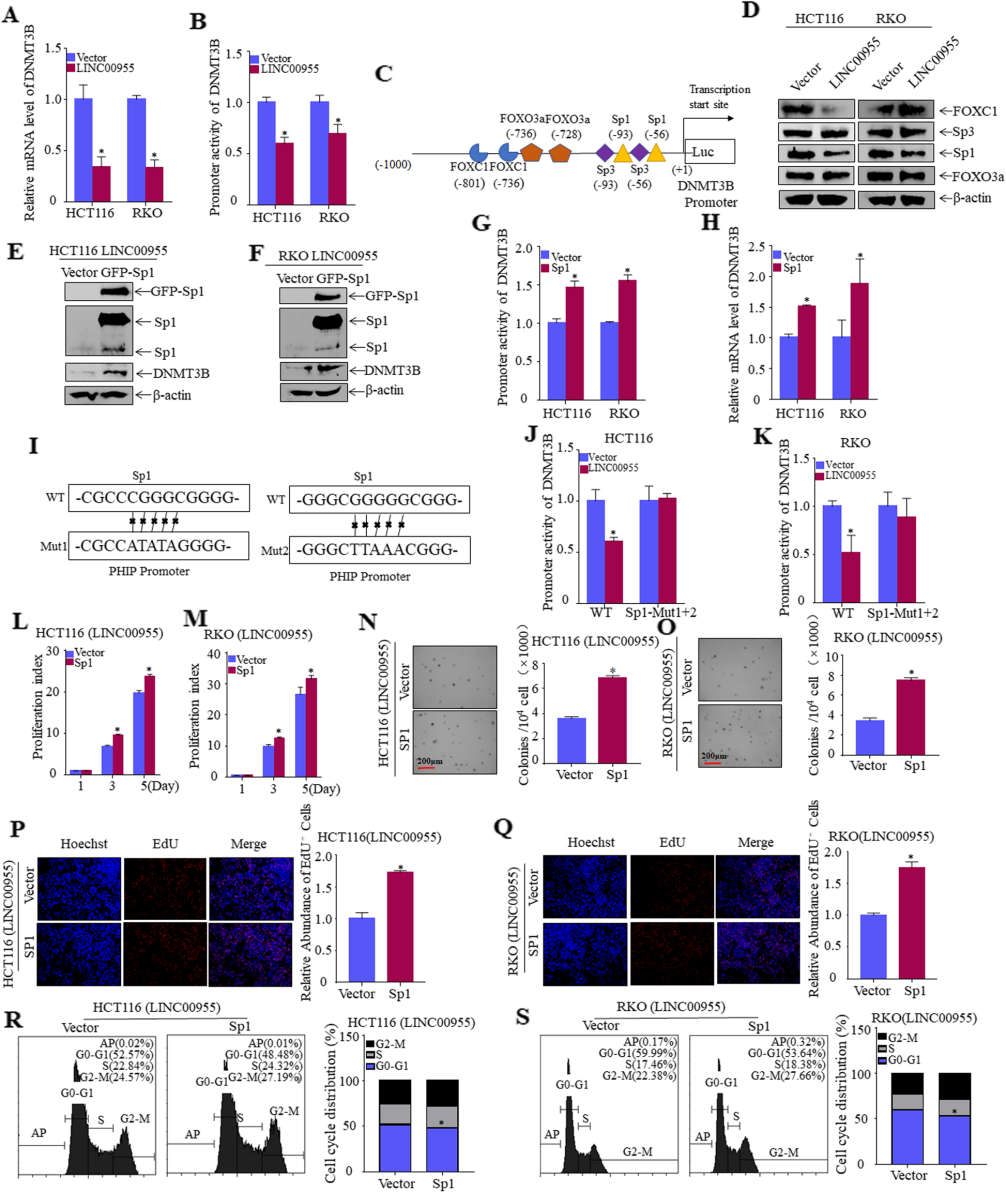
LINC00955 inhibits transcription of DNMT3B by downregulating expression of transcription factor Sp1. **A** DNMT3B mRNA levels in HCT116 (Vector, LINC00955) and RKO (Vector or LINC00955) cells, as determined by qPCR. **B** DNMT3B promoter levels in HCT116 (Vector, LINC00955) and RKO (Vector, LINC00955) cells, as determined by dual-luciferase reporter assays. **C** Transcription factors are present in the DNMT3B promoter region. **D** Expression of transcription factors in HCT116 (Vector, LINC00955) and RKO (Vector, LINC00955) cells was examined using western blotting. **E**, **F** GFP-Sp1 and its control plasmids were transfected into (**E**) HCT116 (LINC00955) and (**F**) RKO (LINC00955) cells, and stability of transfection was assessed by western blotting. **G** Dual-luciferase reporter assays showing the effects of Sp1 overexpression on DNMT3B promoter activity in HCT116 (LINC00955) and RKO (LINC00955) cells. **H** Effects of Sp1 overexpression on DNMT3B mRNA levels in HCT116 (LINC00955) and RKO (LINC00955) cells, as determined by qPCR. **I** Construction of Sp1 mutant DNMT3B promoter-driven luciferase reporters. **J**, **K** Wild-type and Sp1 mutant DNMT3B promoter-driven luciferase reporters were transferred into (J) HCT116 (Vector, LINC00955) and (**K**) RKO (Vector, LINC00955) cells, and their promoter activity was measured. (**L**, **M**) Effects of DNMT3B overexpression on proliferation of (**L**) HCT116 (LINC00955) and (**M**) RKO (LINC00955) cells, as determined by ATP assays. **N**, **O** Soft agar tests were performed to explore the effects of DNMT3B overexpression on the growth of (**N**) HCT116 (LINC00955) and (**O**) RKO (LINC00955) cells. **P**, **Q** Effects of DNMT3B overexpression on the DNA replication activity of (**P**) HCT116 (LINC00955) and (**Q**) RKO (LINC00955) cells, as determined by EdU assays. **R**, **S** Effects of DNMT3B overexpression on the cell cycle of (**R**) HCT116 (LINC00955) and (**S**) RKO (LINC00955) cells, as determined by flow cytometry. An asterisk (*) indicates a significant difference (*p* < 0.05)

To clarify the possible mechanism by which LINC00955 downregulates DNMT3B in CRC cells, the levels of DNMT3B mRNA were evaluated in HCT116 and RKO cells. DNMT3B mRNA levels were visibly lower in HCT116 (LINC00955) and RKO (LINC00955) cells than in HCT116 (Vector) and RKO (Vector) cells (Fig. 5A). The ability of LINC00955 to regulate DNMT3B promoter activity was assessed using dual-luciferase reporter assays. Overexpression of LINC00955 significantly inhibited DNMT3B promoter activity (Fig. 5B), indicating that LINC00955 downregulates expression of DNMT3B at the transcriptional level.

Transcription factors regulate expression of target genes by binding to the promoter region. The DNMT3B promoter is regulated by the transcription factors Sp1, Sp3, FOXO3A, and FOXC1 (Fig. 5C) [17–19]. Because western blotting showed that Sp1 was noticeably downregulated in HCT116 (LINC00955) and RKO (LINC00955) cells (Fig. 5D), Sp1 was stably overexpressed in HCT116 (LINC00955) and RKO (LINC00955) cells (Fig. 5E, F), and alterations in DNMT3B promoter activity and mRNA levels were determined. Sp1 overexpression significantly increased DNMT3B promoter activity and mRNA levels (Fig. 5G–H). To determine whether Sp1 was directly responsible for the downregulation of DNMT3B, the Sp1 mutation sites were determined, and DNMT3B promoter-driven luciferase reporters harboring mutant Sp1 were constructed (Fig. 5I). DNMT3B promoter activity was determined. The reduction in activity of the mutant promoter was significantly lower than that of the wild-type promoter (Fig. 5J, K), demonstrating that LINC00955 inhibited transcription of DNMT3B by downregulating expression of Sp1. ATP, soft agar, and EdU assays showed that Sp1 could restore the ability of LINC00955 to inhibit proliferation of CRC cells (Fig. 5L–Q). According to flow cytometry, overexpression of Sp1 drastically reduced the capacity of LINC00955 to arrest the cell cycle of HCT116 and RKO cells (Fig. 5R, S). Taken together, these results indicate that LINC00955 reduces expression of Sp1 to prevent CRC cell proliferation.

### LINC00955 recruits TRIM25 to degrade Sp1 via ubiquitination

**Fig. 6:**
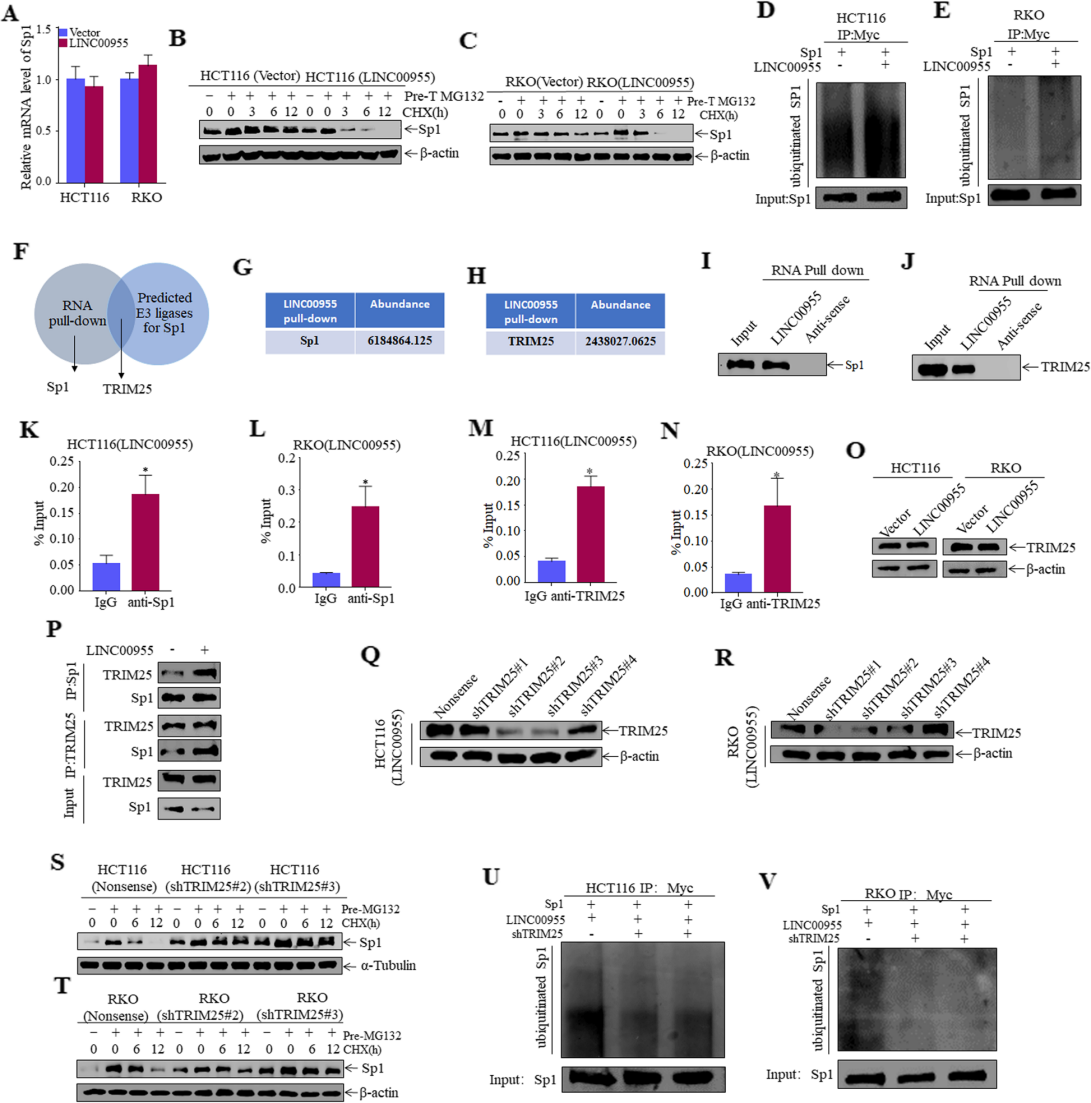
LINC00955 acts as a scaffold to promote ubiquitination and degradation of Sp1 by TRIM25. **A** Sp1 mRNA levels in HCT116 (Vector, LINC00955) and RKO (Vector, LINC00955) cells, as determined by qPCR. (B, C) Effects of MG132 and CHX on the Sp1 protein degradation rate in (**B**) HCT116 (Vector, LINC00955) and (**C**) RKO (Vector, LINC00955) cells, as determined by western blotting. **D**, **E** Effects of LINC00955 overexpression on Sp1 ubiquitination in (**D**) HCT116 and (**E**) RKO cells after the ubiquitin-IP experiment, as determined by western blotting. **F** Venn diagram screening of proteins interacting with LINC00955. **G**, **H** Mass spectrometry analysis results after RNA pull-down. **I**, **J** Interactions between (**I**) LINC00955 and Sp1, and between (**J**) LINC00955 and TRIM25, after RNA pull-down in HCT116 cells transfected with Sp1 or TRIM25, as determined by western blotting. **K**, **L** RIP assays of the interaction between LINC00955 and Sp1 in (**K**) HCT116 (LINC00955) and (**L**) RKO (LINC00955) cells. **M**, **N** RIP assays of the interaction between LINC00955 and TRIM25 in (M) HCT116 (LINC00955) and (**N**) RKO (LINC00955) cells. **O** Expression of TRIM25 in HCT116 (Vector, LINC00955) and RKO (Vector, LINC00955) cells, as determined by western blotting. **P** Co-IP assays were performed to evaluate how overexpression of LINC00955 affected the interaction between TRIM25 and Sp1. **Q**, **R** Transfection efficiency of shPHIP in (**Q**) HCT116 (LINC00955) and (**R**) RKO (LINC00955) cells, as determined by western blotting. **S**, **T** Effect of TRIM25 knockdown on Sp1 degradation in (**S**) HCT116 (LINC00955) and (**T**) RKO (LINC00955) cells, as determined by western blotting. **U**, **V** Effect of TRIM25 knockdown on Sp1 ubiquitination after the ubiquitin-IP experiment in (U) HCT116 (LINC00955) and (V) RKO (LINC00955) cells, as determined by western blotting. An asterisk (*) indicates a significant difference (*p* < 0.05)

To elucidate the molecular mechanism by which LINC00955 regulates Sp1 expression, we first evaluated the ability of LINC00955 to regulate Sp1 mRNA levels through qPCR. Sp1 mRNA levels in HCT116 (LINC00955) and RKO (LINC00955) cells, however, did not differ significantly from levels in their respective control cells (Fig. 6A). To establish whether LINC00955 regulates expression of Sp1 through ubiquitination and degradation, cells were treated with MG132 and CHX. Sp1 was degraded more rapidly in HCT116 (LINC00955) and RKO (LINC00955) cells than in their respective control cells (Fig. 6B, C), suggesting that LINC00955 was responsible for faster degradation of Sp1. In vitro ubiquitination assays revealed that the amount of ubiquitinated Sp1 protein was markedly higher in HCT116 (LINC00955) and RKO (LINC00955) cells than in their respective controls (Fig. 6D, E). To assess direct involvement of LINC00955 in regulation of Sp1 expression, biotinylated LINC00955 was used as an RNA probe in RNA pull-down assays and mass spectrometry analyses. The amount of Sp1 was much higher in biotinylated LINC00955 precipitates than in biotinylated antisense LINC00955 precipitates, with the former also being enriched in TRIM25, an E3 ubiquitin ligase that is predicted to mediate Sp1 ubiquitination and degradation (Fig. 6F–H). TRIM25 promotes ubiquitination of Sp1 at K610 in gastric cancer [20]. LncRNAs can act as scaffolds that participate in protein–protein interactions. LINC00955 may therefore serve as a scaffold to recruit E3 ligase to Sp1 and promote Sp1 ubiquitination and degradation. To test this hypothesis, RNA pull-down and RIP assays were performed. Both assays showed that LINC00955 interacts with Sp1 and TRIM25 (Fig. 6I–N). Overexpression of LINC00955, however, did not significantly alter expression of TRIM25 protein in CRC cells (Fig. 6O). The effect of LINC00955 on the binding of Sp1 to its ubiquitin E3 ligase TRIM25 was further tested by Co-IP experiments, which showed that LINC00955 overexpression increased the interaction between Sp1 and TRIM25 (Fig. 6P). To determine whether TRIM25 mediates Sp1 degradation, TRIM25 was knocked down in HCT116 (LINC00955) and RKO (LINC00955) cells (Fig. 6Q, R), and degradation rate of Sp1 was determined. TRIM25 knockdown significantly reduced the degradation of Sp1 (Fig. 6S, T), as well as its ubiquitination (Fig. 6U, V), indicating that TRIM25 is an E3 ligase that regulates Sp1 ubiquitination in CRC cells. In summary, these findings show that LINC00955 promotes interaction between Sp1 and TRIM25, thereby promoting Sp1 degradation.

### LINC00955 nucleotides 2073–2204 interact with Sp1 protein, and nucleotides 984–1135 interact with TRIM25 protein

**Fig. 7:**
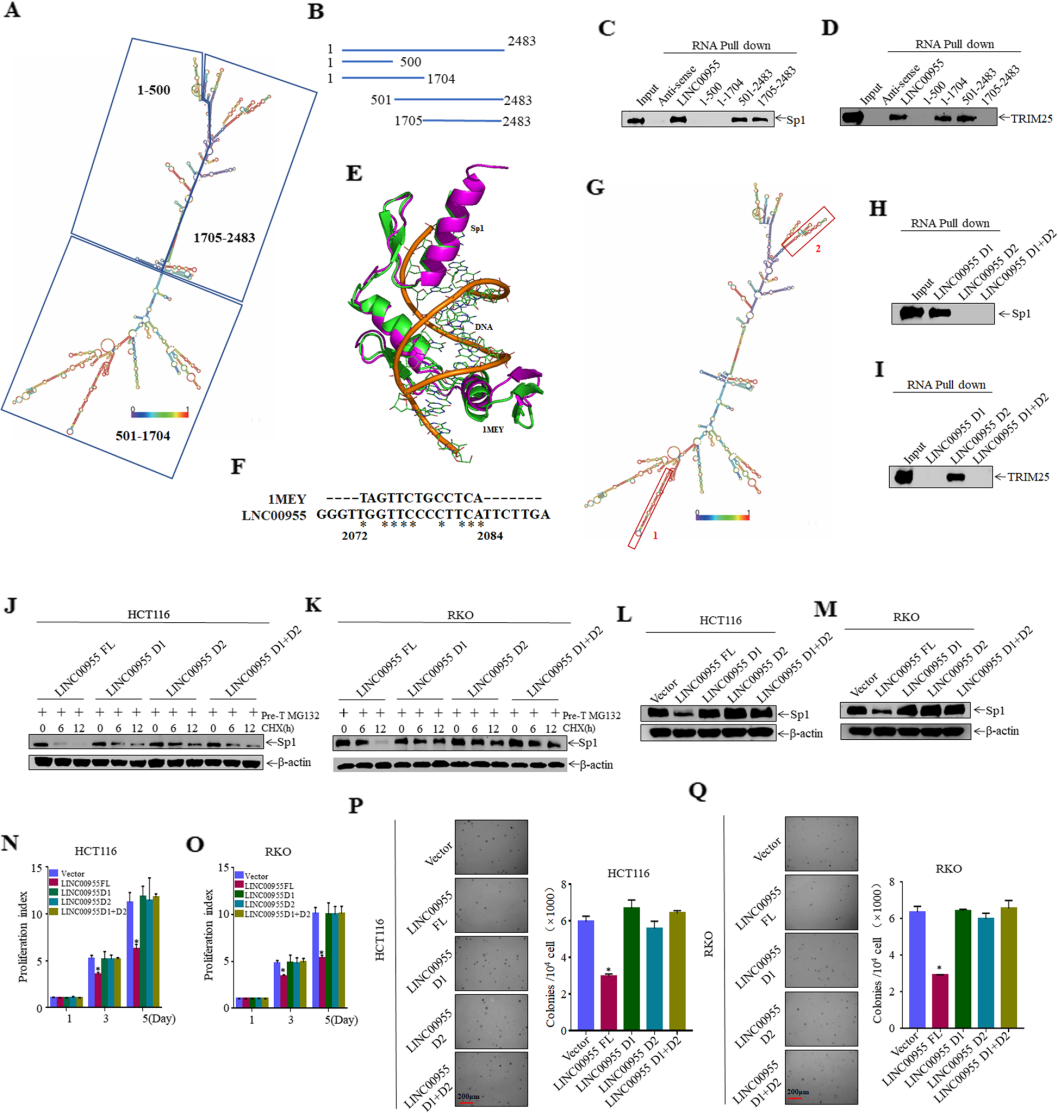
LINC00955 inhibits proliferation of CRC cells by interacting with Sp1 and TRIM25. **A** Structure of the LINC00955 fragment predicted by RNAfold. **B**–**D** The regions of LINC00955 that bind to Sp1 and TRIM25, as shown by RNA pull-down experiments using successively truncated LINC00955 fragments. **E** Structural superposition of the amino acid 626–714 domain in Sp1 (magenta) and the zinc finger protein in 1MEY (green). **F** Sequence alignment of the nucleotides in 1MEY with LINC00955. **G** Predicted LINC00955 binding regions that interact with Sp1 and TRIM25. **H**, **I** Specific regions of LINC00955 bound to Sp1 and TRIM25 proteins, as determined by RNA pull-down assays using HCT116 cells transfected with Sp1 or TRIM25. **J**, **K** Effect of the deleting the LINC00955 fragments bound to Sp1 and TRIM25 on Sp1 protein degradation in (**J**) HCT116 and (K) RKO cells, as determined by western blotting. **L**, **M** Effect of the deleting the LINC00955 fragments bound to Sp1 and TRIM25 on Sp1 protein expression in (**L**) HCT116 and (**M**) RKO cells, as determined by western blotting. **N**, **O** Effects of deleting the LINC00955 fragments bound to Sp1 and TRIM25 on the ability of (**N**) HCT116 and (**O**) RKO cells to proliferate, as measured by ATP assays. **P**, **Q** Soft agar tests were performed to investigate the effects of deleting the LINC00955 fragments bound to Sp1 and TRIM25 on the ability of (**P**) HCT116 and (**Q**) RKO cells to proliferate. An asterisk (*) indicates a significant difference (*p* < 0.05)

To determine the sites within LINC00955 that interact with Sp1 and TRIM25, the structure of LINC00955 was predicted by RNAfold (Fig. 7A). Because proteins tend to interact with the stem-loop structures of RNAs, biotinylated RNA probes corresponding to LINC00955 nucleotides 1–500, 1–1704, 501–2483, and 1705–2483 were synthesized for RNA pull-down experiments (Fig. 7B). LINC00955 mutants containing nucleotides 1705–2483 retained the ability to bind to Sp1 (Fig. 7C), whereas LINC00955 mutants containing nucleotides 501–1704 retained the ability to bind to TRIM25 (Fig. 7D). To identify the regions of Sp1 and TRIM25 that bind to LINC00955, the Protein Data Bank was searched using the Dali web server to identify folds similar to the structural domains in Sp1 (i.e., amino acids 429–558, and 626–714) and TRIM25 (i.e., amino acids 1–84, 105–190, and 431–630). The domain corresponding to amino acids 626–714 in Sp1 contained a structural fold similar to that of a designed zinc finger protein bound to DNA (pdb ID: 1MEY) (Fig. 7E). Sequence alignment showed that the nucleotides in 1MEY aligned well with nucleotides 2072–2084 of LINC00955 (Fig. 7F). Nucleotides 2072–2084 are in a double-stranded helical region (i.e., nucleotides 2073–2204, named ‘2’) (Fig. 7G). The high probability of base pairs in the helical region and the well-aligned sequences suggest that the domain corresponding to amino acids 626–714 in Sp1 may bind to nucleotides 2073–2204 in LINC00955. Evaluation of the three domains in the TRIM25 protein (i.e., amino acids 1–84, 105–190, and 431–630) failed to identify any similar structures that bound directly to nucleotides. However, TRIM25 prefers to bind GC-rich sequences [21]. Nucleotides 984–1135 of LINC00955 had a significantly higher GC-content (74/132) (Fig. 7G) than other regions of LINC00955 (i.e., nucleotides 984–1135, named ‘1’), suggesting that nucleotides 984–1135 in LINC00955 might bind to TRIM25. To test whether the region of LINC00955 corresponding to nucleotides 984–1135 binds to TRIM25, and whether LINC00955 nucleotides 2073–2204 bind to amino acids 626–714 of Sp1 (Fig. 7G), biotinylated LINC00955 probes that deleted nucleotides 984–1135 and/or 2073–2204 were synthesized and utilized in RNA pull-down experiments. Deletion of the LINC00955 region corresponding to nucleotides 2073–2204 abolished interactions with Sp1 protein (Figs. 7H), whereas deletion of nucleotides 984–1135 abolished interactions with TRIM25 protein (Fig. 7I). Deletion of both regions at the same time resulted in a failure to bind both Sp1 and TRIM25 (Fig. 7H, I). These findings indicate that the LINC00955 regions corresponding to nucleotides 2073–2204 and 984–1135 bind to Sp1 and TRIM25, respectively.

To further determine whether the interactions of LINC00955 with Sp1 and TRIM25 proteins inhibit the proliferation of CRC cells, plasmids overexpressing LINC00955 lacking nucleotides 984–1135 and/or 2073–2204 were synthesized and used to construct stable transfected cell lines (Figure S2A, B). Only full-length LINC00955 accelerated Sp1 degradation and downregulated Sp1 expression, whereas plasmids expressing LINC00955 lacking nucleotides 984–1135 and/or 2073–2204 did not (Fig. 7J–M). ATP and soft agar experiments showed that LINC00955 fragments lacking the Sp1-binding domain, the TRIM25-binding domain, and both regions did not inhibit CRC cell proliferation (Fig. 7N–Q), which supported the conclusion that the function of LINC00955 depends on its binding to Sp1 and TRIM25.

### Correlations among Sp1, DNMT3B, PHIP, and CDK2 protein levels in clinical CRC tissues

**Fig. 8:**
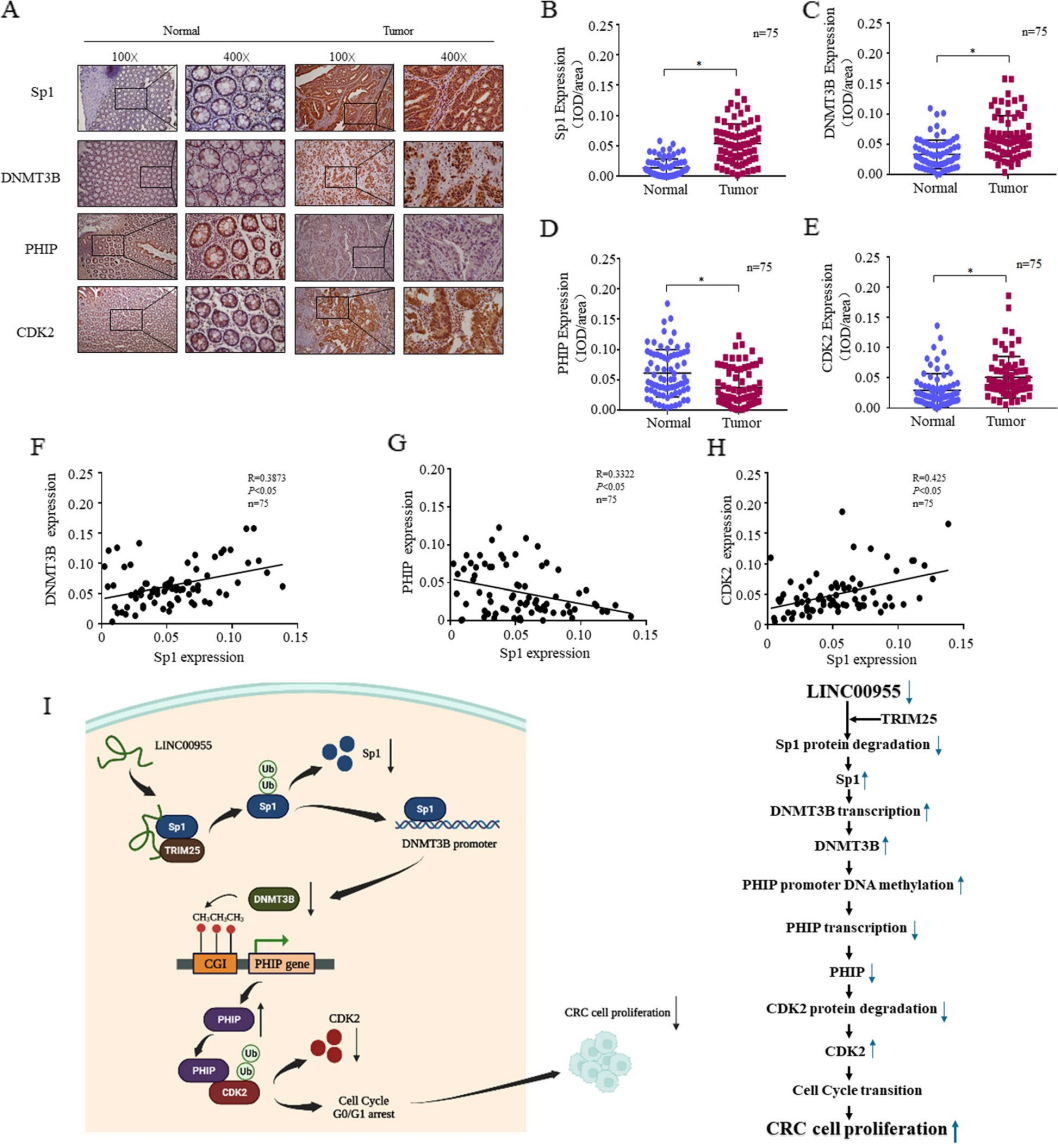
Correlations between expression of Sp1, DNMT3B, PHIP, and CDK2 proteins. **A**–**E** Levels of Sp1, DNMT3B, PHIP, and CDK2 protein in CRC tissues and adjacent normal tissues, as determined by immunohistochemical analysis of 75 pairs of clinical samples. **F**, **H** Correlations between expression of the above proteins in 75 pairs of CRC tissues and normal tissue samples. **I** Mechanistic diagram of inhibition of CRC cell proliferation by LINC00955 through the TRIM25/Sp1/DNMT3B/PHIP/CDK2 axis

To verify the correlations between LINC00955 and its downstream genes in vivo, we performed immunohistochemistry (IHC) to detect expression of Sp1, DNMT3B, PHIP, and CDK2 in 75 pairs of CRC and normal tissues. The protein levels of Sp1, DNMT3B, and CDK2 were significantly higher in CRC tissues than in normal tissues, while the protein levels of PHIP were significantly lower in CRC tissues than in normal tissues (Fig. 8A–E), which correlated with the downregulation of LINC00955 in CRC. Further correlation analysis showed that Sp1 protein levels were positively correlated with DNMT3B and CDK2 protein levels, but negatively correlated with PHIP protein levels (Fig. 8F–H), which is consistent with the results of in vitro studies. These results indicate that the LINC00955-Sp1-DNMT3B-PHIP-CDK2 regulatory pathway has clinical significance in regulation of CRC progression. Finally, the molecular mechanism of LINC00955 inhibiting the malignant proliferation of CRC cells is mapped (Fig. 8I).

## Discussion

LncRNAs have significant roles in the pathophysiology and development of a number of malignancies [22], indicating that combined targeting of dysregulated lncRNAs may become a potential strategy for cancer treatment in the future [23]. To date, however, only the roles of a few lncRNAs have been thoroughly characterized. The discovery and identification of additional lncRNAs, and their underlying mechanisms, are of great significance for cancer treatment. The present study, using both bioinformatics and experimental analyses, demonstrated that LINC00955 expression was substantially downregulated in CRC tissues. Moreover, lower expression of LINC00955 was linked to a poorer prognosis in patients with CRC, suggesting that LINC00955 may be a prognostic biomarker. LINC00955 inhibited CRC cell proliferation in vitro and in vivo. When considered together, these results demonstrate that LINC00955 may be a significant tumor suppressor in CRC.

The cell cycle is both important and strictly controlled, as abnormal cell cycle processes can lead to genome instability and cancer progression [24]. Little is known about the molecular function of lncRNAs in cell cycle regulation in comparison with the diverse proteins involved in cell cycle regulation and linked with cancer-causing mutations [25]. Several lncRNAs regulate the cell cycle and cell proliferation by directly regulating DNA replication or indirectly controlling expression of vital cell cycle-regulating genes [26]. For example, lncRNA MIR31HG binds to HIF1A, targets p21, and promotes cell proliferation by enhancing cell cycle progression in HNSCC [27]. The results presented herein showed that LINC00955 blocked the G0/G1 phase of the cell cycle and inhibited proliferation of CRC cells by reducing expression of CDK2. CDK2 is activated by complexing with a cyclin, and is active from G1 phase progression and throughout S phase [28]. CDK2 expression is upregulated in cancers [29], and CDK2 is crucial for anchorage-independent proliferation mediated by oncogenes [30]. Cancer therapy is thought to target CDK2 in some cases, and several small-molecule CDK2 inhibitors are currently undergoing clinical trials. One example is Alvocidib, a purine analogue and combination inhibitor [31]; however, this agent shows unsatisfactory efficacy, high toxicity, and non-specificity. Additional key regulators of CDK2 may suppress cell proliferation during oncogenesis. For example, the present study identified a new lncRNA that regulates CDK2 in CRC, suggesting a new direction for CDK2 inhibitors and suppressors of CRC cell proliferation.

Current research on CDK2 is mainly concerned with its kinase activity [32]. Fewer studies, however, have evaluated the regulation of CDK2 expression, especially its stability. The current investigation discovered that LINC00955 mediates the ubiquitination-degradation of CDK2 through the E3 ligase PHIP. Ubiquitination is a significant post-translational change involved in controlling a number of biological functions. The ubiquitination cascade includes activating enzymes (E1s), conjugating enzymes (E2s), and ligases (E3s) [33], with E3 ligase playing a crucial role in specifically determining the ubiquitination and degradation of target proteins. PHIP is a cytosolic protein encoded on chromosome 6q14.1, which participates in insulin and IGF-1 signaling and interacts only with the PH domain of IRS-1 [34]. To our knowledge, PHIP has not been reported to function as a substrate protein for E3 ligase. The present study is therefore the first to show that CDK2 is an ubiquitinated substrate of PHIP. Evaluation of clinical tissue samples demonstrated that PHIP expression was lower in CRC tissues than in normal colon tissues. Moreover, functional experiments showed that PHIP plays a role as a tumor suppressor gene during development of CRC.

Additionally, the current study discovered that LINC00955 markedly increased PHIP promoter activity. PHIP inhibited CRC tumor cell proliferation. Several tumor suppressor genes can be rendered inactive by aberrant CpG island methylation in their promoter regions, highlighting the significance of epigenetic changes during carcinogenesis [35]. DNA methylation patterns are generally regulated by DNA methyltransferases (DNMTs) [36], with DNMT3B acting as a de novo methyltransferase [37]. In HCT116 colon cancer cells, disruption of DNMT1 and DNMT3B decreases the 5-mC concentration by 95% and delays cell proliferation [38]. Few studies to date, however, have focused on the specific molecular mechanisms by which DNMT3B participates in the process of CRC proliferation. The present study proposes a novel mechanism by which LINC00955 inhibits CRC cell proliferation by downregulating DNMT3B to inhibit methylation of the PHIP promoter. The PHIP gene, however, is not likely to be the only target of DNMT3B during cell proliferation. Alterations in expression of intracellular DNA methyltransferases must therefore affect other candidate genes and related pathways, indicating a need for additional studies. Assessment of the mechanism by which LINC00955 regulates DNMT3B found that LINC00955 inhibits DNMT3B transcription by downregulating transcription factor Sp1.

The molecular processes by which lncRNAs function in the growth of tumors are intricate. LncRNAs typically exert their biological functions through physical interactions with regulatory proteins, miRNAs, or other cellular factors [39], although evidence suggests that it may be more important for lncRNAs to exert their biological functions through their target proteins. Some lncRNAs remain connected to their transcription sites, and interact with proteins to regulate expression of cis genes [40]. Some of them serve as molecular spies and bind to particular transcription factors, preventing them from attaching to DNA [41]. LncRNAs can also participate in protein–protein interactions. The transcriptional regulator Sp1 belongs to the family of transcription factors [42]. Sp1 was identified as a promoter-specific binding factor involved in a number of biological processes in mammalian cells [43]. Sp1 plays an important role in CRC by regulating genes involved in all cancer-related processes, including growth factor-independent proliferation, immortality, evasion of apoptosis, angiogenesis, tissue invasion, and metastasis [44, 45]. The transcriptional activity, DNA-binding affinity, and protein stability of Sp1 can all be changed post-translationally [46]. Sp1 is frequently post-translationally modified through phosphorylation, glycosylation, acetylation, ubiquitination, and sumoylation [47], with ubiquitination being an important post-translational modification [48]. Several E3 ligases specifically recognize Sp1 and mediate its ubiquitination and subsequent degradation [49, 50]. Less is known, however, about the biological roles of lncRNAs during E3 ligase-mediated ubiquitination and degradation of Sp1.

The present study found that LINC00955 post-translationally regulates Sp1 ubiquitination and degradation by promoting the binding of E3 ligase to Sp1. The TRIM family of proteins, which is distinguished by the presence of three conserved N-terminal domains, a RING domain, one or two B-Boxes (B1/B2), and a coiled-coil domain, includes the 17 beta-estradiol and type I IFN-inducible E3 ligase known as TRIM25 [51]. TRIM25 acts as an E3 ubiquitin ligase that promotes ubiquitination of Sp1 at K610 [20]. The present study found that LINC00955 promotes degradation of Sp1 by enhancing binding of the E3 ligase TRIM25 to Sp1, with subsequent degradation of Sp1 protein. LINC00955 nucleotides 2073–2204 interact with Sp1 protein, and nucleotides 984–1135 interact with TRIM25 protein. LINC00955 serves as a scaffold for protein–protein interactions that inhibit proliferation of CRC, indicating that LINC00955 plays a direct role in proliferation of CRC. In recent years, intracellular protein degradation pathways and the development of protein-targeted degradation technology have become of interest to researchers in the field of drug research and development [52]. The current research found that LINC00955 can act as a scaffold molecule that participates in the ubiquitination and degradation process, providing new ideas and directions for research on ubiquitin–proteasome systems.

## Conclusions

In conclusion, LINC00955 is downregulated in CRC and inhibits CRC cell proliferation by acting as a molecular scaffold of TRIM25 and Sp1 to inhibit the DNMT3B/PHIP/CDK2 axis.

### Supplementary Information

**Additional file 1.** 

**Additional file 2:** **Figure S1.** (A) Construction of A truncated PHIP promoter-driven luciferase reporter. (B, C) The *PHIP-1* promoter-driven luciferase reporters and the *PHIP-2* promoter-driven luciferase reporters were transferred into HCT116 (Vector, LINC00955) and RKO (Vector, LINC00955) cells, respectively, and their promoter activity was measured. **Figure S2. **(A, B) Stable transfection efficiency after deletion the LINC00955 fragment bound to Sp1 and TRIM25 in HCT116 and RKO cells, as determined by qPCR.

**Additional file 3:** **Table S1.** Information about CRC patients including case number, gender, age and tumor largest diameter.

## Data Availability

The data that support the findings of this study are openly available in ProteomeXchange Dataset at (https://www.ebi.ac.uk/pride/archive/). Proteomics data accession number is PXD041773, and mass spectrometry data accession number is PXD041514.

## Abbreviations

CRC: Colorectal cancer
lncRNAs: Long non-coding RNAs
LINC00955: Long Intergenic Non-protein Coding RNA 955
TRIM25: Tripartite motif containing 25
Sp1: Sp1 transcription factor
DNMT3B: DNA methyltransferase 3 beta
PHIP: Pleckstrin homology domain interacting protein
CDK2: Cyclin dependent kinase 2
HIF1α: Hypoxia inducible-factor 1α
CHX: Cycloheximide
