# Supplementary material for: LINC00955 suppresses colorectal cancer growth by acting as a molecular scaffold of TRIM25 and Sp1 to Inhibit DNMT3B-mediated methylation of the PHIP promoter
Source: BMC Cancer. 2023 Sep 23;23:898. doi: 10.1186/s12885-023-11403-2 (PMC10518100; doi:10.1186/s12885-023-11403-2)
Supplement: Supplementary file 1 — Additional file 1. [file 12885_2023_11403_MOESM1_ESM.pdf]

Figuer2 C

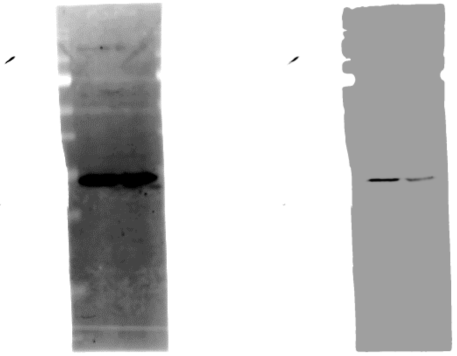

M3-HCT116-CDK2

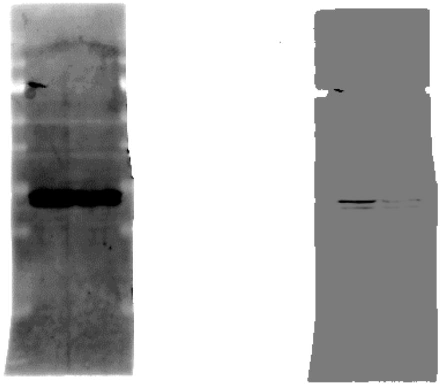

M2-RKO-CDK2

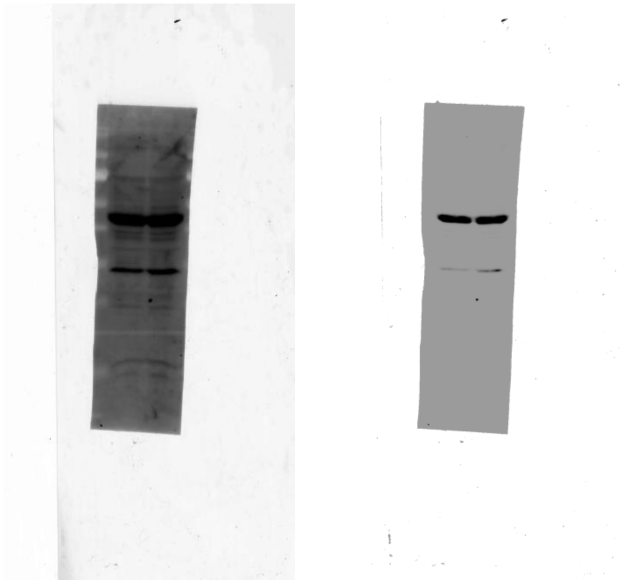

M3-HCT116-CYCLINE2

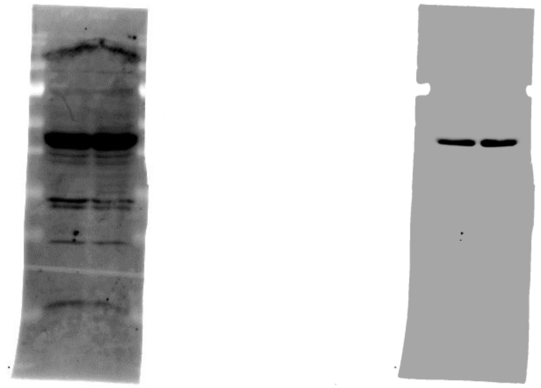

M2-RKO-CYCLINE2

Figuer2 C

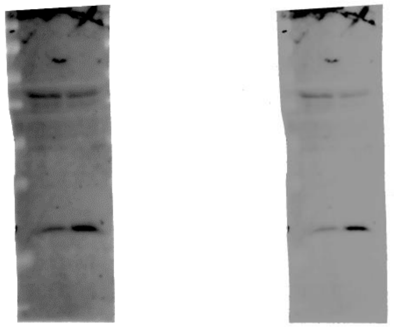

M3-HCT116-P21

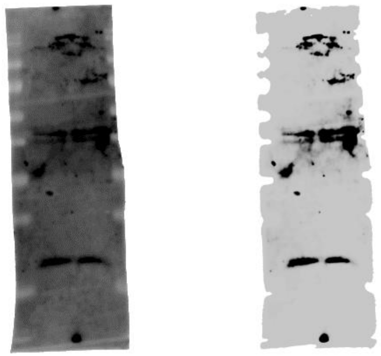

M2-RKO-P21

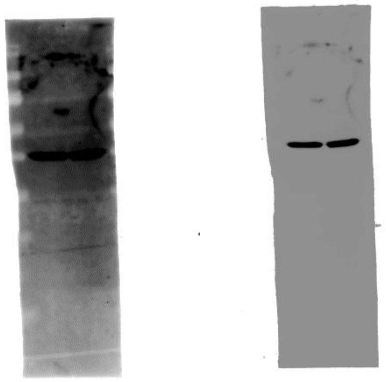

M3-HCT116-β-ACTION

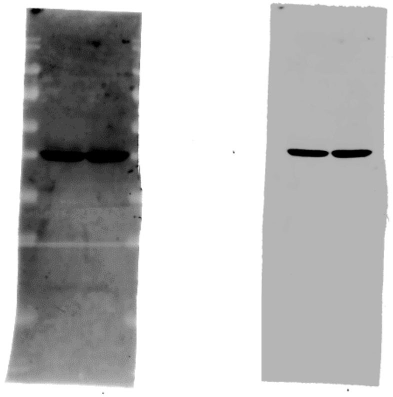

M2-RKO-β-ACTION

Figuer2 C

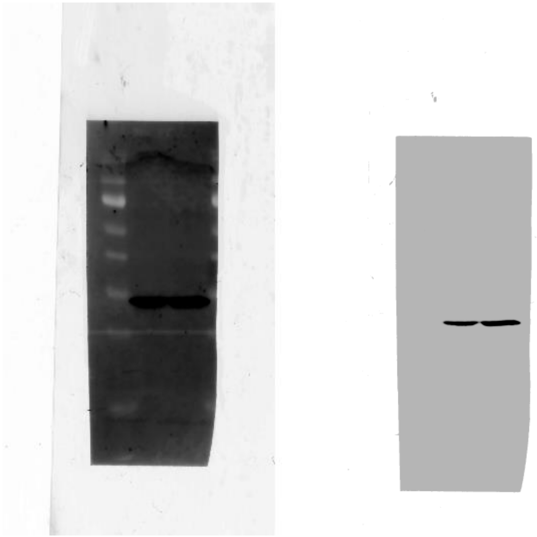

M1-HCT116-CDK4

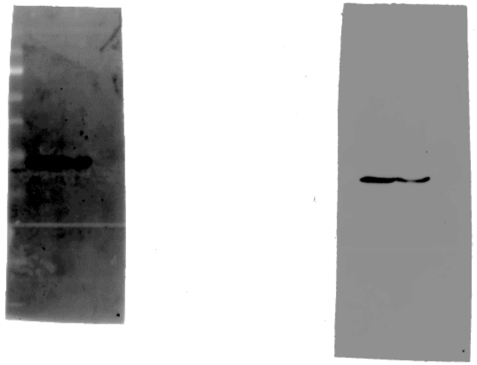

M4-RKO-CDK4

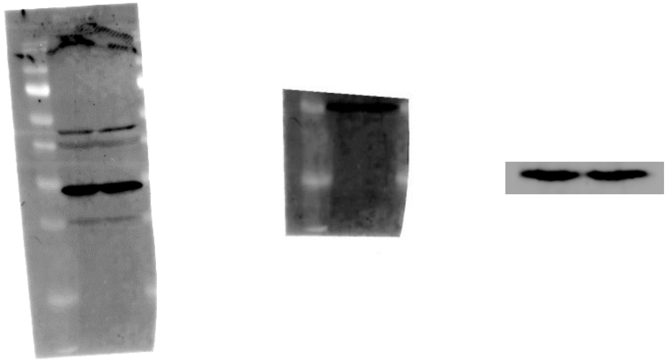

M1-HCT116-P27

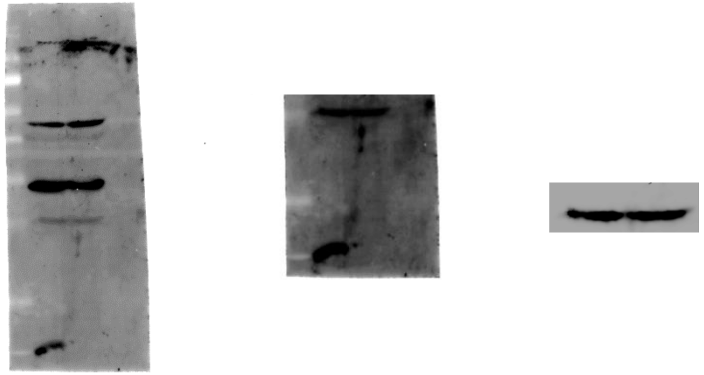

M4-RKO-P27

Figuer2 C

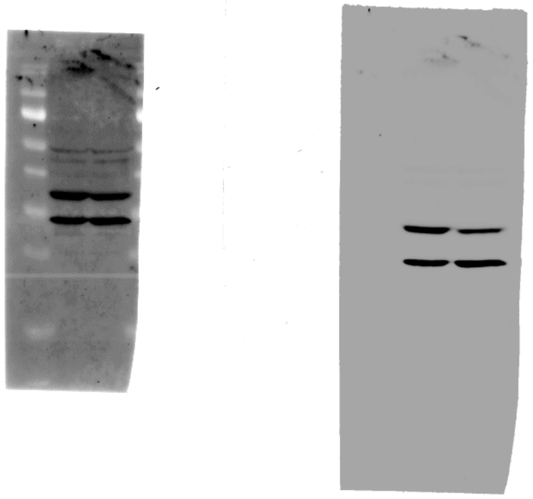

M1-HCT116-CDK6

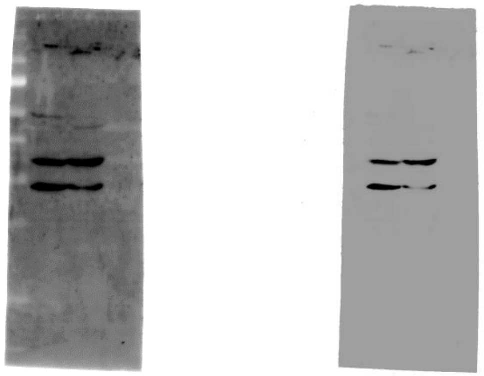

M4-RKO-CDK6

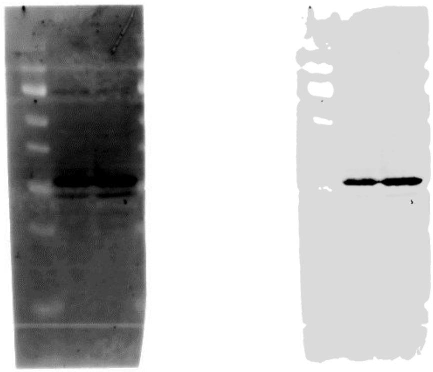

M1-HCT116-CYCLIND1

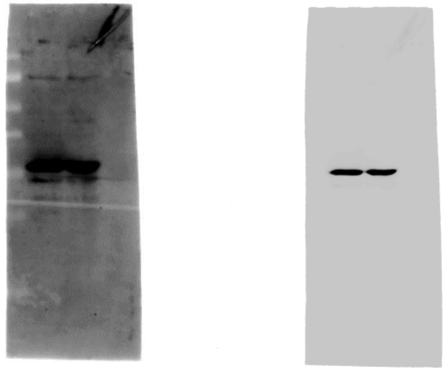

M4-RKO-CYCLIND1

Figuer2 C

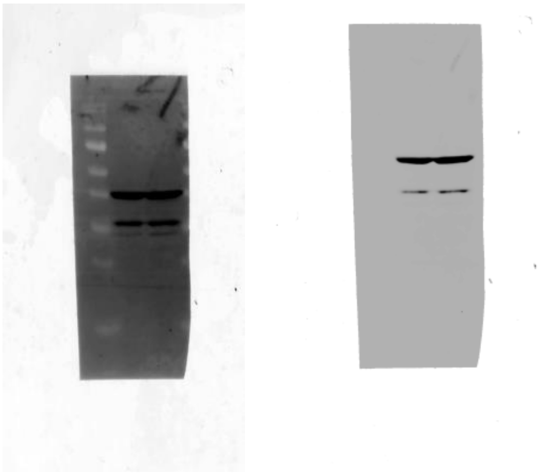

M1-HCT116-β-ACTION

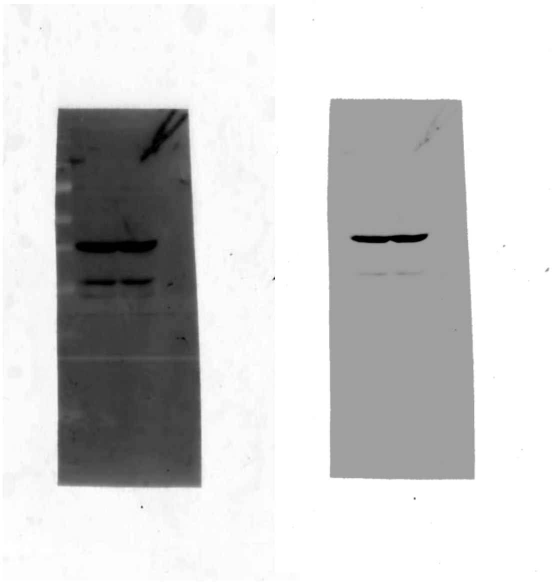

M4-RKO-β-ACTION

Figuer2 D

HCT116(linc00955)-vector/CDK2

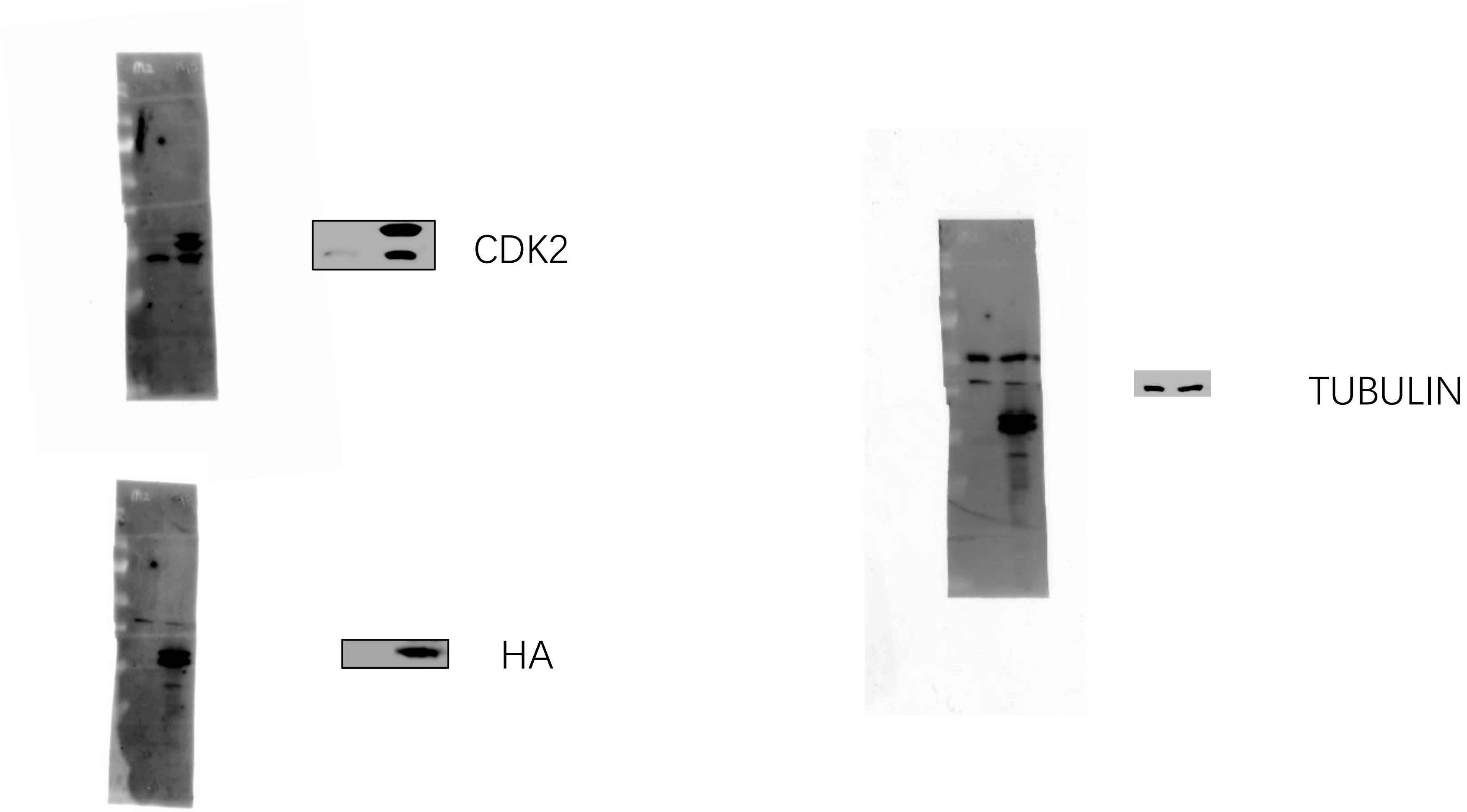

Figuer2 E

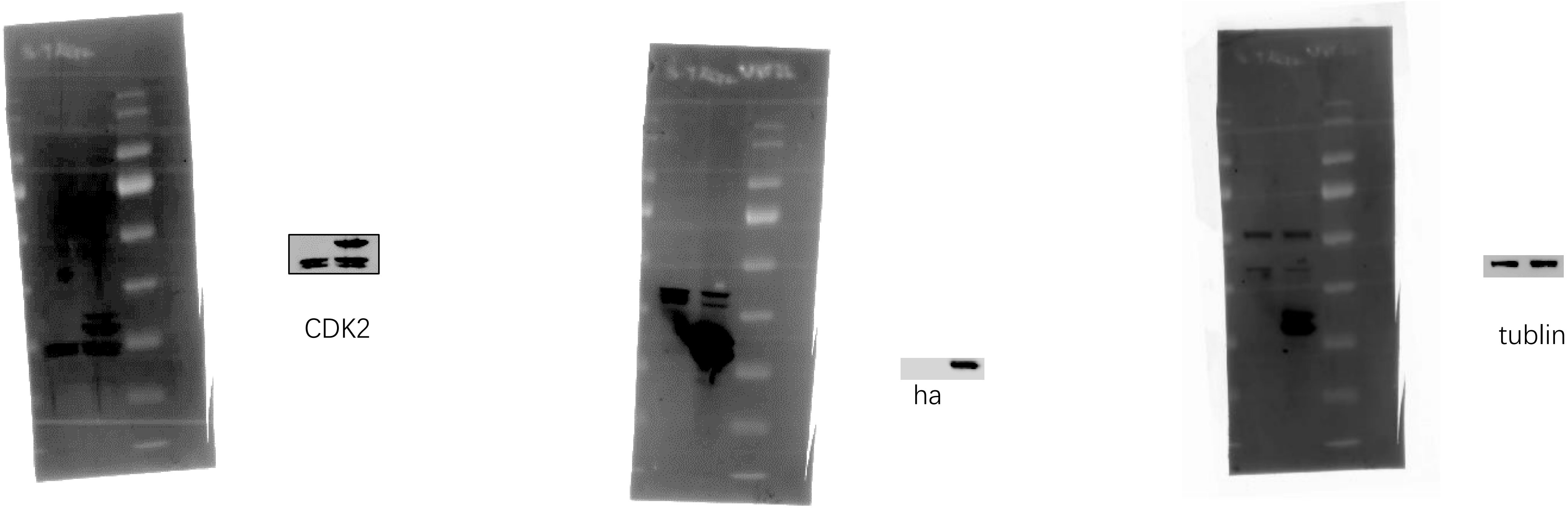

Figuer3 B

HCT116细胞CDK2降解速率

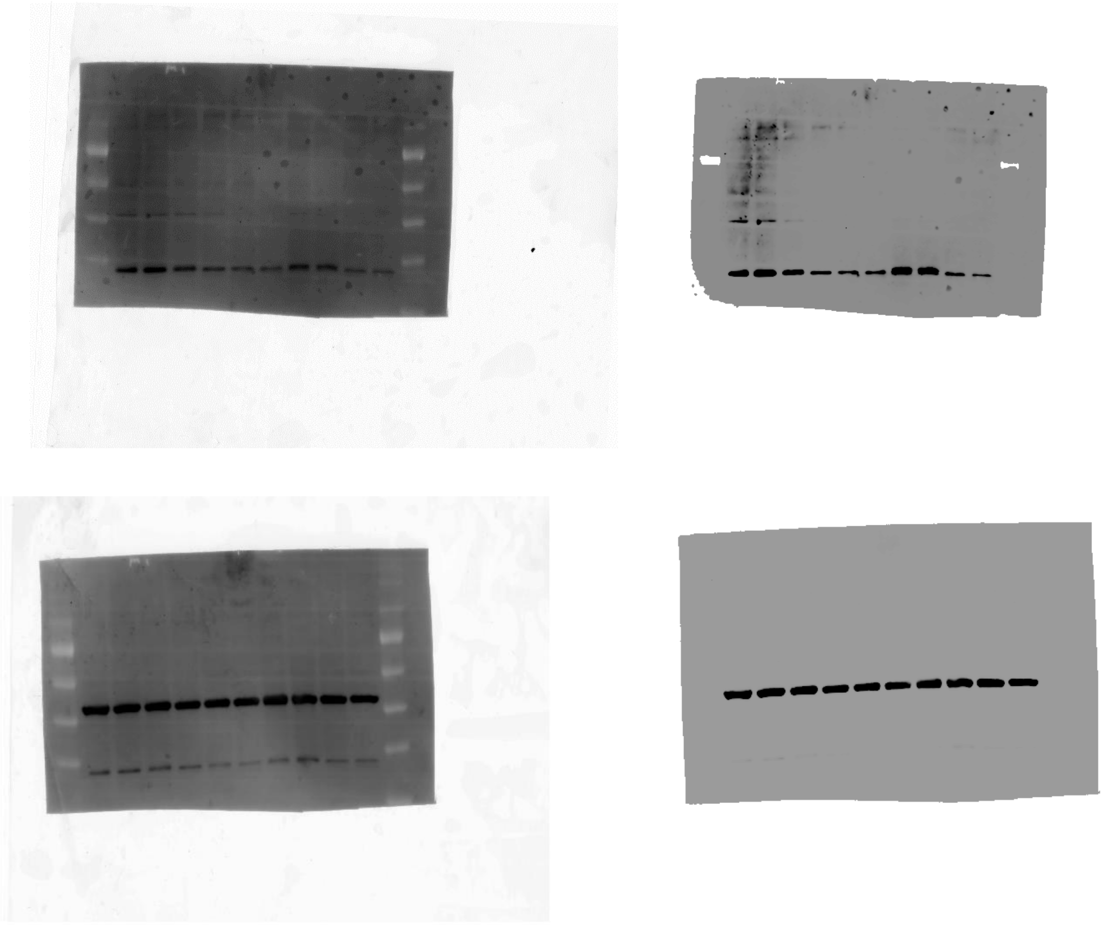

CDK2

actin

Figuer3 C

RKO细胞CDK2降解

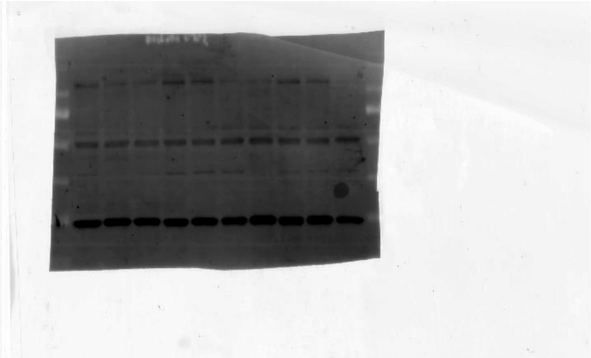

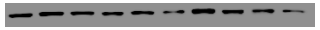

CDK2

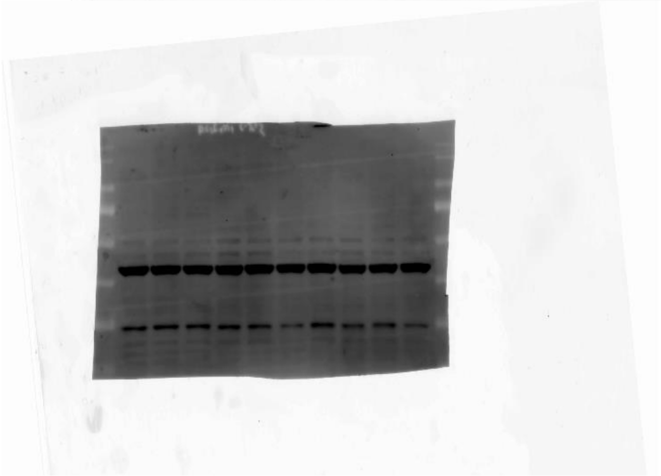

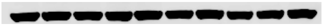

ACTIN

PHIP Figuer3 E

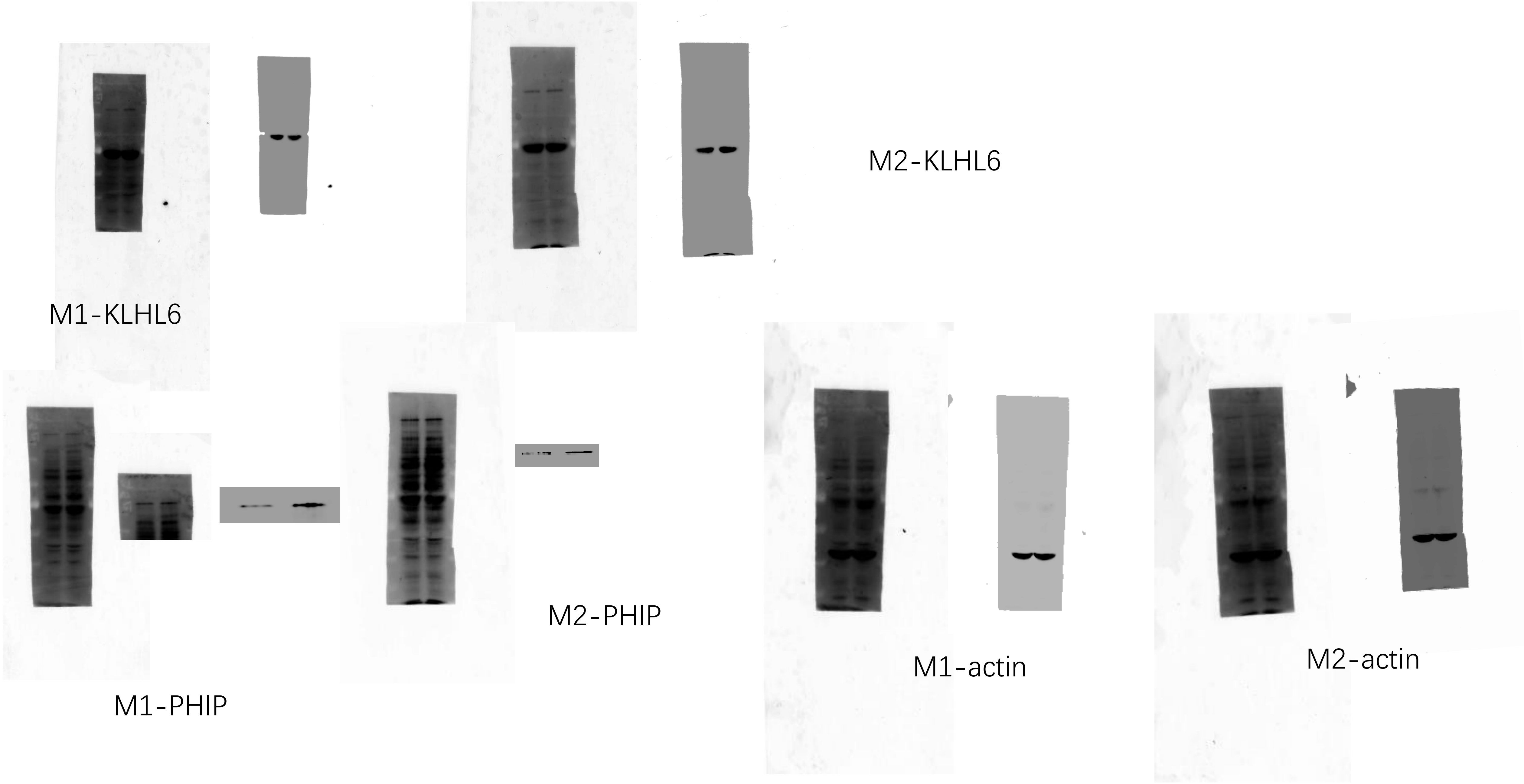

Figuer3 F

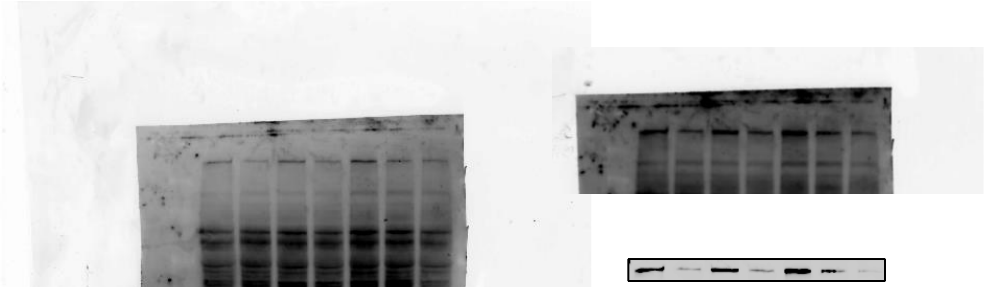

PHIP

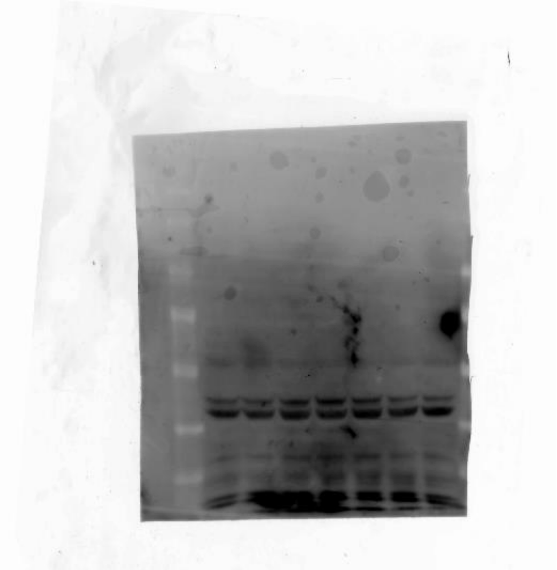

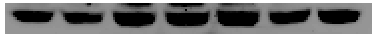

actin

Figuer3 G

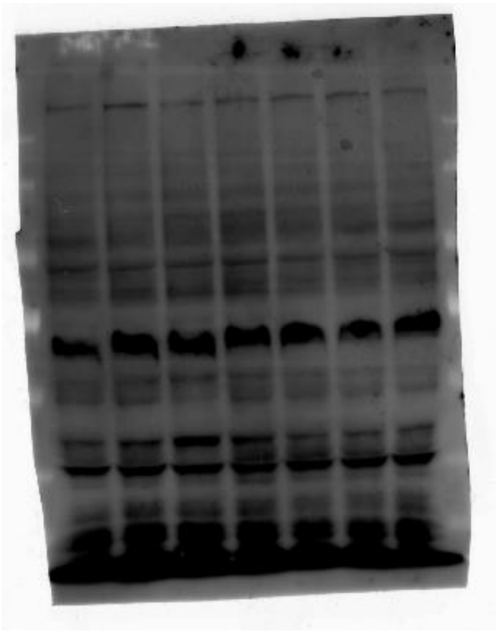

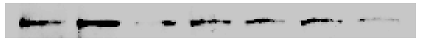

PHIP

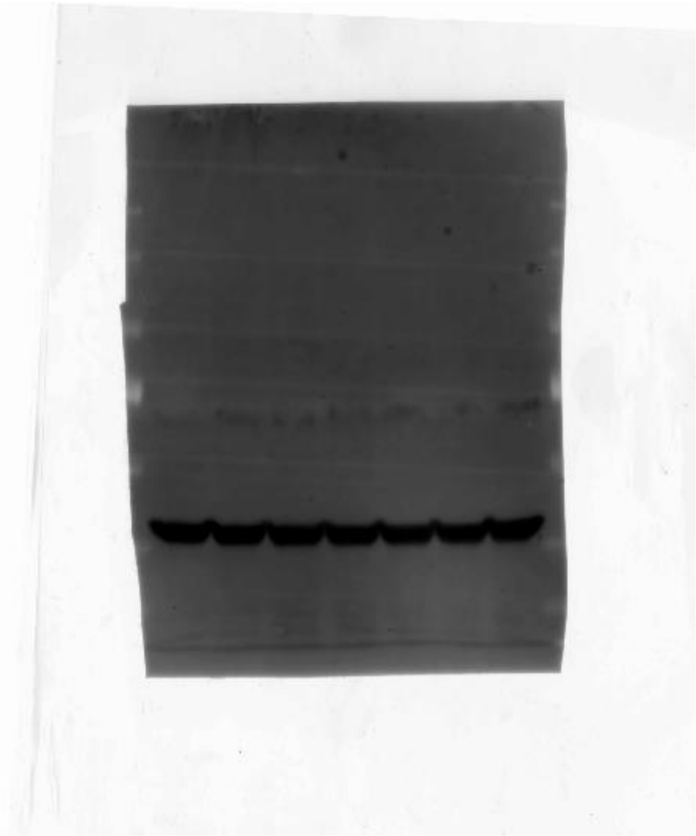

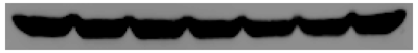

actin

Figuer3 H

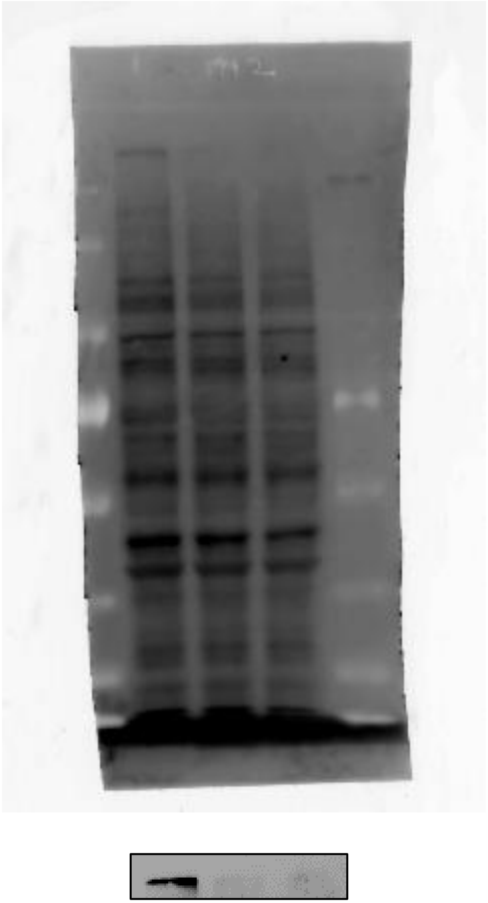

PHIP

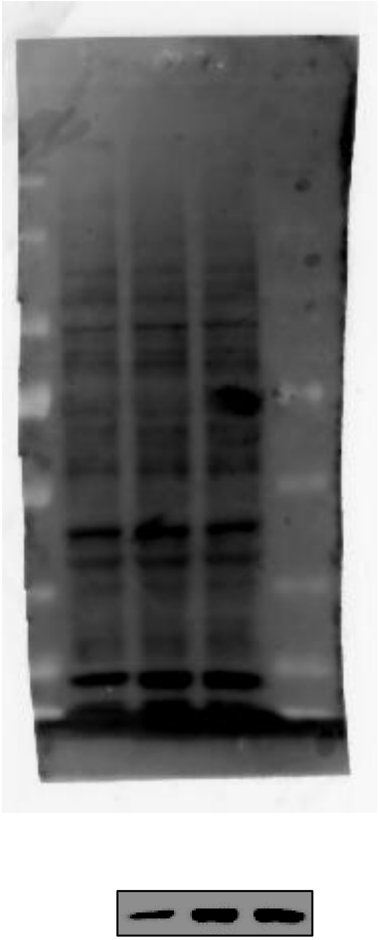

CDK2

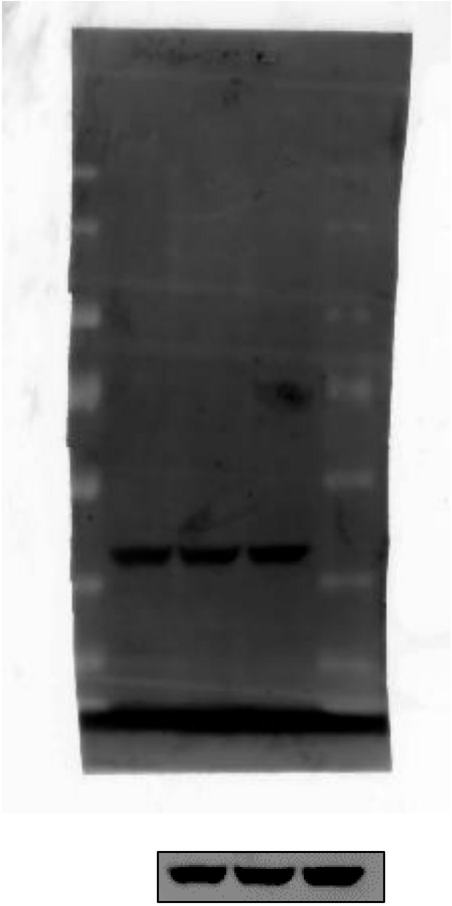

ACTIN

Figuer3 I

2021.9.6扫

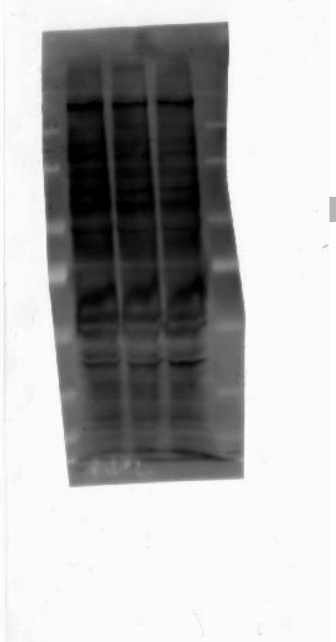

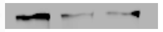

PHIP

2021.9.7扫

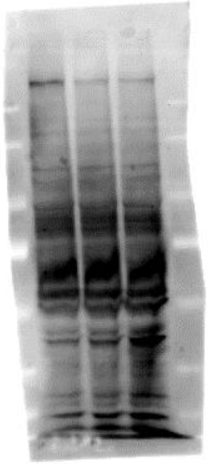

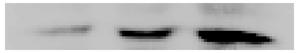

CDK2

2021.9.8扫

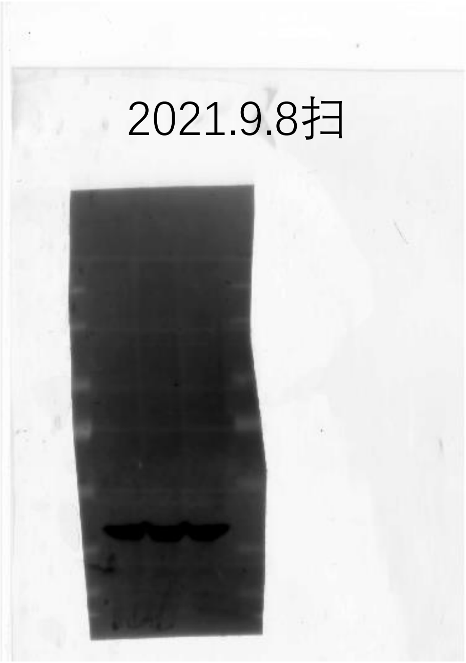

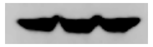

ACTIN

Figuer3 J

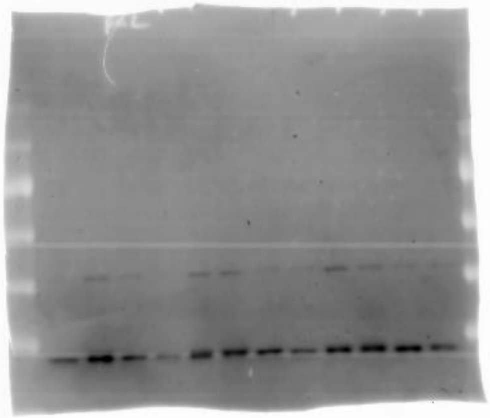

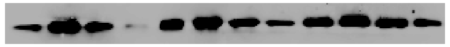

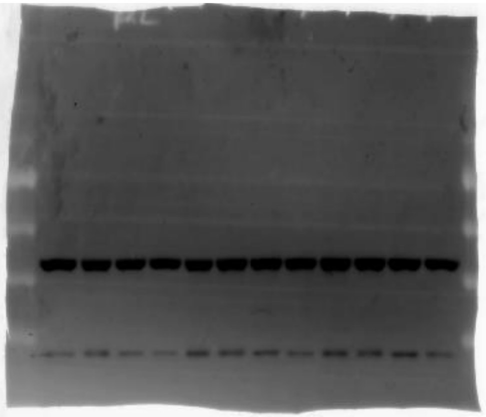

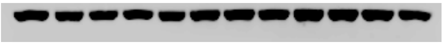

Figuer3 K

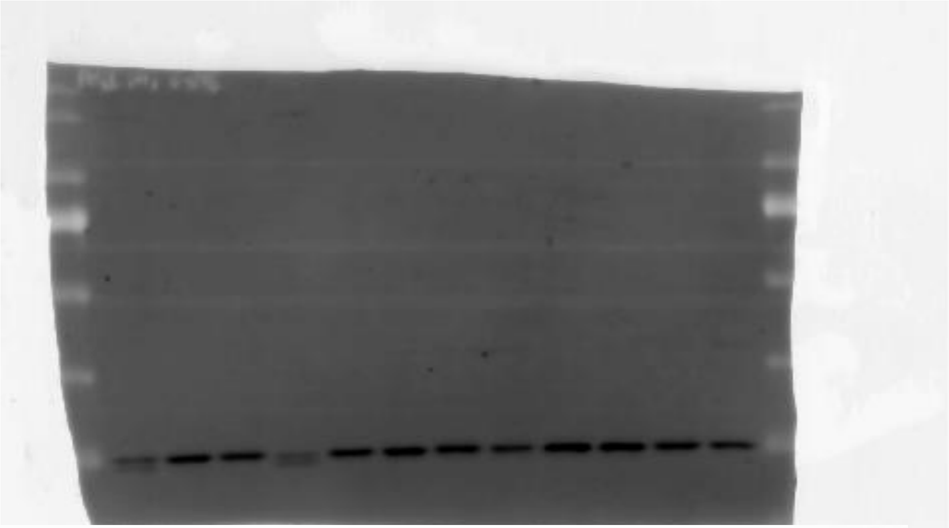

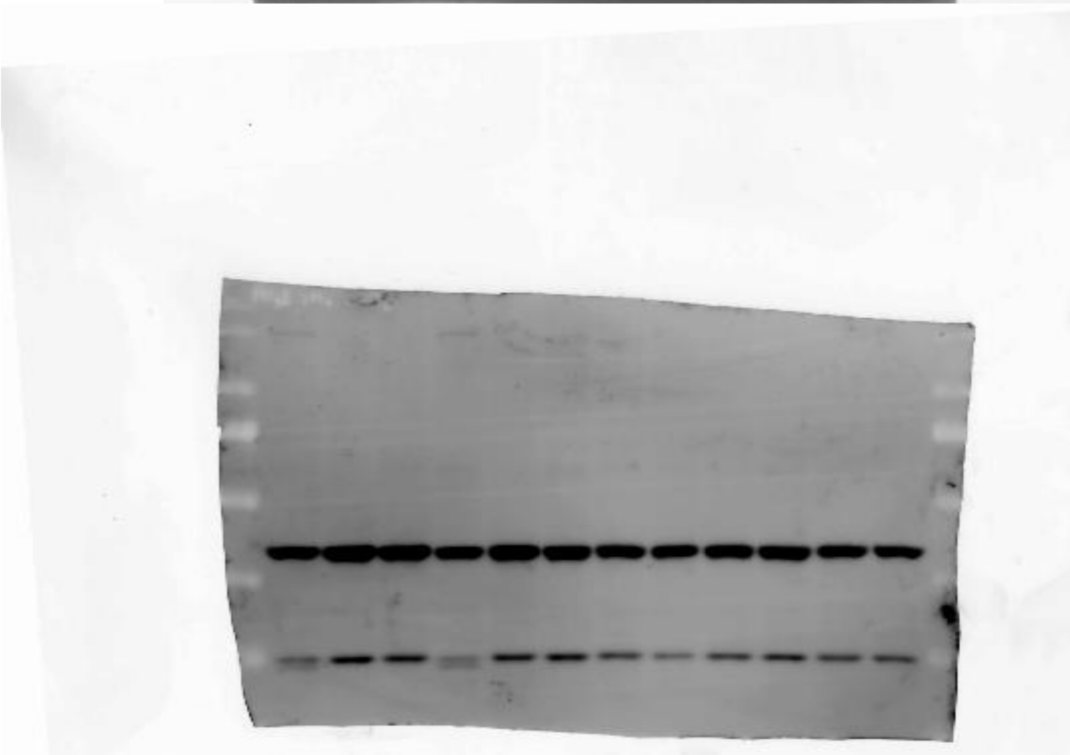

CDK2

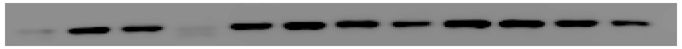

ACTIN

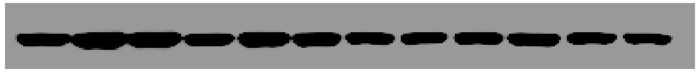

Figuer3 L

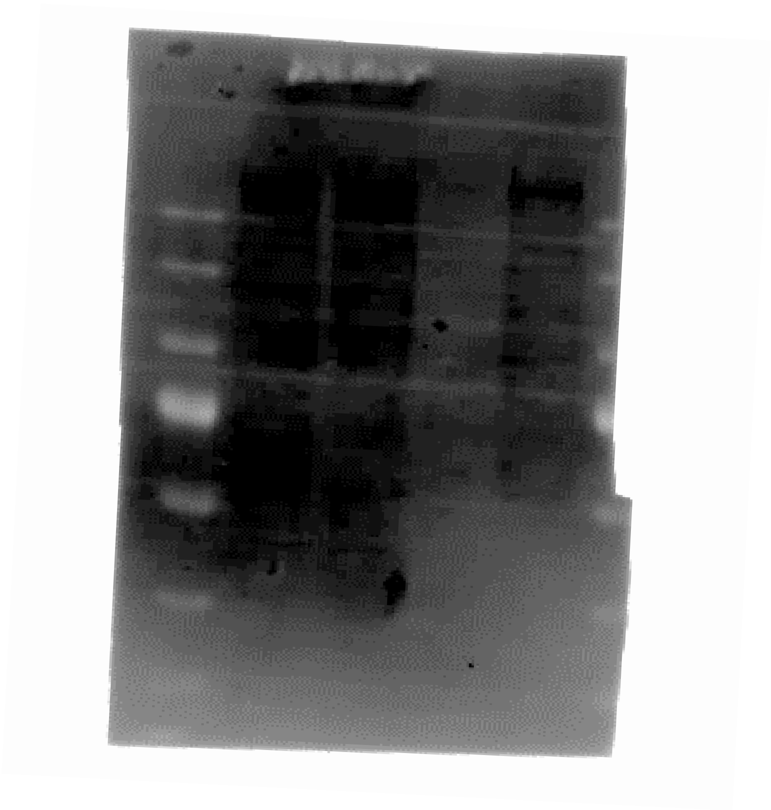

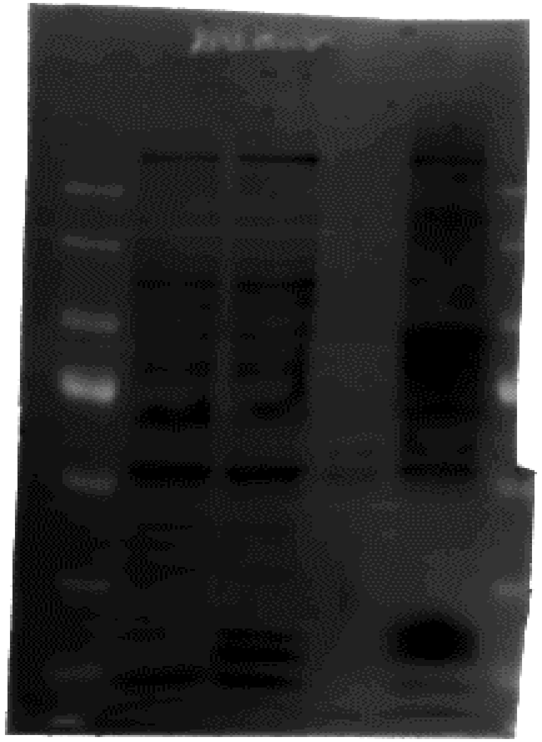

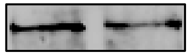

PHIP

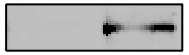

PHIP

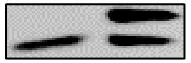

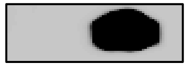

Figuer3 M

HCT116

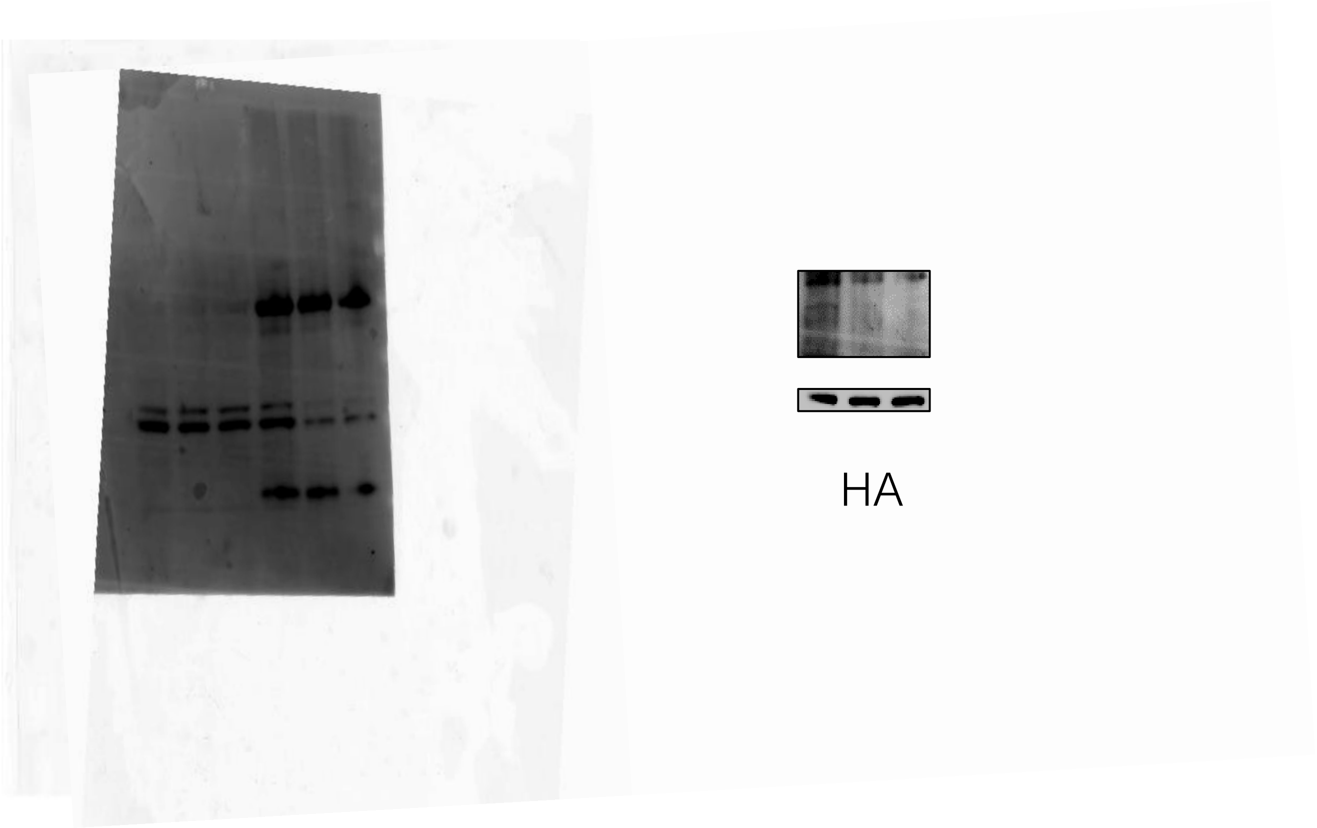

HA

Figuer3 N

RKO

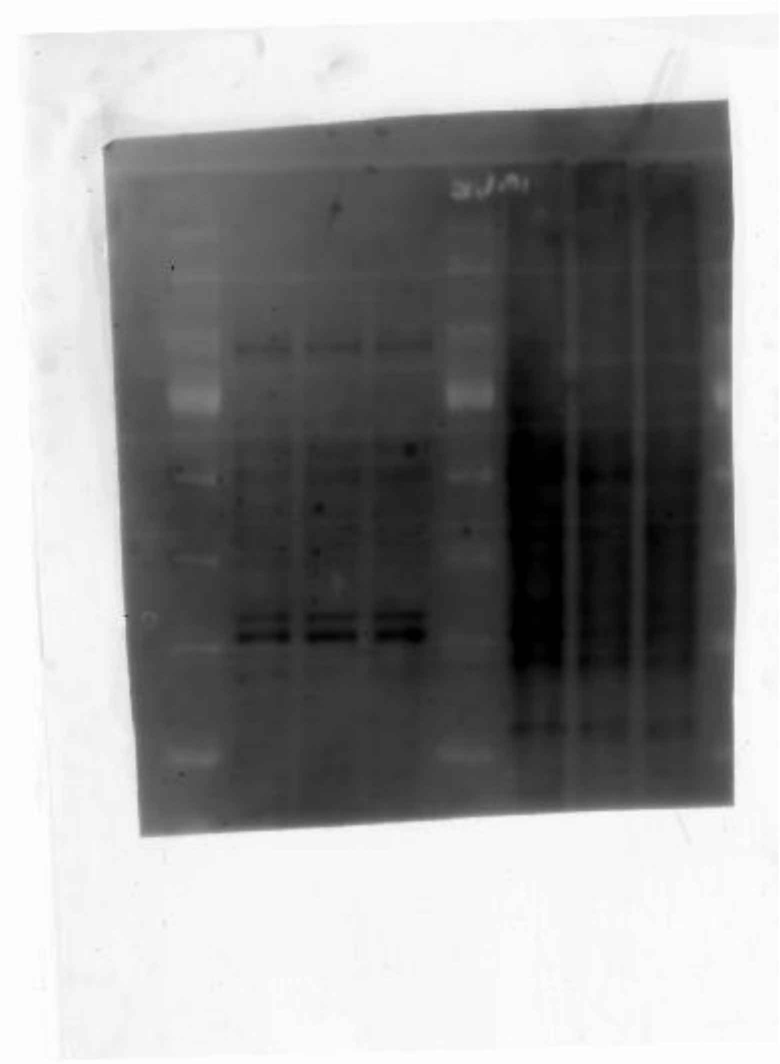

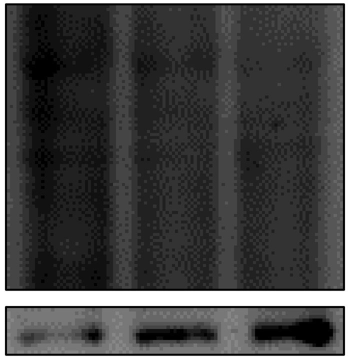

Figuer4 D

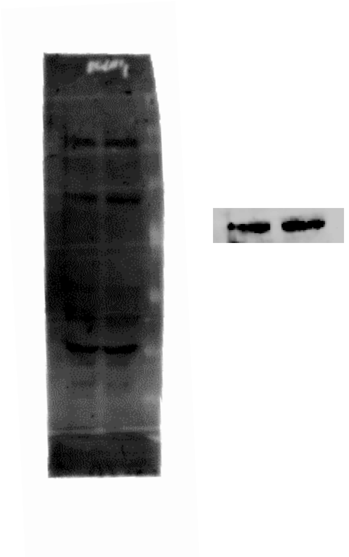

M1-SP3

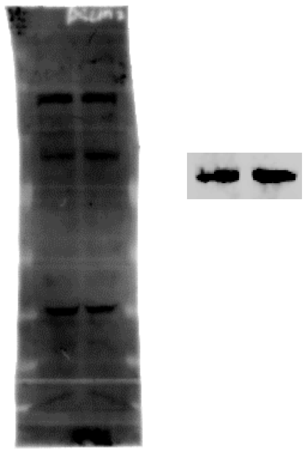

M2-SP3

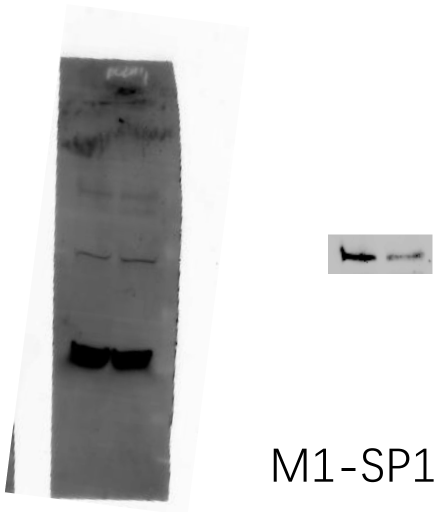

M1-SP1

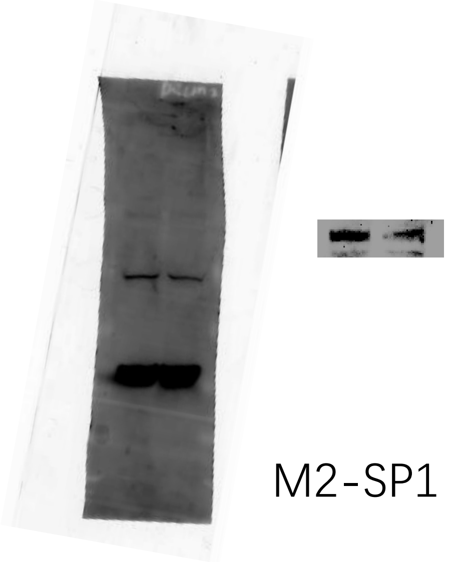

M2-SP1

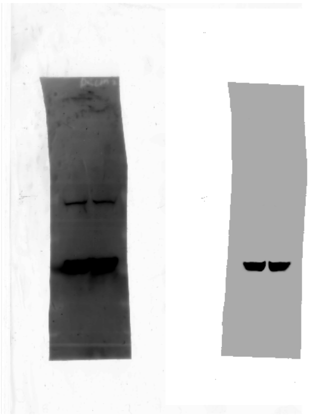

M1-actin

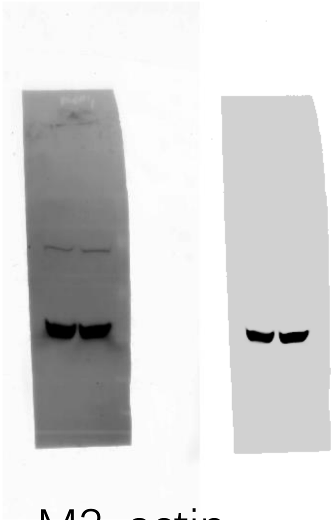

M2-actin

Figuer4 D

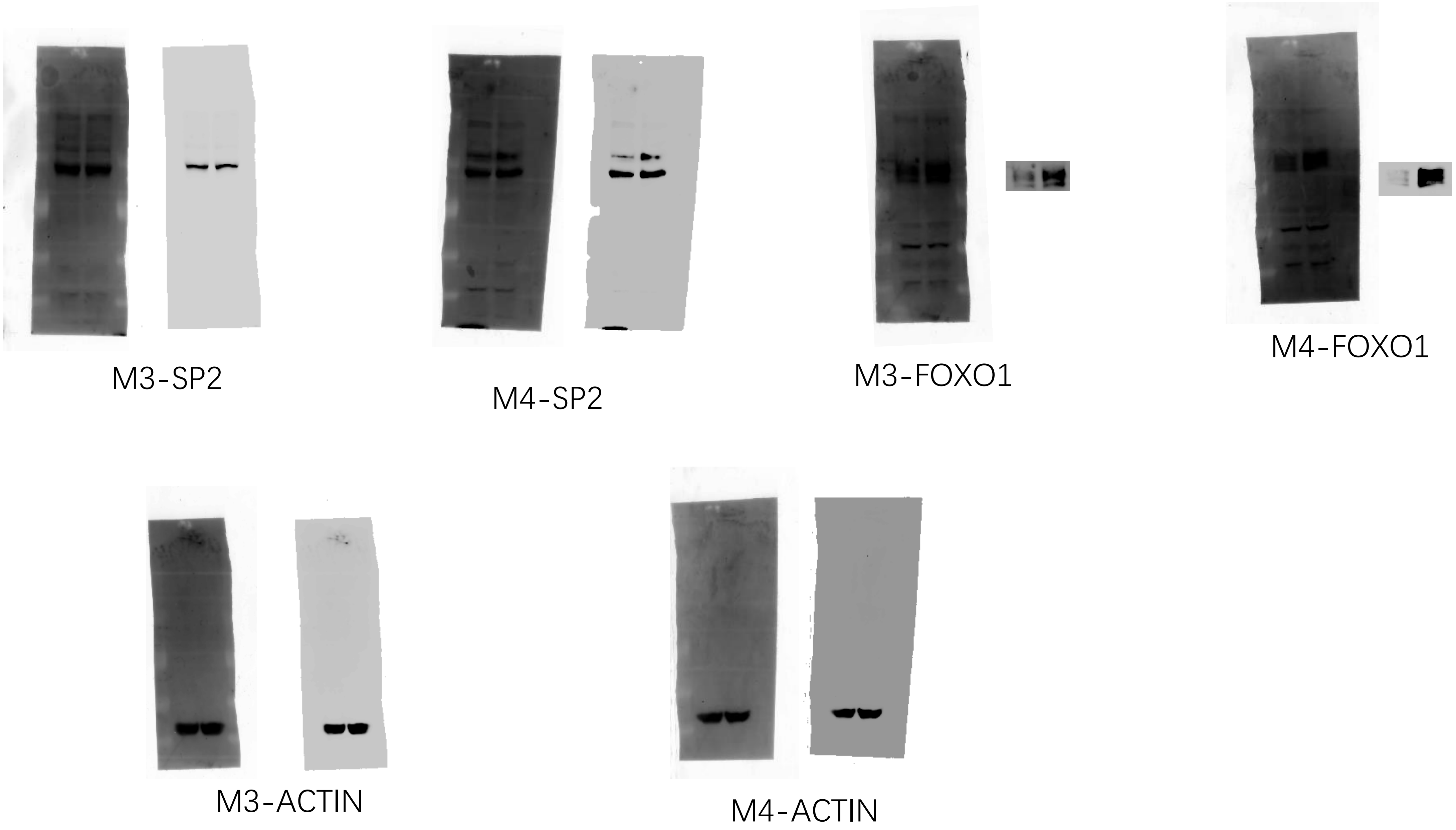

Figuer4 O

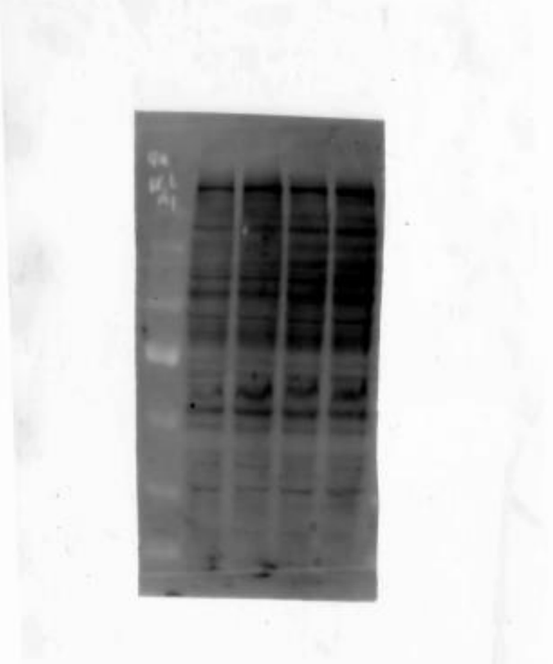

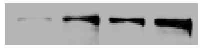

PHIP

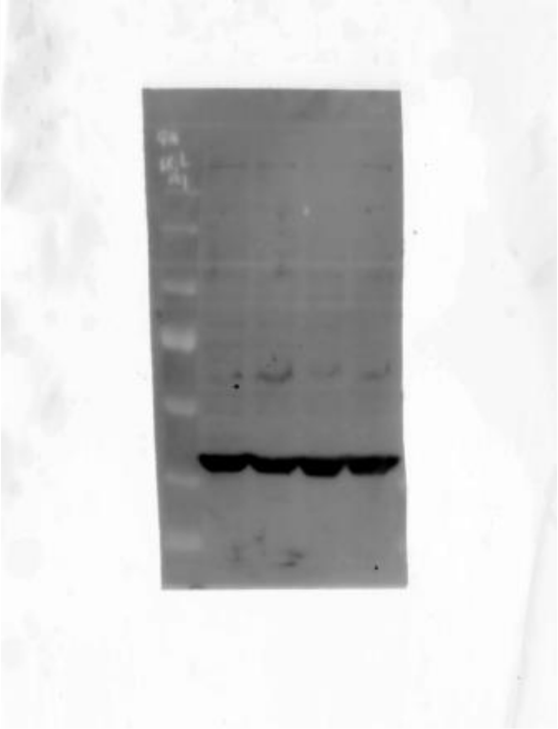

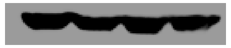

ACTIN

Figuer4 P

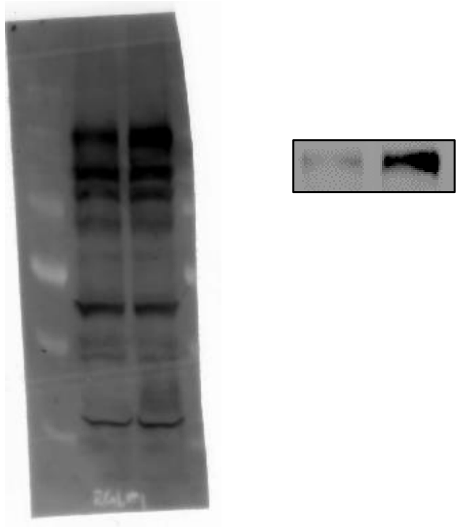

M1-DNMT3A

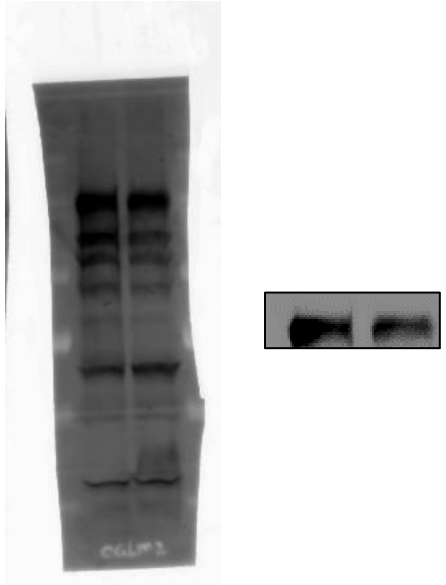

M2-DNMT3A

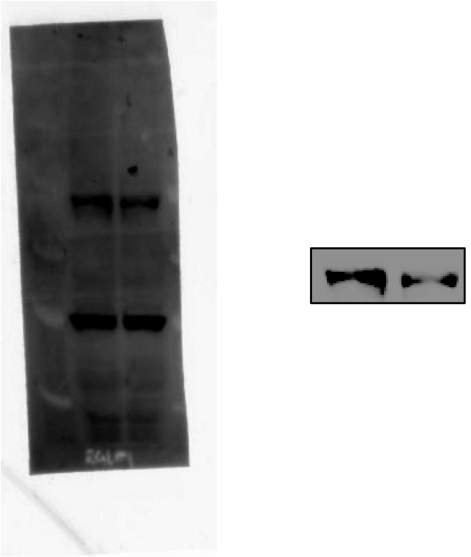

M1 DNMT3B

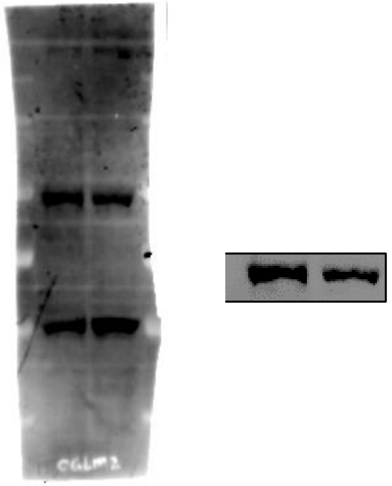

M2 DNMT3B

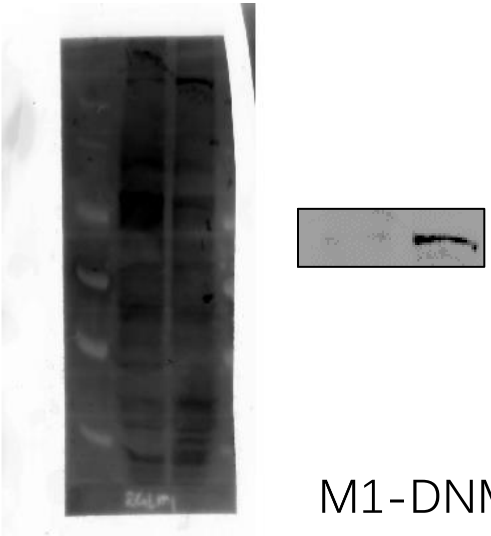

M1-DNMT1

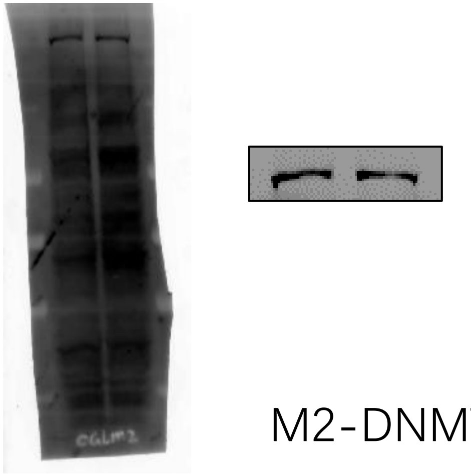

M2-DNMT1

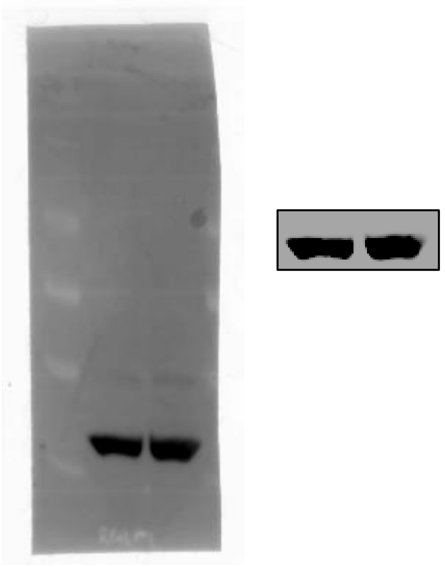

M1 B-ACTIN

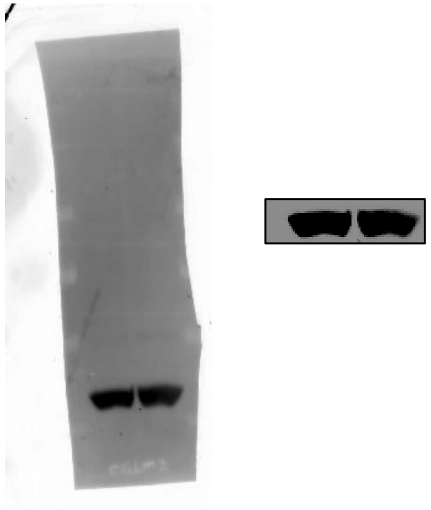

M2 B-ACTIN

Figuer4 Q

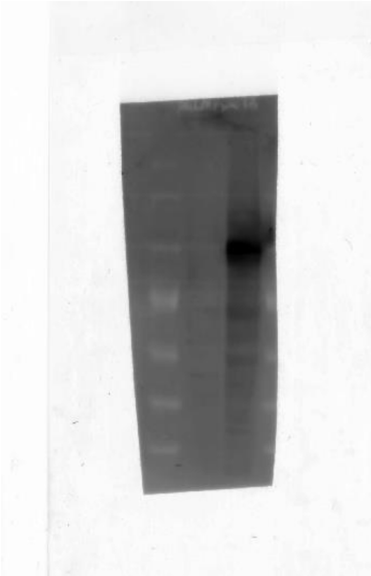

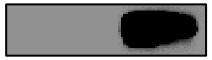

HA

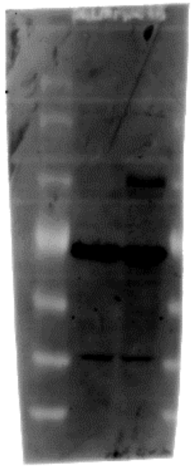

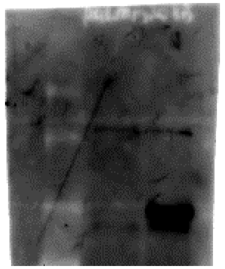

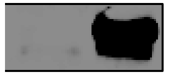

DNMT3B

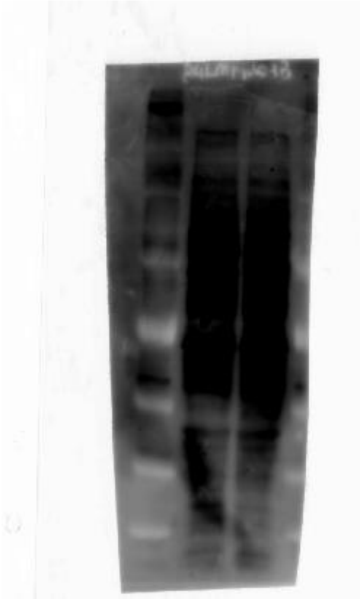

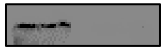

PHIP

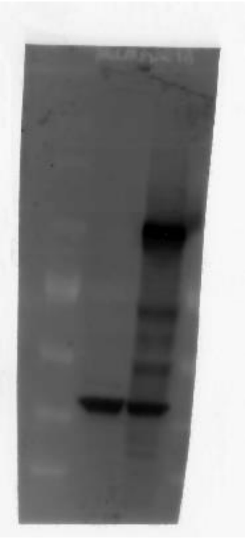

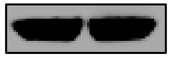

ACTIN

Figuer4 R

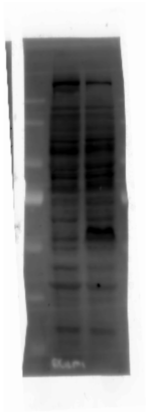

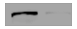

PHIP

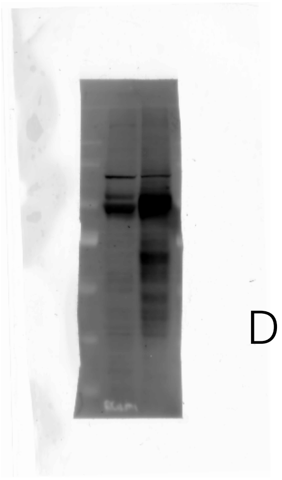

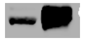

DNMT3B

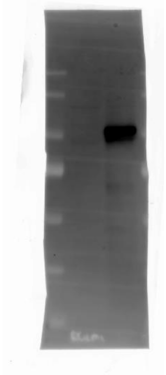

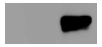

HA

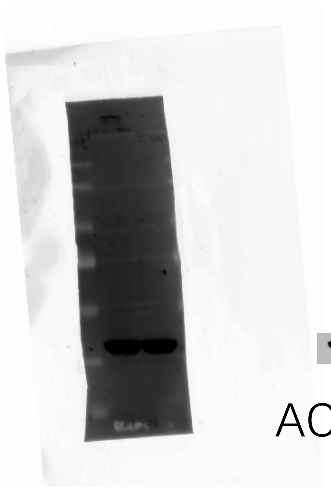

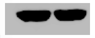

ACTIN

Figuer5 D

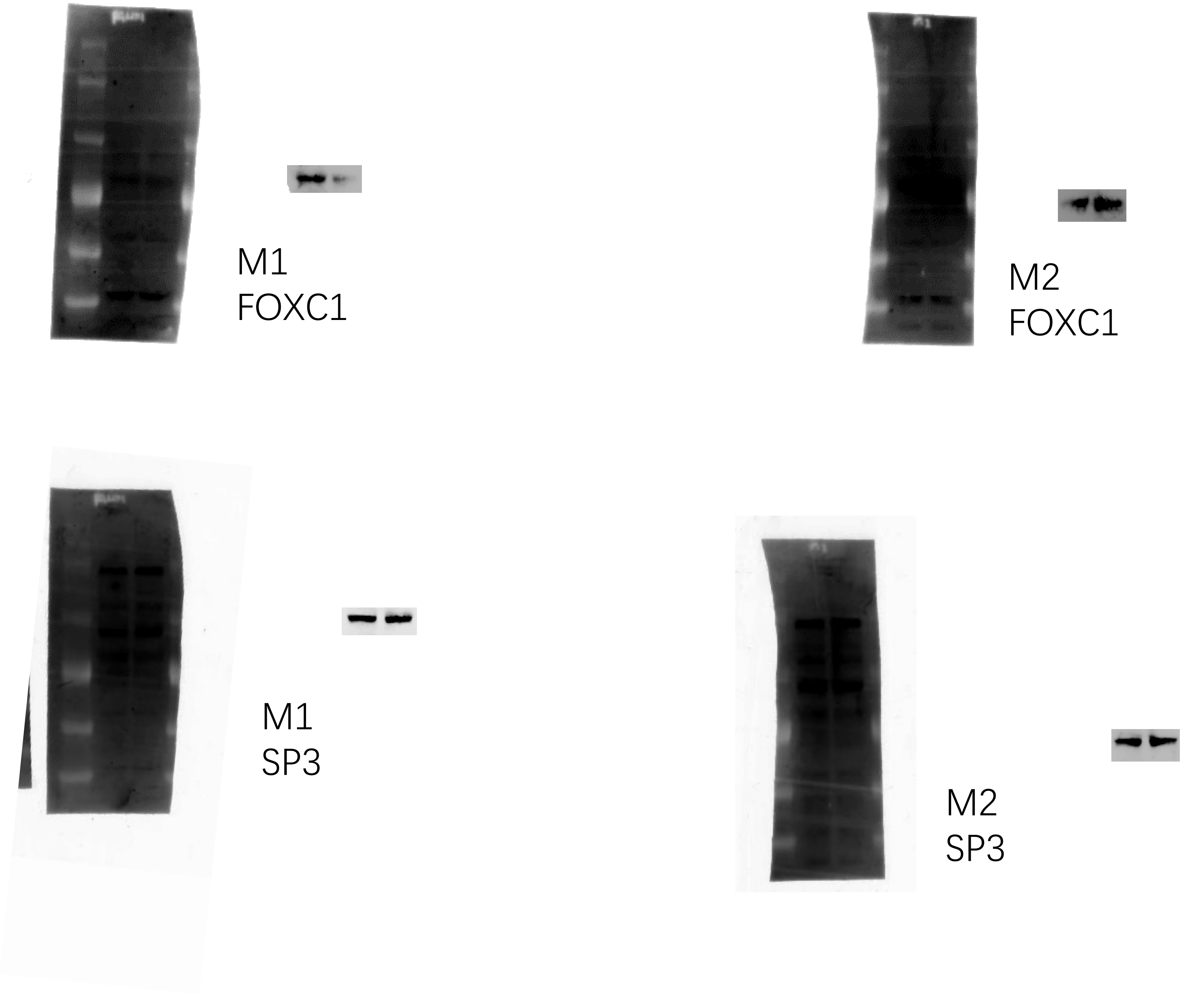

Figuer5 D

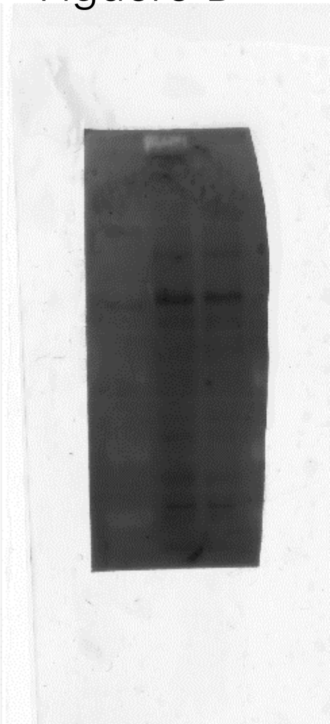

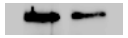

M1 SP1

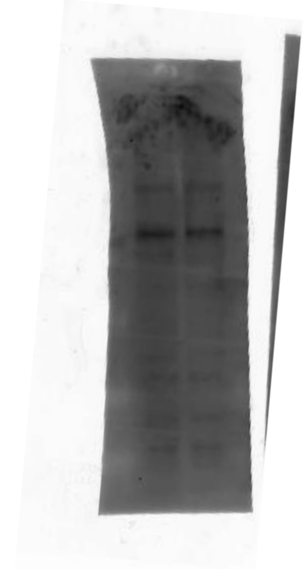

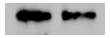

M2 SP1

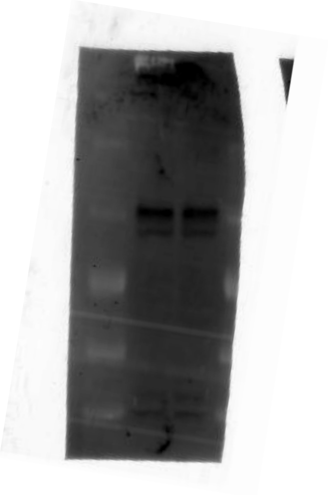

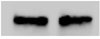

M1 FOXO3A

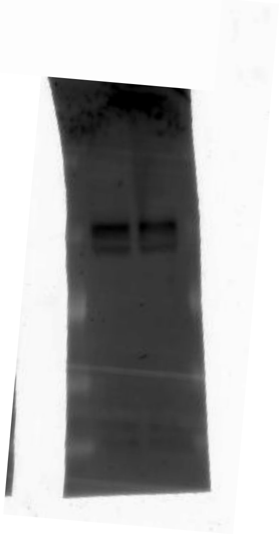

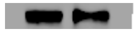

M2 FOXO3A

Figuer5 D

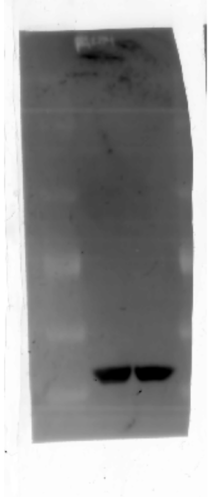

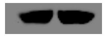

M1 ACTIN

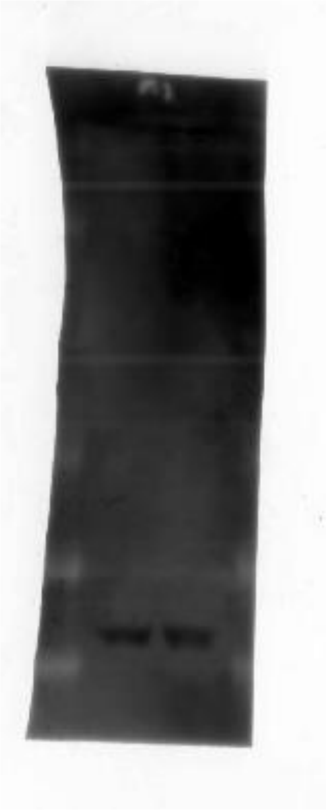

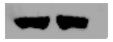

M2 ACTIN

Figuer5 E

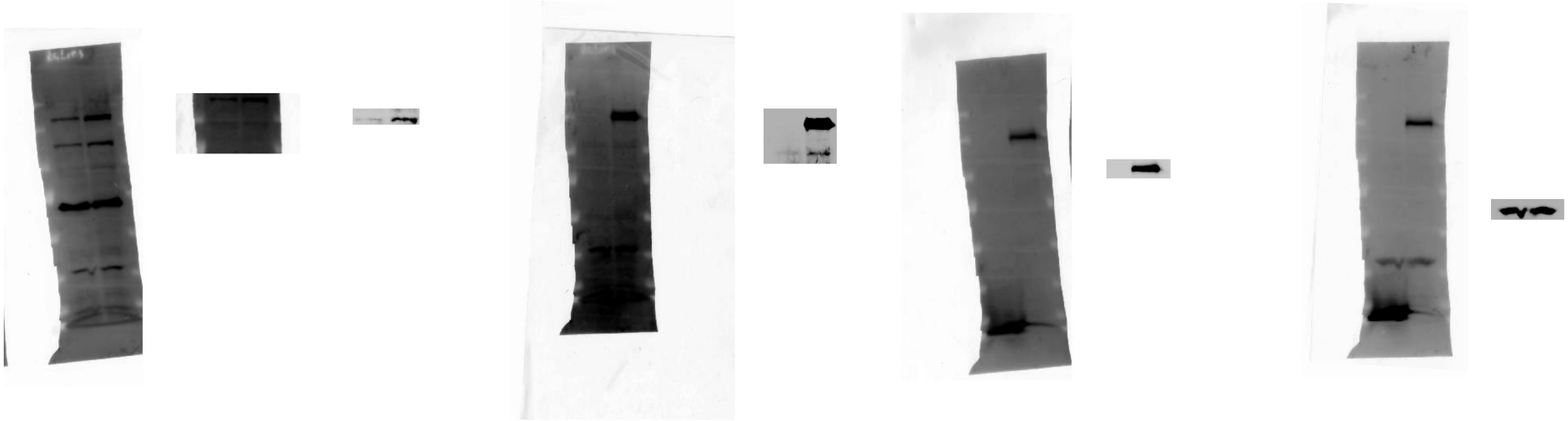

DNMT3B

SP1

GFP

β-actin

Figuer5 F

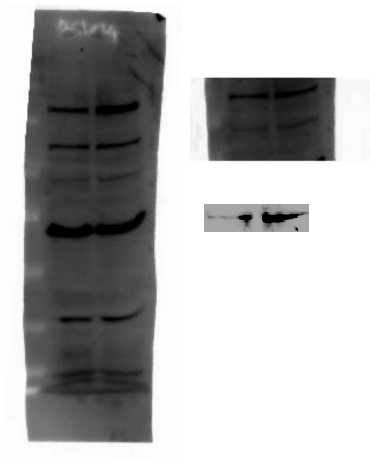

DNMT3B

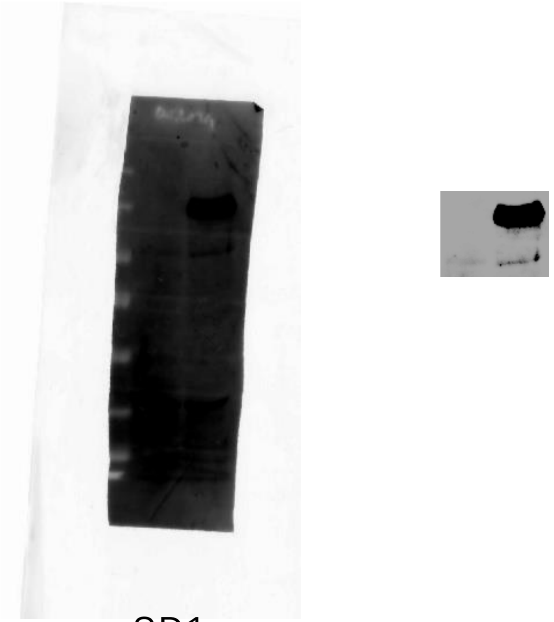

SP1

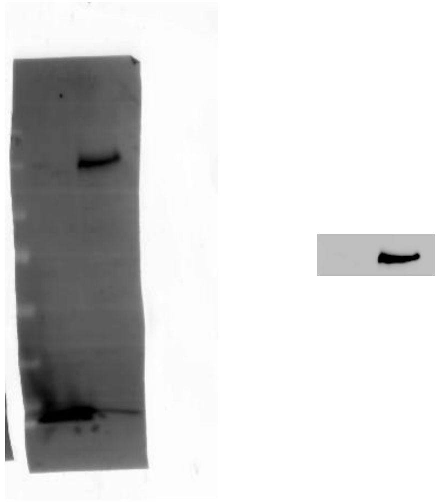

GFP

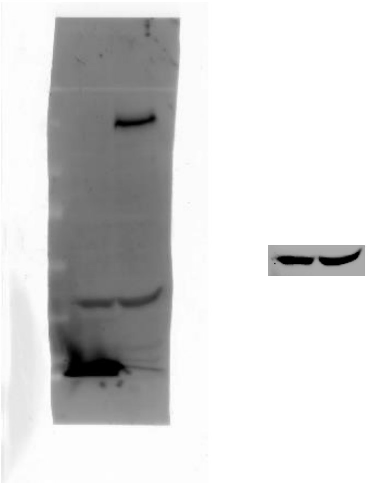

β-actin

116-SP1 蛋白降解

Figuer6 B

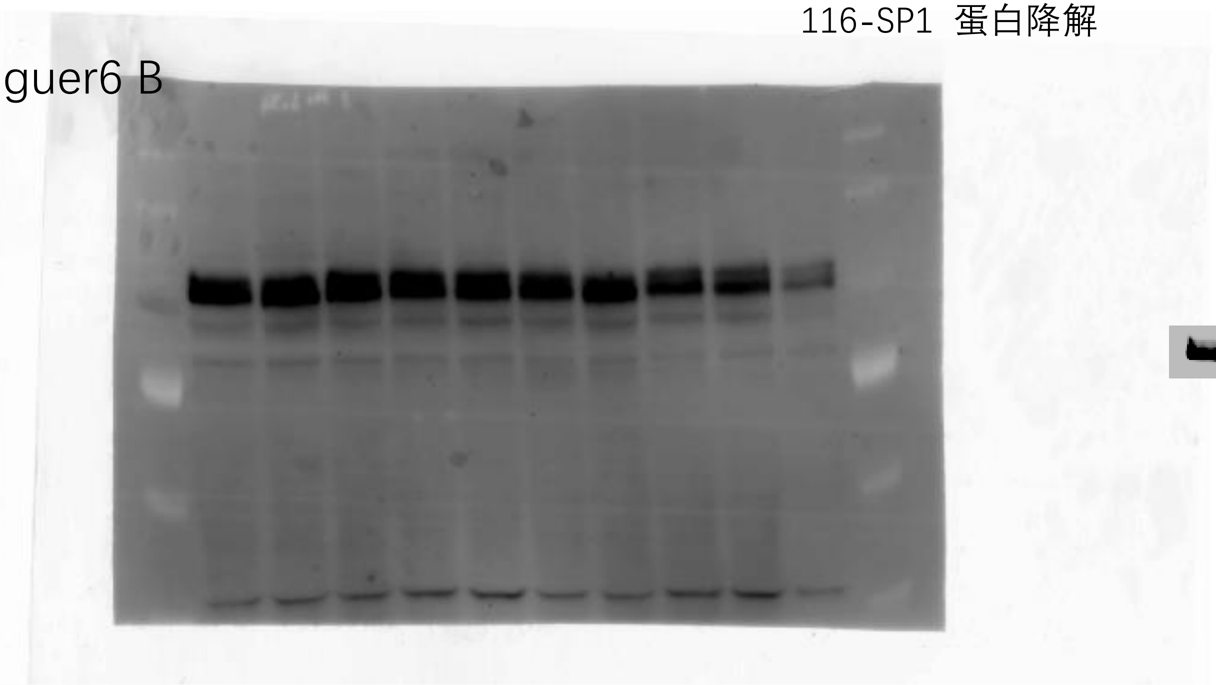

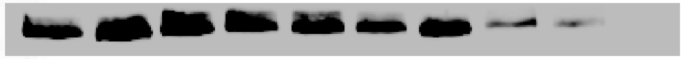

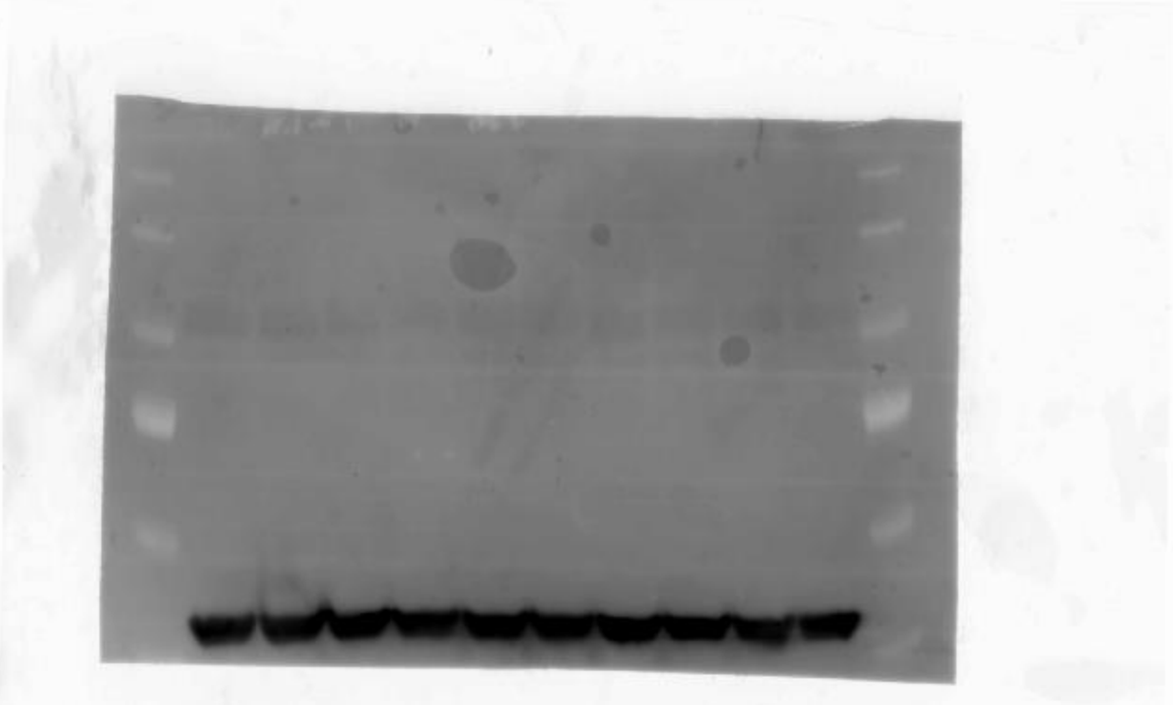

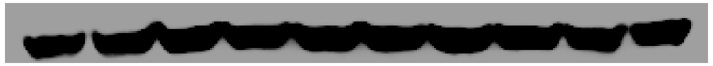

Figuer6 C

RKO-SP1 蛋白降解

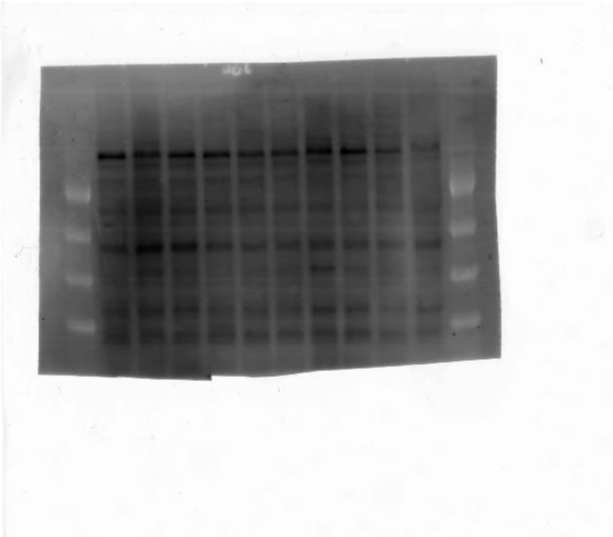

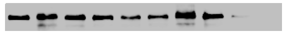

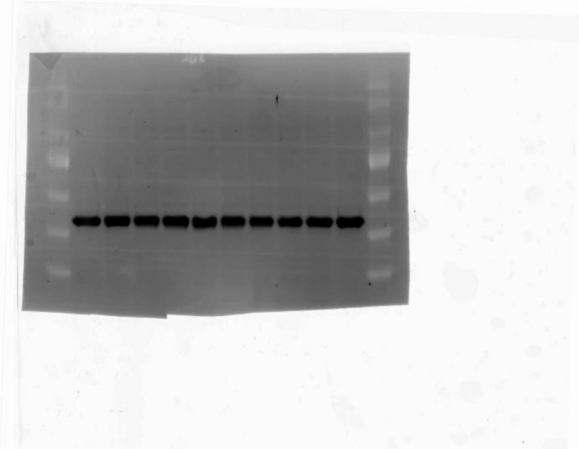

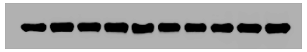

Figuer6 D

HCT116

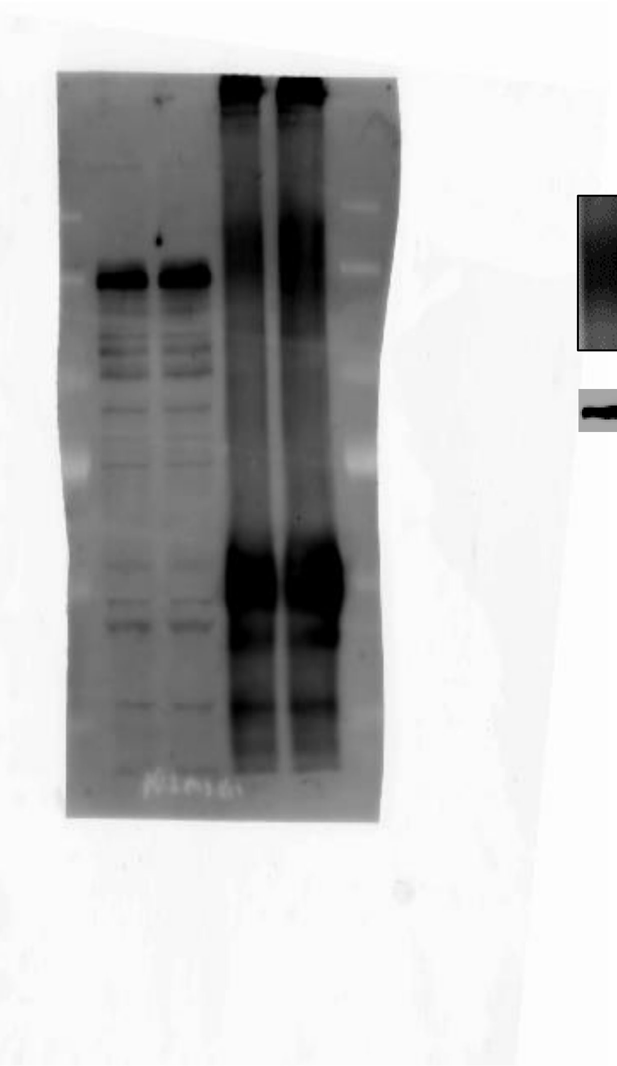

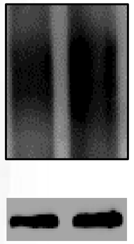

Figuer6 E

RKO

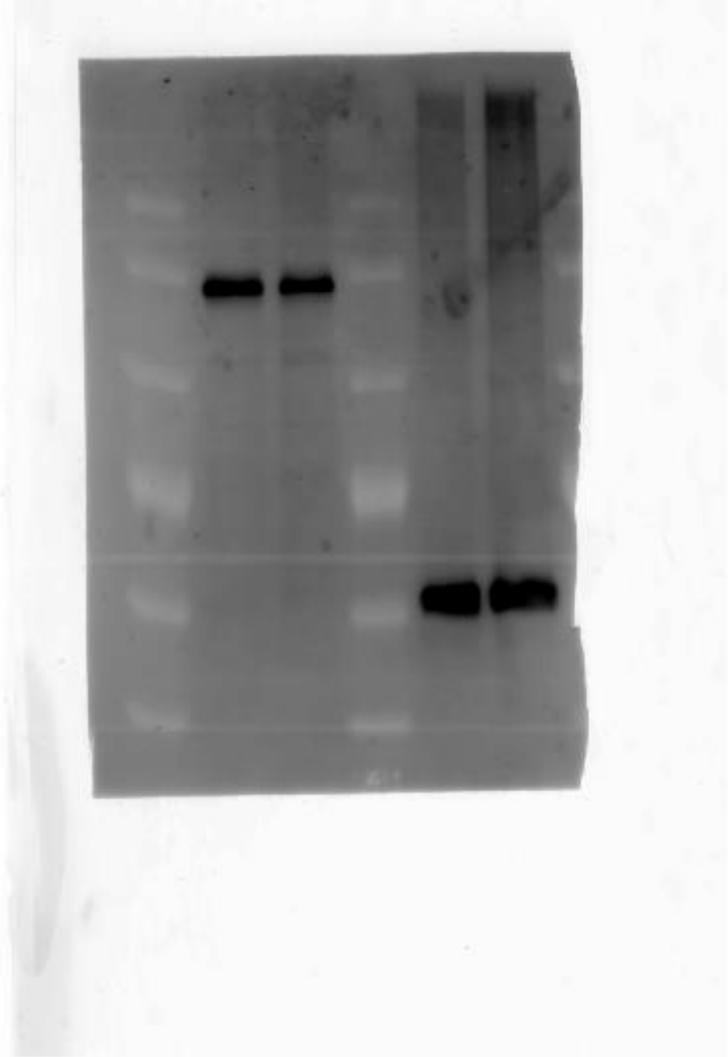

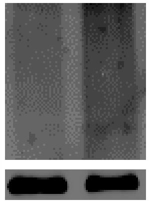

Figuer6 I

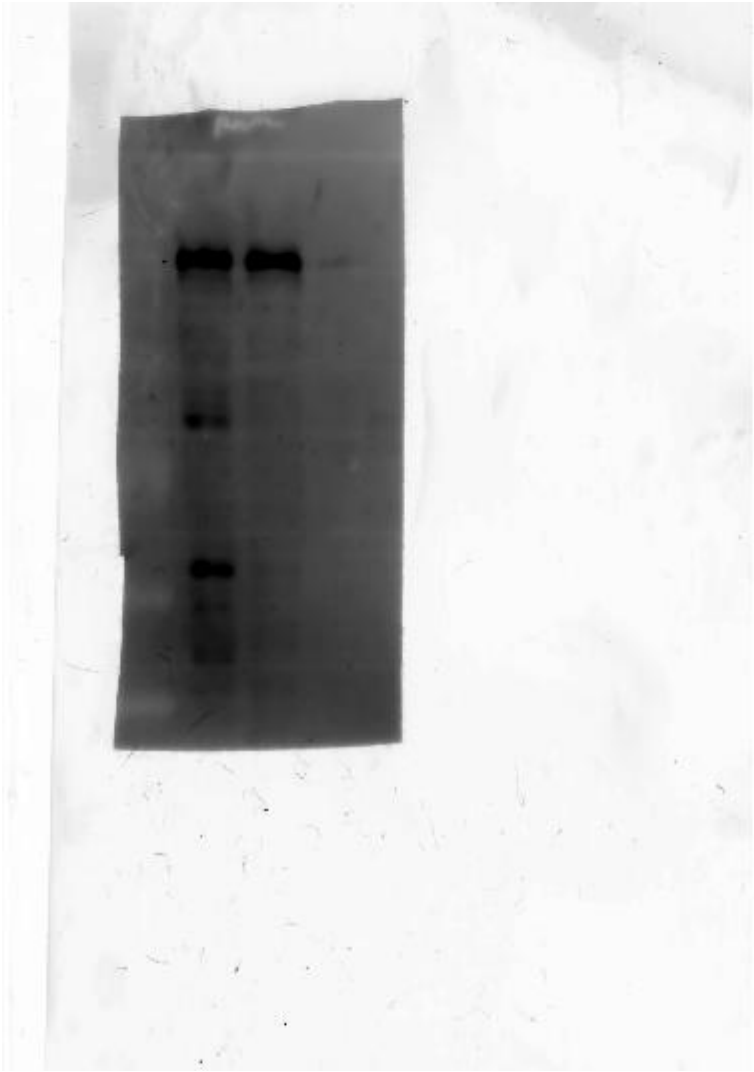

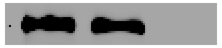

SP1

Figuer6 J

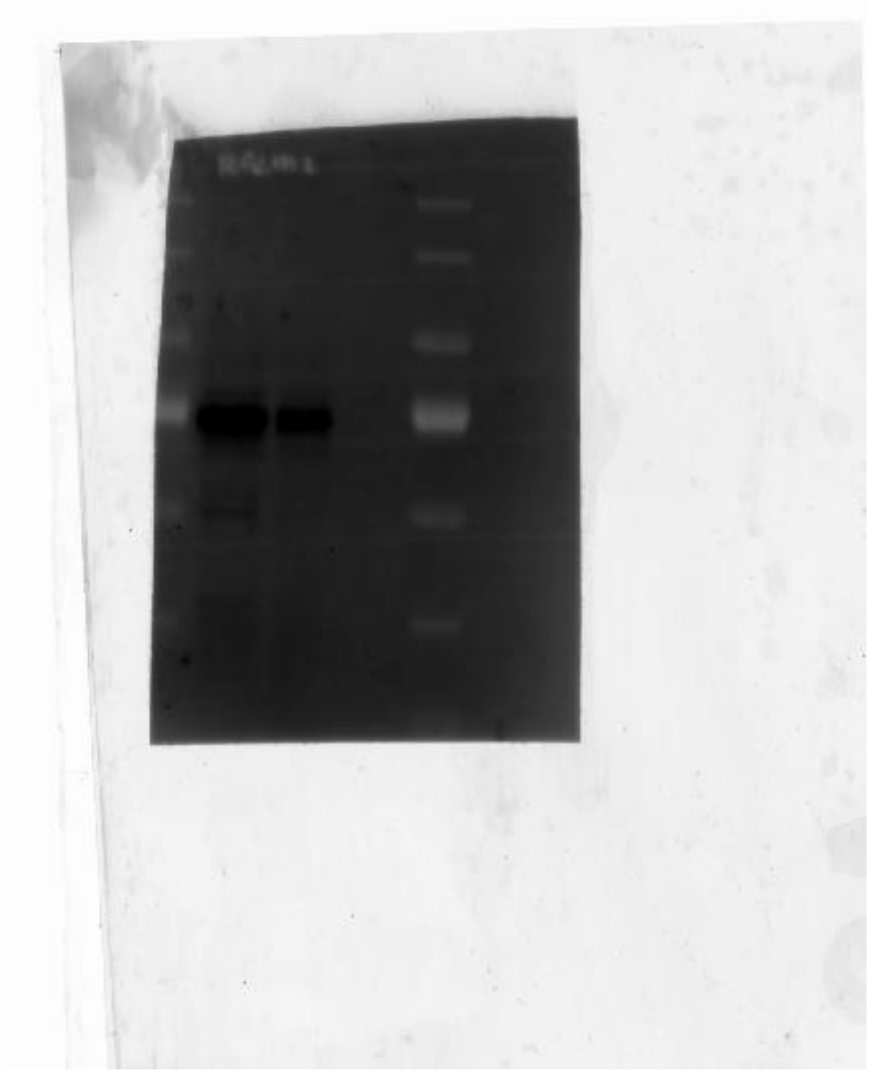

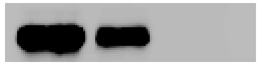

TRIM25

Figuer6 O

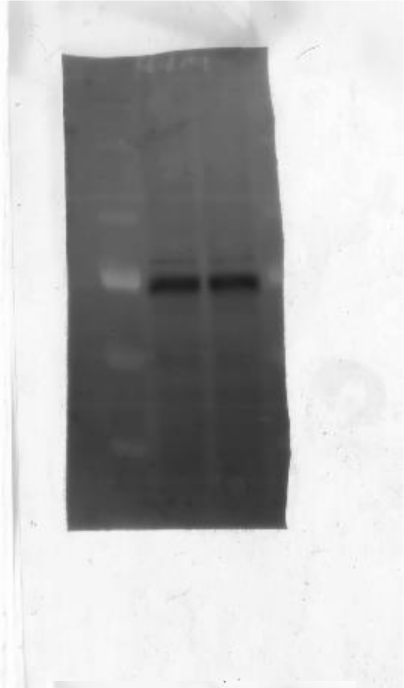

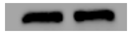

TRIM25

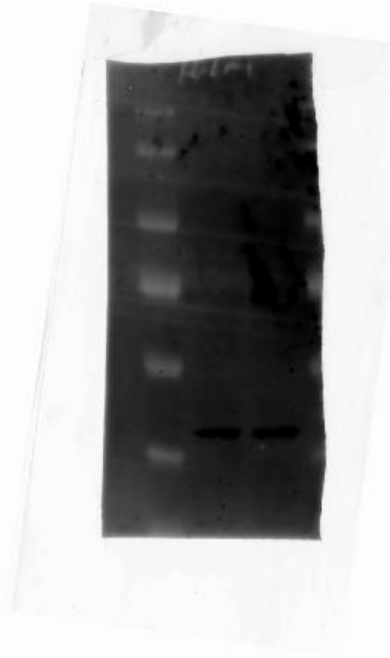

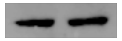

β-actin

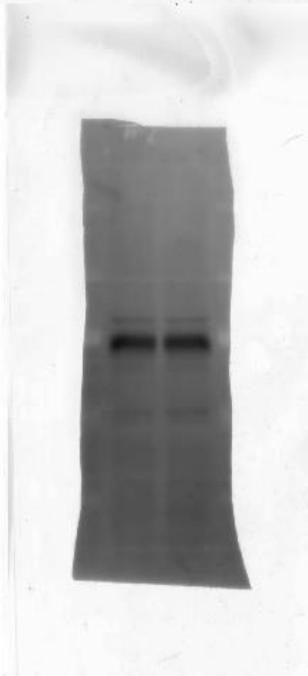

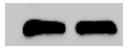

TRIM25

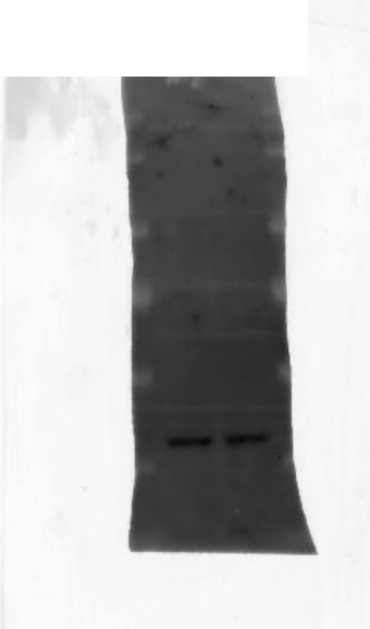

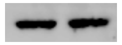

β-actin

Figuer6 P

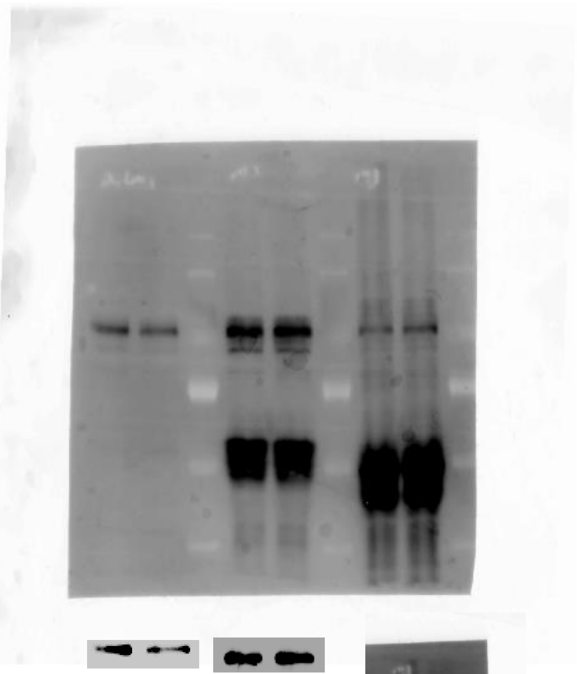

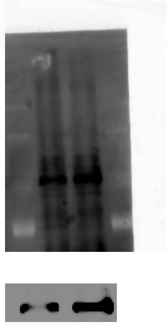

SP1

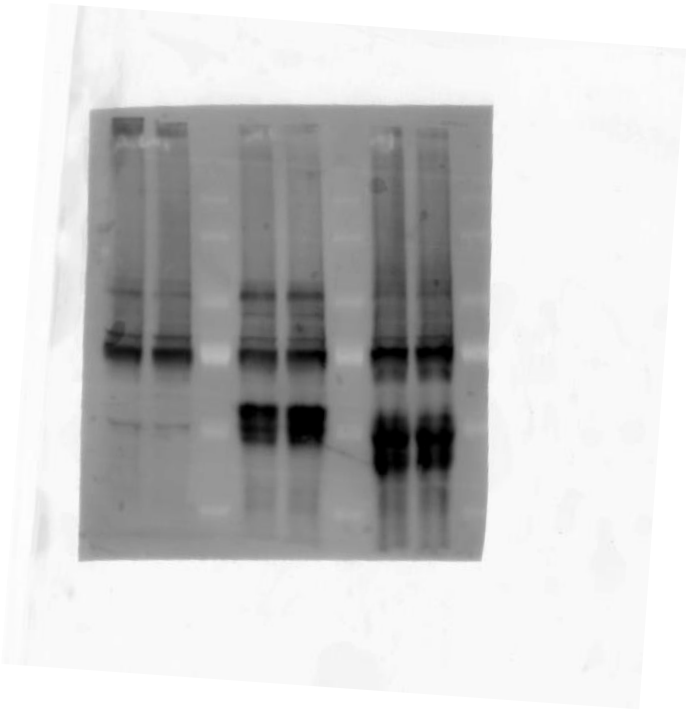

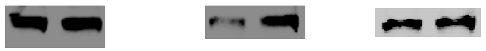

TRIM25

IP互拉 SP1 TRIM25 上抗体SP1

Figuer6 Q

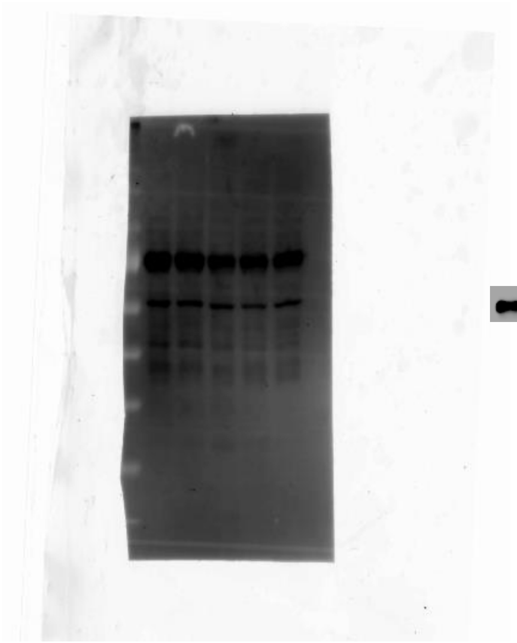

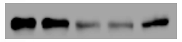

TRIM25

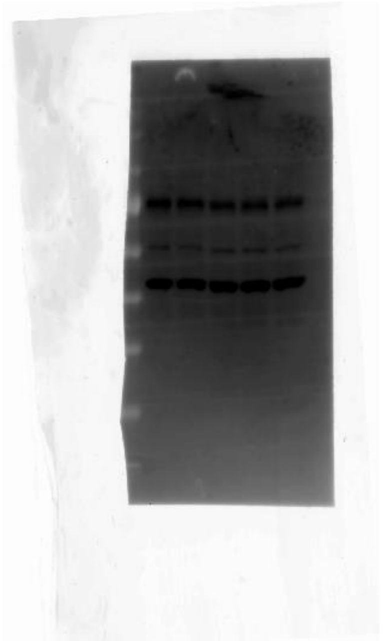

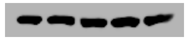

β-actin

TRIM25敲低 HCT116

Figuer6 R

RKO

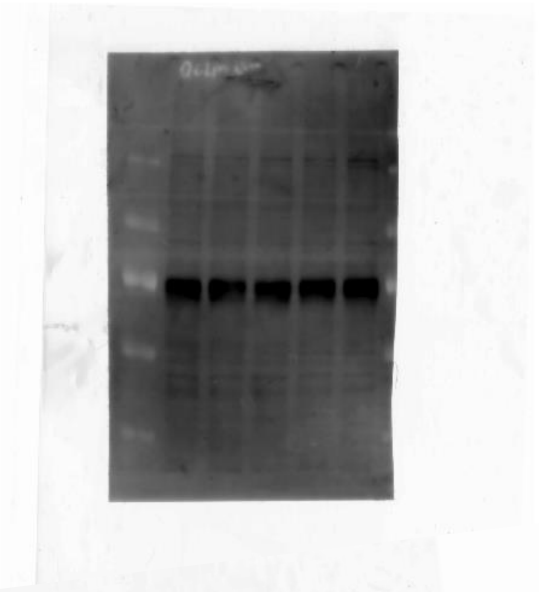

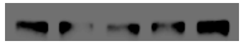

TRIM25

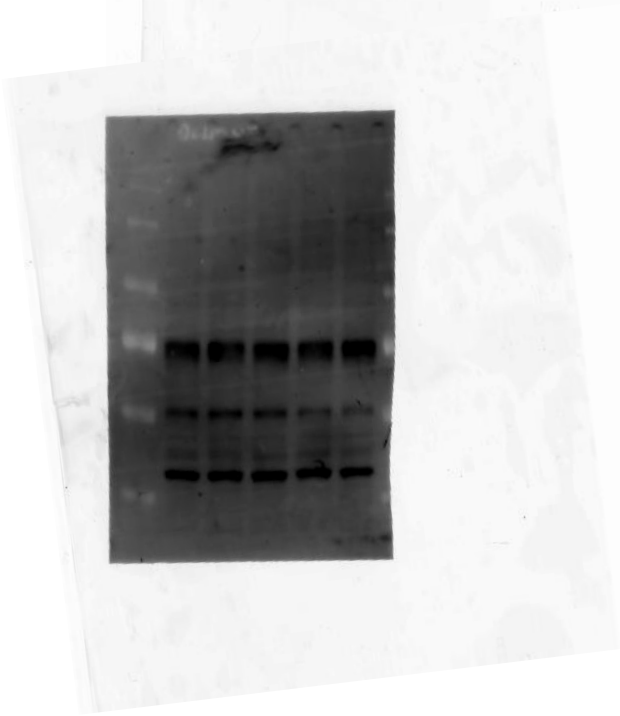

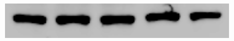

β-actin

Figuer6 S

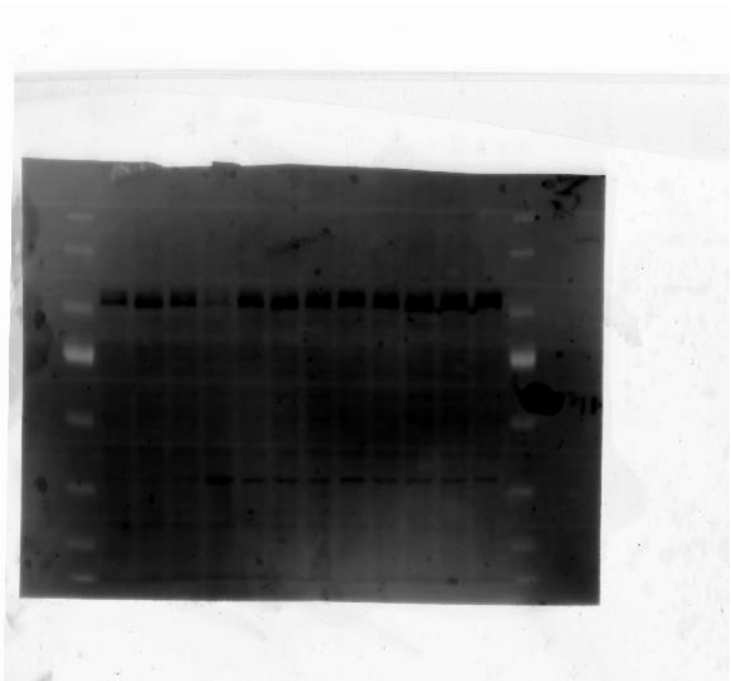

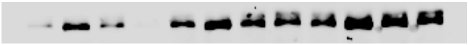

SP1

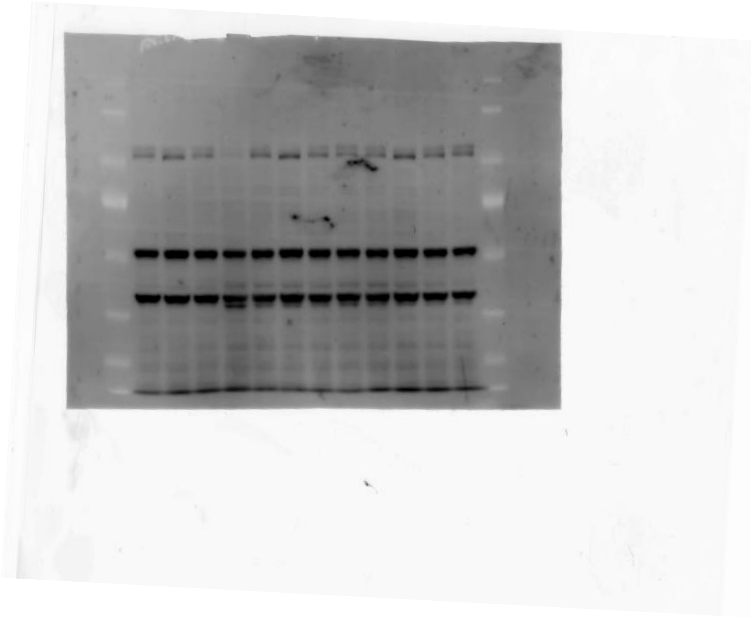

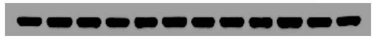

tubulin

Figuer6 T

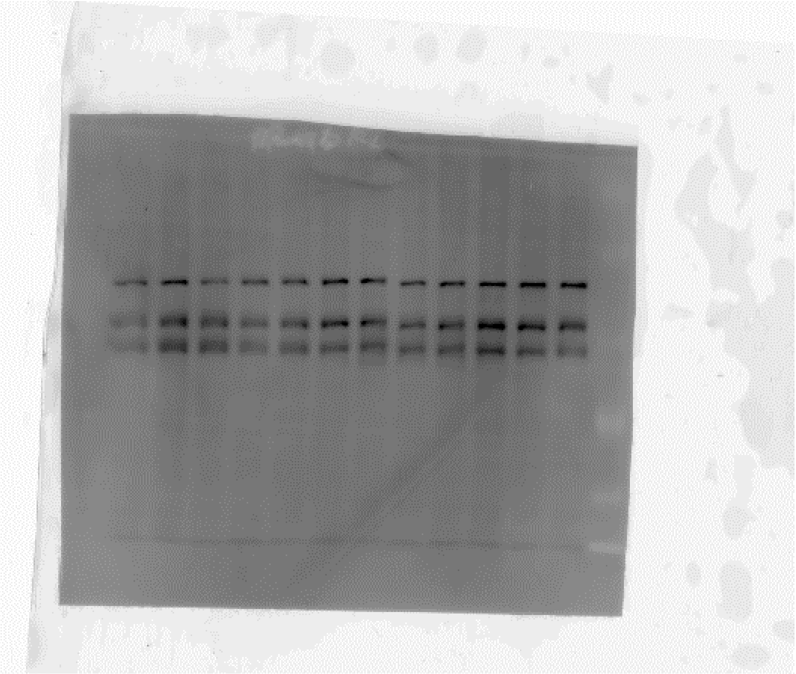

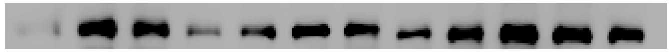

SP1

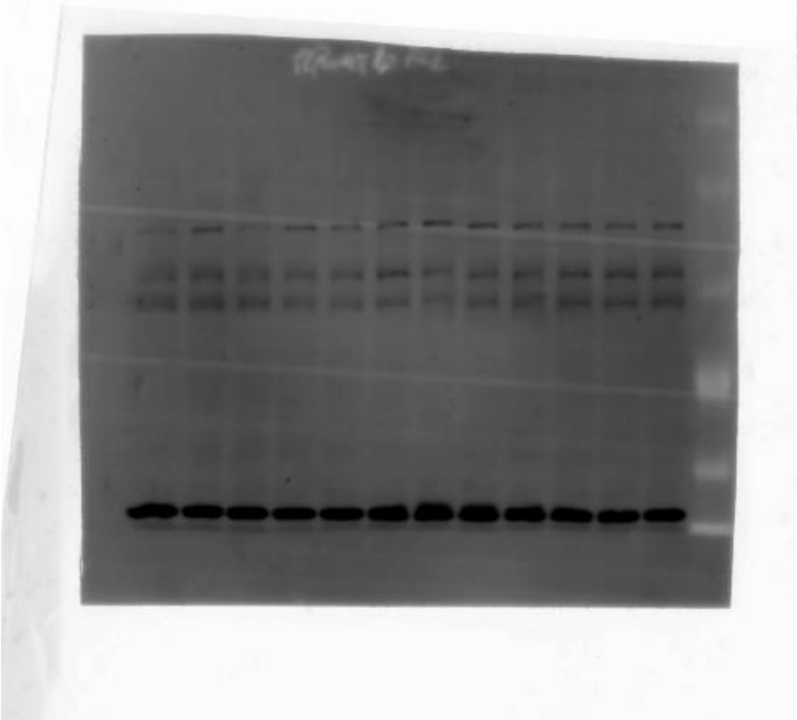

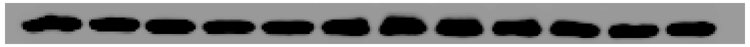

β-actin

Figuer6 U

HCT116

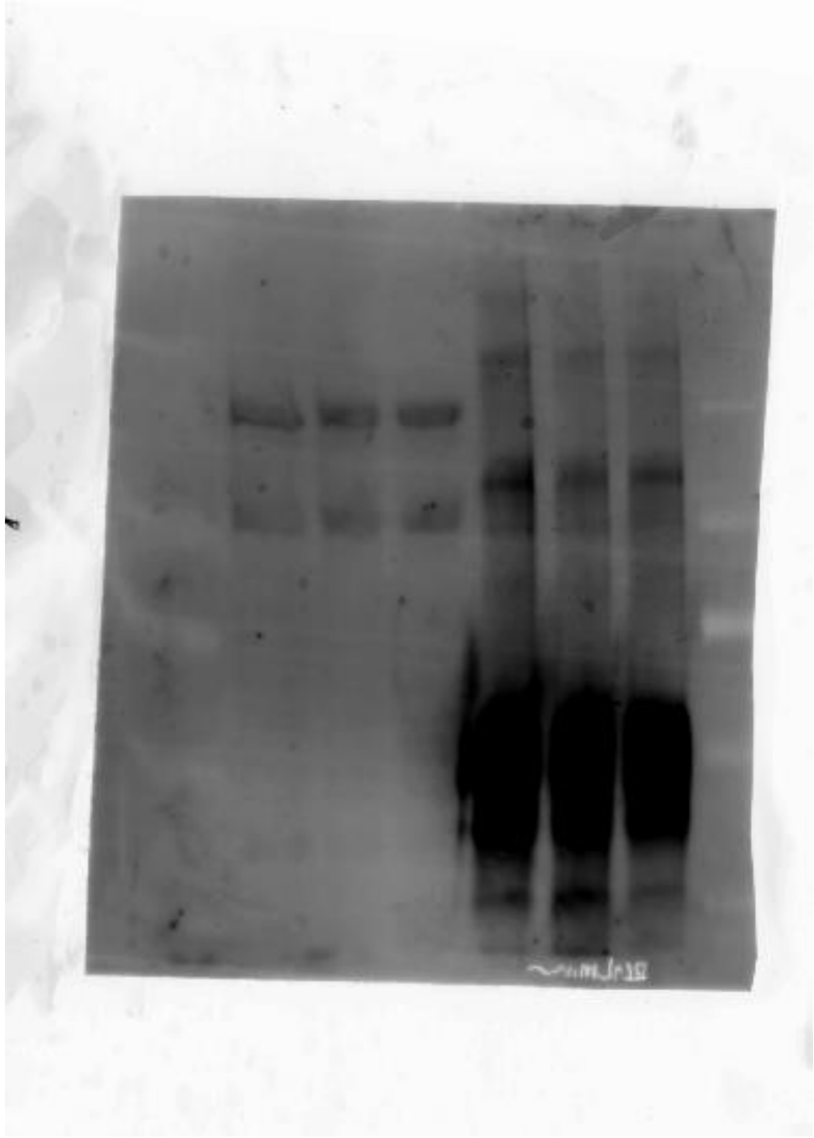

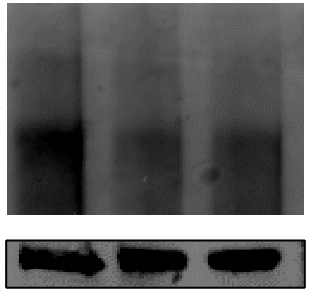

Figuer6 V

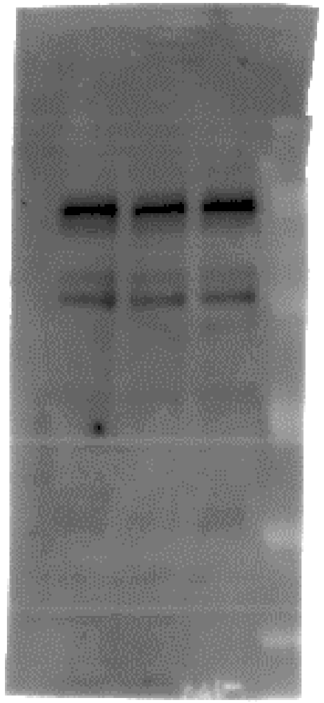

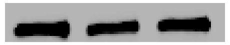

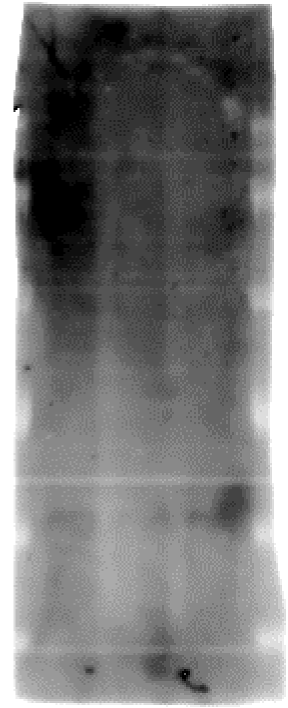

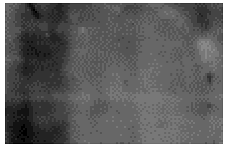

Figuer7 C

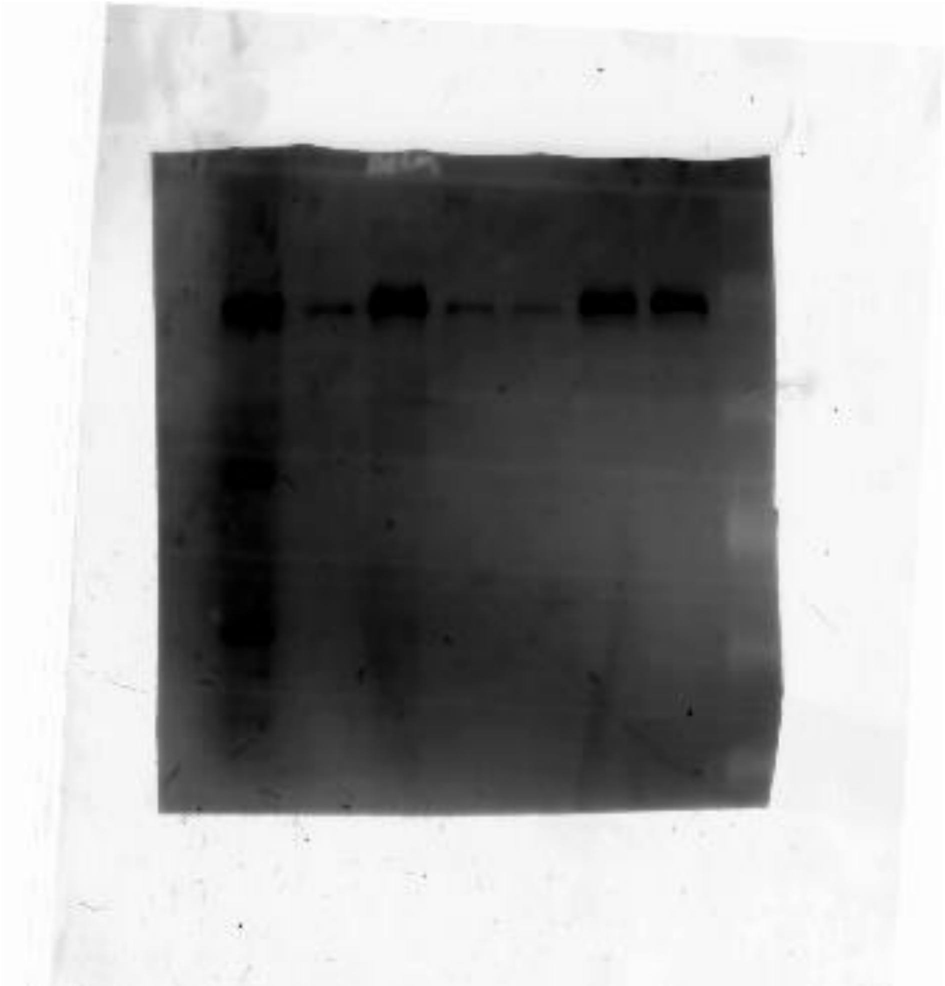

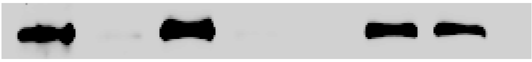

GFP-SP1

Figuer7 D

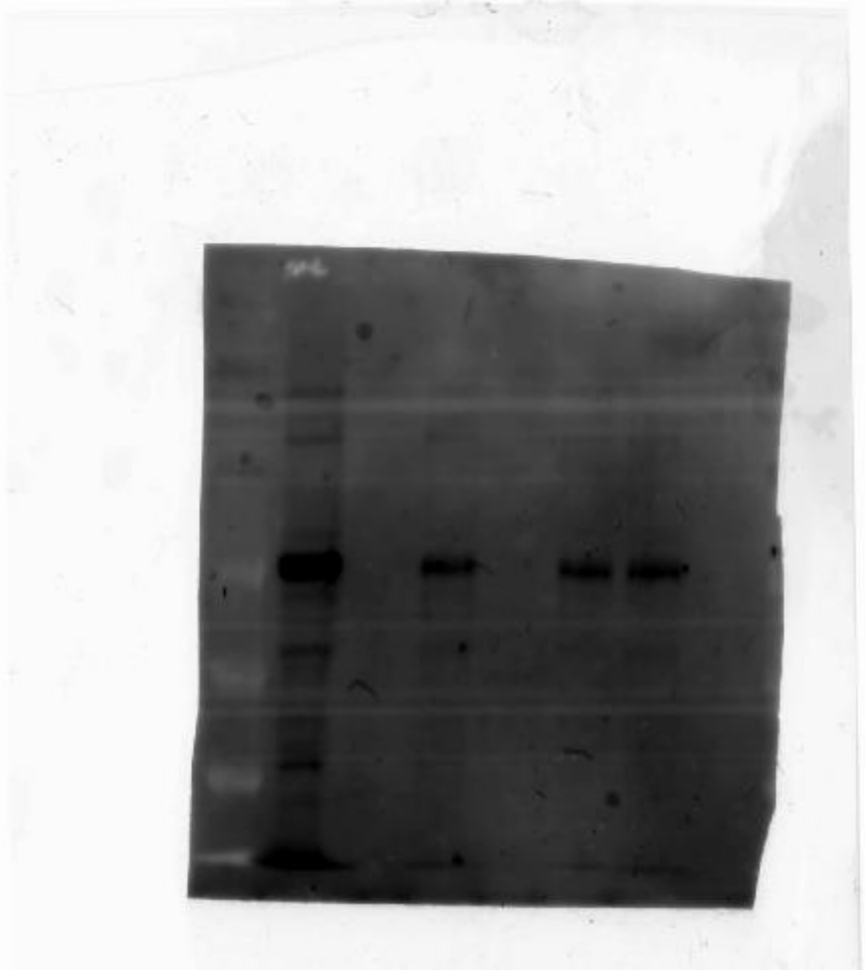

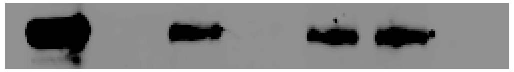

HA-TRIM25

Figuer7 H

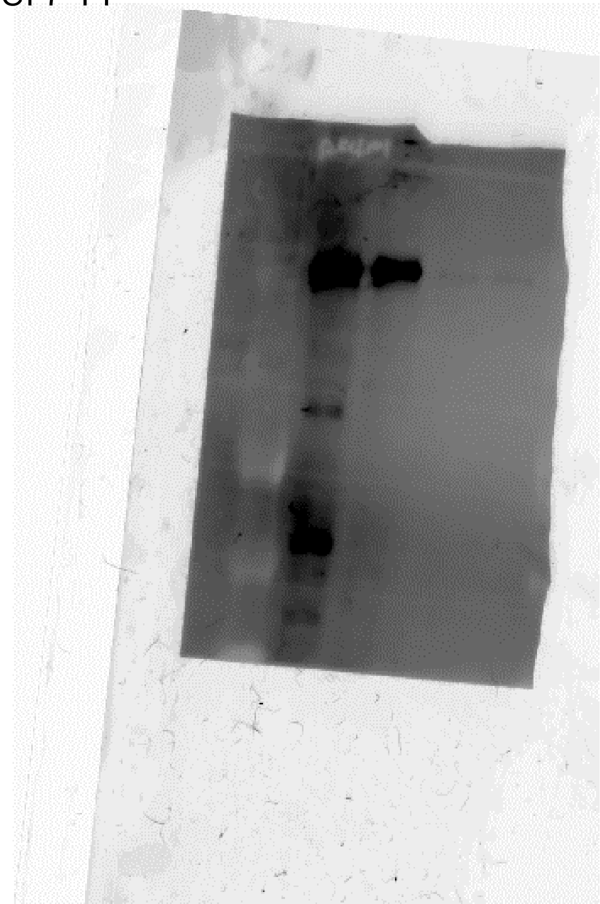

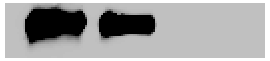

GFP-SP1

Figuer7 I

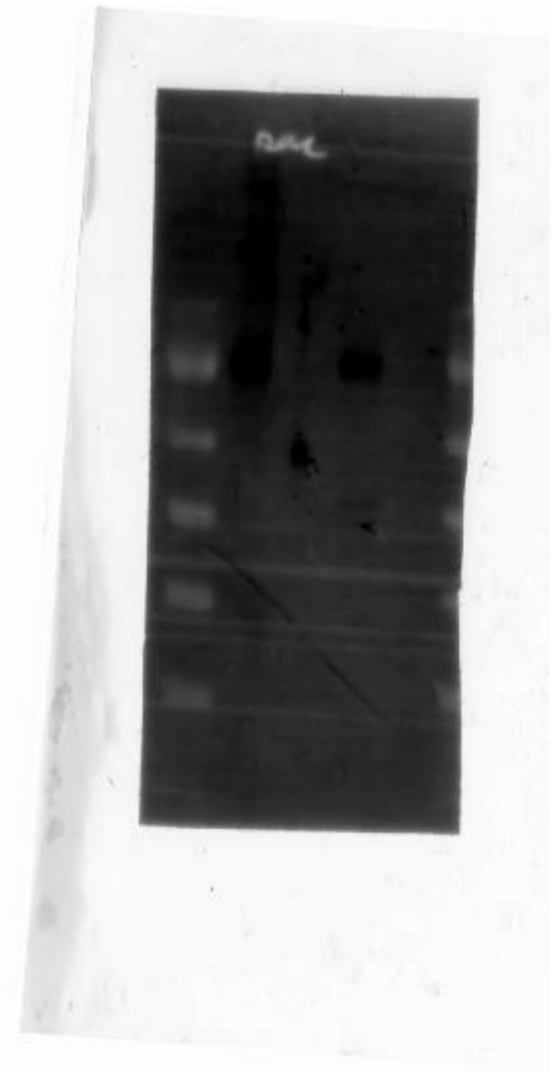

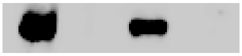

HA-TRIM25

Figuer7 J

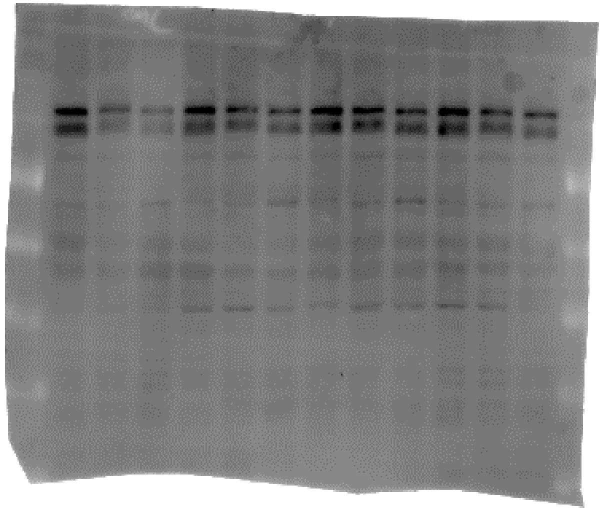

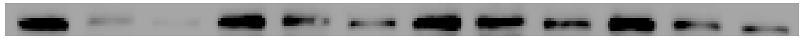

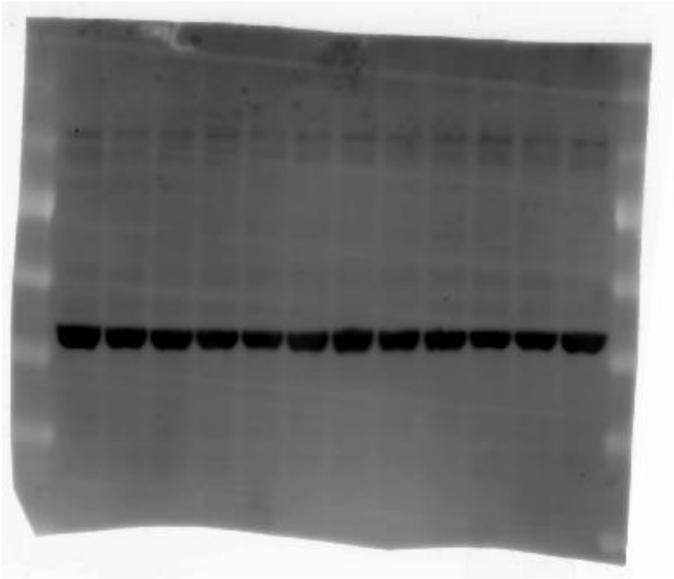

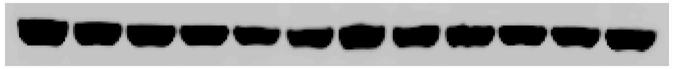

Figuer7 K

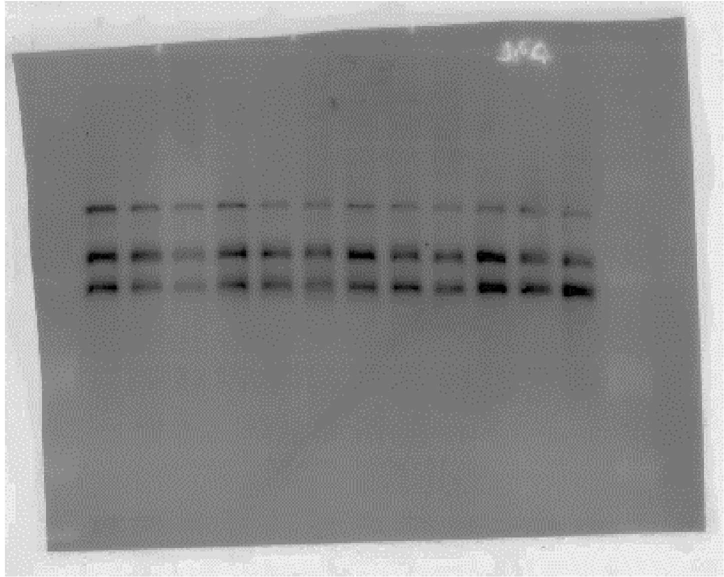

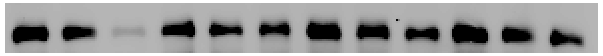

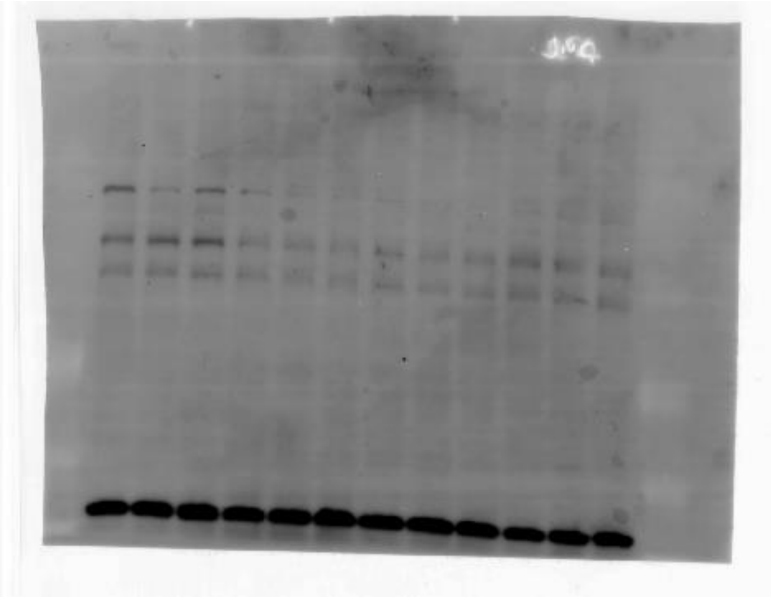

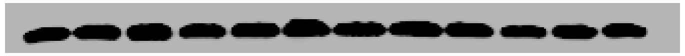

Figuer7 L

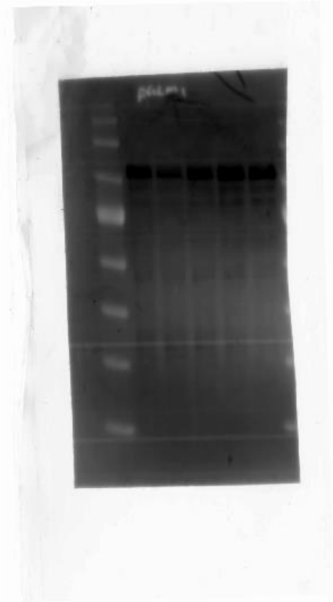

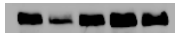

SP1

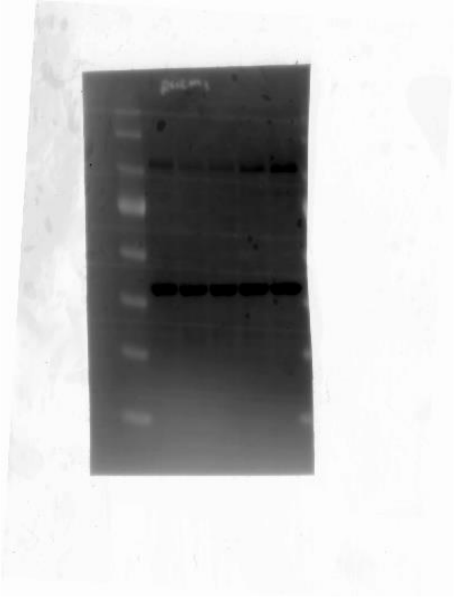

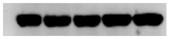

ACTIN

Figuer7 M

RKO

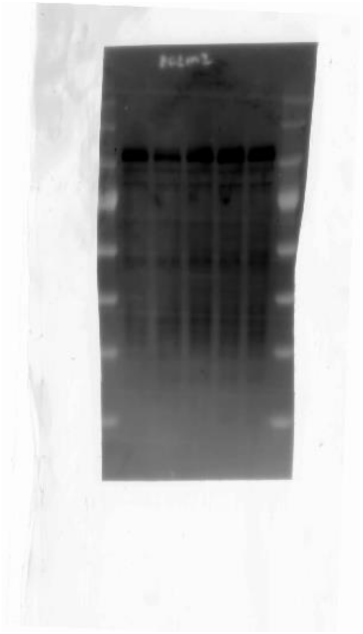

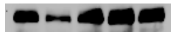

SP1

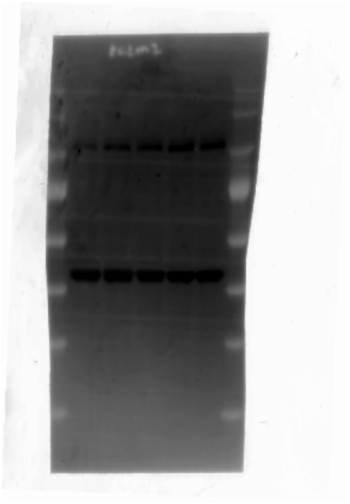

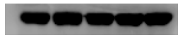

ACTIN
