# Supplementary material for: LINC00955 suppresses colorectal cancer growth by acting as a molecular scaffold of TRIM25 and Sp1 to Inhibit DNMT3B-mediated methylation of the PHIP promoter
Source: BMC Cancer. 2023 Sep 23;23:898. doi: 10.1186/s12885-023-11403-2 (PMC10518100; doi:10.1186/s12885-023-11403-2)
Supplement: Supplementary file 2 — Additional file 2: Figure S1. (A) Construction of A truncated PHIP promoter-driven luciferase reporter. (B, C) The PHIP-1 promoter-driven luciferase reporters and the PHIP-2 promoter-driven luciferase reporters were transferred into HCT116 (Vector, LINC00955) and RKO (Vector, LINC00955) cells, respectively, and their promoter activity was measured. Figure S2. (A, B) Stable transfection efficiency after deletion the LINC00955 fragment bound to Sp1 and TRIM25 in HCT116 and RKO cells, as determined by qPCR. [file 12885_2023_11403_MOESM2_ESM.docx]

**Figure S1.** (A) Construction of A truncated PHIP promoter-driven luciferase reporter. (B, C) The *PHIP-1* promoter-driven luciferase reporters and the *PHIP-2* promoter-driven luciferase reporters were transferred into HCT116 (Vector, LINC00955) and RKO (Vector, LINC00955) cells, respectively, and their promoter activity was measured.

**Figure S2.** (A, B) Stable transfection efficiency after deletion the LINC00955 fragment bound to Sp1 and TRIM25 in HCT116 and RKO cells, as determined by qPCR.
