# Supplementary material for: LINC00955 suppresses colorectal cancer growth by acting as a molecular scaffold of TRIM25 and Sp1 to Inhibit DNMT3B-mediated methylation of the PHIP promoter
Source: BMC Cancer. 2023 Sep 23;23:898. doi: 10.1186/s12885-023-11403-2 (PMC10518100; doi:10.1186/s12885-023-11403-2)
Supplement: Supplementary file 3 — Additional file 3: Table S1. Information about CRC patients including case number, gender, age and tumor largest diameter. [file 12885_2023_11403_MOESM3_ESM.docx]

| **case(NO#)** | **gender** | **age** | **Tumor diameter(cm)** | **case(NO#)** | **gender** | **age** | **Tumor diameter(cm)** |
| --- | --- | --- | --- | --- | --- | --- | --- |
| **1#** | **female** | **52** | **4** | **39#** | **male** | **64** | **2.5** |
| **2#** | **male** | **47** | **6** | **40#** | **male** | **67** | **4** |
| **3#** | **male** | **75** | **6** | **41#** | **male** | **69** | **N/A** |
| **4#** | **male** | **79** | **4** | **42#** | **male** | **54** | **5** |
| **5#** | **female** | **68** | **2** | **43#** | **female** | **86** | **3.5** |
| **6#** | **female** | **80** | **6** | **44#** | **female** | **56** | **N/A** |
| **7#** | **female** | **69** | **5** | **45#** | **female** | **52** | **7** |
| **8#** | **female** | **69** | **4** | **46#** | **female** | **79** | **3** |
| **9#** | **female** | **74** | **2** | **47#** | **male** | **71** | 4 |
| **10#** | **male** | **61** | **2** | **48#** | **male** | **43** | **5** |
| **11#** | **male** | **81** | **3.5** | **49#** | **female** | **68** | **2** |
| **12#** | **male** | **44** | **7** | **50#** | **female** | **77** | **7** |
| **13#** | **male** | **63** | **N/A** | **51#** | **male** | **73** | **4** |
| **14#** | **female** | **73** | **2.5** | **52#** | **female** | **49** | **2** |
| **15#** | **female** | **77** | **N/A** | **53#** | **male** | **70** | **2** |
| **16#** | **female** | **75** | **2.8** | **54#** | **female** | **69** | **8** |
| **17#** | **male** | **81** | **4.5** | **55#** | **male** | **73** | **1.5** |
| **18#** | **male** | **53** | **3.5** | **56#** | **male** | **75** | **1.5** |
| **19#** | **female** | **45** | **5** | **57#** | **male** | **69** | **9.5** |
| **20#** | **male** | **57** | **2.8** | **58#** | **male** | **65** | **4** |
| **21#** | **male** | **84** | **4** | **59#** | **male** | **79** | **4** |
| **22#** | **female** | **69** | **5** | **60#** | **male** | **75** | **9** |
| **23#** | **male** | **68** | **5** | **61#** | **male** | **63** | **4.5** |
| **24#** | **male** | **68** | **4** | **62#** | **male** | **71** | **3** |
| **25#** | **male** | **61** | **5** | **63#** | **female** | **84** | **5** |
| **26#** | **male** | **79** | **8** | **64#** | **male** | **74** | **3** |
| **27#** | **male** | **69** | **3** | **65#** | **female** | **77** | **5** |
| **28#** | **male** | **84** | **N/A** | **66#** | **male** | **45** | **5** |
| **29#** | **female** | **69** | **4** | **67#** | **male** | **42** | **7.5** |
| **30#** | **male** | **84** | **4** | **68#** | **female** | **44** | **5** |
| **31#** | **male** | **55** | **5** | **69#** | **female** | **74** | **4** |
| **32#** | **female** | **71** | **7** | **70#** | **female** | **54** | **5** |
| **33#** | **female** | **46** | **4.5** | **71#** | **male** | **32** | **5** |
| **34#** | **female** | **48** | **4.5** | **72#** | **male** | **68** | **7** |
| **35#** | **male** | **89** | **8** | **73#** | **female** | **66** | **2.5** |
| **36#** | **male** | **62** | **3.5** | **74#** | **male** | **55** | **2.5** |
| **37#** | **male** | **73** | **4** | **75#** | **male** | **58** | **3** |
| **38#** | **male** | **68** | **3.8** |  |  |  |  |

**Table S1.** Information about CRC patients including case number, gender, age and tumor largest diameter.
